# SARS-CoV-2 reinfections with BA.1 (Omicron) variant among fully vaccinated individuals in northeastern Brazil

**DOI:** 10.1371/journal.pntd.0010337

**Published:** 2022-10-03

**Authors:** Francisco P. Freire-Neto, Diego G. Teixeira, Dayse C. S. da Cunha, Ingryd C. Morais, Celisa P. M. Tavares, Genilson P. Gurgel, Sanderson D. N. Medeiros, David C. dos Santos, Alexandre de O. Sales, Selma M. B. Jeronimo

**Affiliations:** 1 Instituto de Medicina Tropical do Rio Grande do Norte, Universidade Federal do Rio Grande do Norte, Natal, Rio Grande do Norte, Brazil; 2 Departmento de Bioquímica, Universidade Federal de São Paulo, São Paulo, São Paulo, Brazil; 3 Getúlio Sales Diagnósticos, Natal, Rio Grande do Norte, Brazil; 4 Secretaria Municipal de Saúde, Apodi, Rio Grande do Norte, Brazil; 5 Instituto Metrópole Digital, Universidade Federal do Rio Grande do Norte, Natal, Rio Grande do Norte, Brazil; 6 Departmento de Bioquímica, Centro de Biociências, Universidade Federal do Rio Grande Norte, Natal, Rio Grande do Norte, Brazil; 7 Instituto Nacional de Ciência e Tecnologia de Doenças Tropicais, Natal, Rio Grande do Norte, Brazil; Medizinische Universitat Wien, AUSTRIA

## Abstract

**Background:**

The first case of Severe Acute Respiratory Syndrome Coronavirus 2 (SARS-CoV-2) infection in Rio Grande do Norte, northeastern Brazil, was diagnosed on March 12, 2020; thereafter, multiple surges of infection occurred, similar to what was seen elsewhere. These surges were mostly due to SARS-CoV-2 mutations leading to emergence of variants of concern (VoC). The introduction of new VoCs in a population previously exposed to SARS-CoV-2 or after vaccination has been a challenge to understanding the kinetics of the protective immune response against this virus. The aim of this study was to investigate the outbreak of SARS-CoV-2 reinfections observed in mid-January 2022 in Rio Grande do Norte state, Brazil. It describes the clinical and genomic characteristics of nine cases of reinfection that occurred coincident with the introduction of the omicron variant.

**Methodology/Principal findings:**

Of a total of 172,965 individuals with upper respiratory symptoms tested for SARS-CoV-2, between March 2020 through mid-February 2022, 58,097 tested positive. Of those, 444 had documented a second SARS-CoV-2 infection and nine reinfection cases were selected for sequencing. Genomic analysis revealed that virus lineages diverged between primary infections and the reinfections, with the latter caused by the Omicron (BA.1) variant among individuals fully vaccinated against SARS-CoV-2.

**Conclusions/Significance:**

Our findings suggest that the Omicron variant is able to evade both natural and vaccine-induced immunity, since all nine cases had prior natural infection and, in addition, were fully vaccinated, emphasizing the need to develop effective blocking vaccines.

## Author summary

The pattern of the COVID-19 pandemic has been characterized by multiple surges of infection with a variety of outcomes. By December 2021, about 75.3% of the population of the state of Rio Grande do Norte in northeastern Brazil, had been fully vaccinated against SARS-CoV-2 and a decrease in case detection from a mean of 32% of positives to about 8% of cases tested. Nevertheless, with the introduction of the Omicron variant at the end of 2021, the number of new SARS-CoV-2 infections reached its highest peak, with 75% of the suspected cases testing positive. Among the positive cases diagnosed in the Institute of Tropical Medicine between March 2020 to mid-February 2022 a total of 444 reinfections were identified; of which 62.3% (n = 277) occurred during the Omicron outbreak. Of the reinfection cases, nine were sequenced as part of genomic surveillance. Analysis showed that the reinfections’ lineages belonged to BA.1 introduced multiple times into the region; whereas, the isolates from the first infection belonged to different lineages. Thus, our data suggest that the Omicron variant evades immunity from natural infection from any other SARS-CoV-2 variants as well as from the available vaccines, indicating the need for development of effective blocking vaccines.

## Introduction

The world has followed closely the spread of SARS-CoV-2 since 2019 in real time. However, after two years since the first cases of Coronavirus Disease 2019 (COVID-19) were reported in China, much is still unknown about the mutations and the molecular evolution of SARS-CoV-2 [1,2]. The emerging new SARS-CoV-2 lineages may lead to a field of concerns since human immune responses to natural infection or to vaccination vary; although people tend to manifest lower morbidity and mortality, they still are able to shed virus and contribute to virus circulation in a community [3,4].

SARS-CoV-2 variants with potentially increased transmissibility, virulence, and resistance to antibody neutralization have already been reported in several locations [5,6]. The dissemination of variants of concern (VoC) are also closely associated with higher viral load [7], and/or rates of reinfections [8]. Some VoCs can evade the protective effects of humoral immunity induced either by natural infection or by vaccination [9,10], posing an additional challenge to curb the pandemic, since continuous transmission and an increased number of reinfections are being reported worldwide in 2022 [11].

The multi-surge COVID19 pandemic dynamics fit different scenarios depending on the geographic location of emergence of the SARS-CoV-2 variants and the time when the variant was introduced and the previous burden of other variants. Some variants can have a significant impact in specific parts of the world. For example, studies have shown that the Gamma (P.1) variant was more prevalent in South America [12–14]. In contrast, the Delta variant and its sublineages [8,15,16] were found all over the world, apparently with less pressure in Brazil, but Delta was introduced in Brazil right after the worse surge of Gamma and potentially could the reason why the burden and mortality here was not as high as seen elsewhere [15].

Recently, Brazil faced the third wave of COVID-19 pandemic with a record upsurge of cases that started at the end of December 2021 and had been sustained by the highly transmissible variant B.1.1.529, a VoC, named Omicron [17]. In Brazil, COVID-19 vaccination started on January 17, 2021. However, despite the high immunization coverage, with over 79% of the population receiving at least one dose of the vaccine [18], there was sustained number of cases throughout 2021, and some COVID-19 reinfection cases were reported among fully vaccinated people [10].

From March 2020 to mid-February 2022, 444 reinfection episodes were detected in our clinic, of which 277 cases were diagnosed in the first two months of 2022. Thus, we hypothesized that the reinfection cases detected were caused by the recently introduced Omicron strain. Here, we describe nine reinfection cases with the Omicron variant in January 2022 among people who had been fully vaccinated. We provide genomic variation data and clinical features between primary infection and reinfection cases.

## Materials and methods

### Ethics statement

The study protocol was reviewed and approved by the Federal University of Rio Grande do Norte Ethical committee (36287120.2.0000.5537) and participants gave formal consent to be included into the study.

### Diagnosis of SARS-CoV-2

The Institute of Tropical Medicine of Rio Grande do Norte at the Federal University of Rio Grande do Norte (IMT-UFRN), located in Natal, Brazil, provided service throughout the COVID-19 pandemic for diagnosis of SARS-CoV-2 infection for 17 municipalities in the State of Rio Grande do Norte and conducted an outpatient clinic for COVID-19. Clinical data and nasopharyngeal swab specimens were collected at the municipalities or at the IMT-UFRN. All clinical data were stored in the IMT-UFRN database. Nasopharyngeal swabs collected and placed in Viral Transport Medium. Samples were tested for SARS-CoV-2, within 24 hours after collection, by reverse transcriptase quantitative polymerase chain reaction (RT-qPCR), using CDC/EUA protocol [19]. Remnant samples were stored in the IMT- UFRN Biorepository at −80°C and a formal consent was provided by the volunteers to allow their use in this study.

We conducted a descriptive retrospective survey of a large cohort to assess reinfection among the cases tested at IMT-UFRN between June 2020 and February, 2022, in which of 172,965 people tested for SARS-CoV-2 a total of 58,097 were positive. Of those positive, 444 had a second SARS-CoV-2 infection. However, we selected isolates for sequencing from nine reinfection cases (two isolates per case, 18 total sequenced) who presented at the outpatient clinic and whose follow-up data were known. The 18 samples had a cycle threshold (Ct) value ≤ 30. This work was part of a larger genomic surveillance effort performed by the Institute of Tropical Medicine of Rio Grande do Norte at the Federal University of Rio Grande do Norte to follow for potential entrance of newly SARS-CoV-2 variants.

### RNA extraction, library preparation and sequencing

Viral RNA was isolated from 200-μl samples collected from people suspected of having SARS-CoV-2 infections using the QIAamp Viral RNA Mini kit (QIAGEN, Hilden, Germany) according to the manufacturer’s instructions. Virus genome sequencing was carried out on all positive samples selected using a COVIDSeq protocol (Illumina Inc, USA).

cDNA synthesis was performed from the extracted RNA using a multiplex polymerase chain reaction (PCR) protocol, producing 98 amplicons across the SARS-CoV-2 genome (https://artic.network/). The primer pool additionally had primers targeting human RNA, producing an additional 11 amplicons. The PCR-amplified product was later processed for tagmentation and adapter ligation using IDT for Illumina Indexes. Further enrichment and cleanup were performed as per protocols provided by the manufacturer (Illumina Inc). All samples were processed as batches in 16-well plates. Pooled samples were quantified using Qubit 2.0 fluorometer (Invitrogen Inc.) and fragment sizes were analyzed in 4150 TapeStation System (Agilent Inc). The pooled library was further normalized to 4nM concentration and 25 μl of each normalized pool containing index adapter set A were combined in a new microcentrifuge tube to a final concentration of 100pM. The pooled libraries were loaded and clustered onto iSeq 100 i1 Reagent v2 (Illumina) with a flow cell and sequenced using synthesis (SBS) chemistry on the Illumina iSeq 100 sequencing system. The sequencing run was created with Local Run Manager software installed on the iSeq 100 instrument. Using the Generate FASTQ Analysis module with the custom library preparation kit options, the number of sequencing cycles was set to 151 bp and the paired-end sequencing option was determined.

### Read mapping and consensus calling

All reads were mapped to the reference SARS-CoV-2 genome (GenBank accession number MN908947.3) using the BWA-MEM V0.7.12-r1039 [17]. Next, the removal of duplicate reads and the split of reads containing poorly sequenced base was proceeded by the Genomic Analysis-Toolkit (GATK) V4.2.0.0 [18, 17]; the quality of the final mapping process was accessed by Mosdepth [19] and Qualimap V2.2.1 [20]. The SNV identification was performed by the *HaplotypeCaller* function from the GATK, followed by the filtration using the *VariantFiltration*, *FilterVcf* and *SelectVariants*, excluding variants with depth lower than 10. Those variants which passed by the depth threshold were annotated using the SnpEff V5.0e [21].

Sequence consensus calling was performed by the *FastaAlternateReferenceMaker* function from GATK, and for the final fasta file those positions covered by fewer than 10 reads were replaced by Ns, by using an in-house script written in Python 3.8 (available at GitHub). All sequences were submitted to the Pangolin COVID-19 Lineage Assigner v.3.1.17 [20] (accessed on Jan 24, 2022). The whole genome sequences of viral variants of the nine samples (two samples from each case of reinfection) were deposited in the GISAID database (accession nos. EPI_ISL_9467461, EPI_ISL_9467460, EPI_ISL_9467471, EPI_ISL_9467463, EPI_ISL_9467462, EPI_ISL_9467470, EPI_ISL_9467458, EPI_ISL_9467469, EPI_ISL_9467457, EPI_ISL_9467468, EPI_ISL_9467459, EPI_ISL_9467454, EPI_ISL_9467465, EPI_ISL_9467464, EPI_ISL_9467456, EPI_ISL_9467467, EPI_ISL_9467455, EPI_ISL_9467466) and all GISAID sequences used in this study are listed in S3 Table.

### Phylogenetics analysis

The 18 genome sequences obtained in this work were aligned to 700 other genome sequences from Rio Grande do Norte, Brazil, obtained from GISAID on January 20, 2022, using MAFFT v.7.394 [21]. Then, we constructed a maximum likelihood (ML) phylogenetics tree supported by 1,000 replicates of bootstrap using IQ-TREE2 [22], under the substitution model GTR+F+R4, which was inferred to be the best-fitting model by de software itself. The same procedure was performed for the alignment containing only the Brazilian BA.1 genomes, under the GTR+F+R3 model. Tree topologies were inspected by Figtree v.1.4.4 (http://tree.bio.ed.ac.uk/software/figtree/ last accessed 2021-11-29).

### Air travels to the state of Rio Grande do Norte

Since there were multiple introductions of Omicron into the state of Rio Grande do Norte within a few days after the variant was introduced in Brazil, we hypothesized that more likely cities that had more flights to Rio Grande do Norte could be implicated in the type of lineage introduced. Therefore, we collected data on the total number of flights landing in Rio Grande do Norte, from December 1, 2021 to December 31, 2021, as registered at the National Civil Aviation Agency (ANAC), by accessing the page Consultations of past flights (https://sas.anac.gov.br/, accessed on January 24, 2022). Data from January-1-2022 to January-20-2022 was obtained from ANAC by means of consultation of planned flights (https://siros.anac.gov.br, January 24, 2022).

## Results

### Reinfection cases in Rio Grande do Norte, Brazil

**Fig 1:**
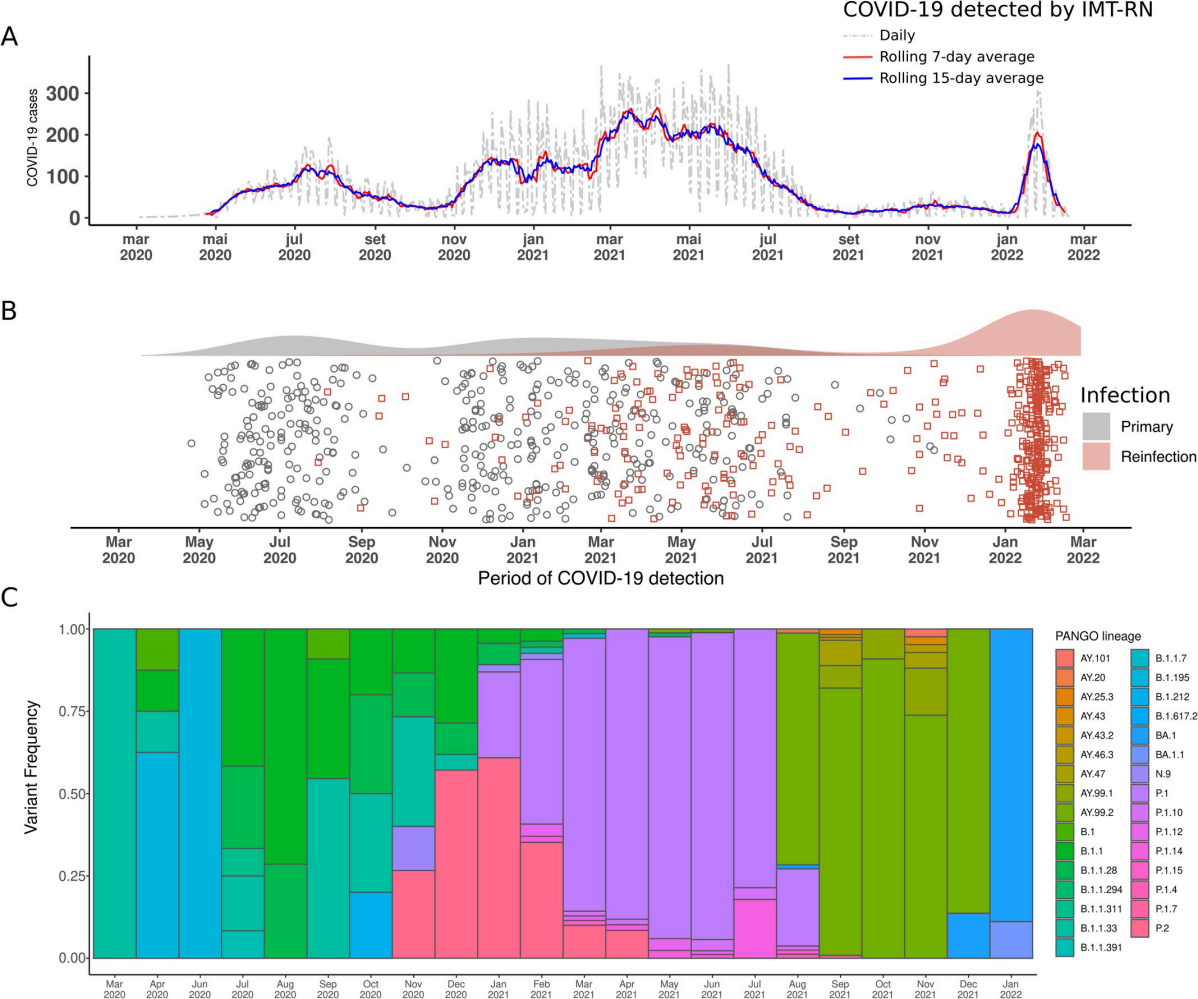
Timeline of COVID-19 detection cases in Rio Grande do Norte state, Brazil. (A) Positive COVID-19 cases in RN detected by RT-qPCR in IMT-RN laboratory. (B) Detection of SARS-CoV-2 reinfection in 444 individuals. Participants with two entries were identified and the first (gray circle) and second (red square) entries were plotted. A gray line connects both COVID-19 tests for the same individual. The density of COVID-19 cases on top of the plot. (C) SARS-CoV-2 PANGO lineages sequenced in the state from March 2020 to January 2022, obtained from GISAID.

From March 2020 to February 2022, a total of 172,965 individuals with upper respiratory symptoms underwent testing by RT-qPCR for SARS-CoV-2 at IMT-RN, of which 58,097 tested positive. Positive tests remained high from December 2020 to July 2021 (mean 160.9/day, 100.4 SD), when thereafter a decrease of COVID-19 cases was observed until the beginning of January 2022 (mean 27.8/day, 28.5 SD), followed by a rapid increase in positivity (Fig 1A). Among those, 444 individuals had two episodes of symptomatic infection for COVID-19 (Fig 1B). Reinfection cases had already been reported in mid-2020, although they seemed to be scarce. Reinfections, however, reached a peak at 62.3% (n = 277) of all reinfection cases identified in our system during the Omicron-predominant period in early 2022, when sequences in the state of Rio Grande do Norte corresponded to 100% of Omicron lineages in January (Fig 1C). The dates of sample collections and vaccination data are shown in the S1 Table. Although one of the cases had reinfection under 90 days, the sequence data indicated that the infections were due to two different lineages (S2 Table). None of the subjects had any comorbidities such as hypertension, diabetes mellitus, HIV, cancer, or asthma/COPD.

### Demographic data, clinical characteristics, and vaccination status

**Fig 2:**
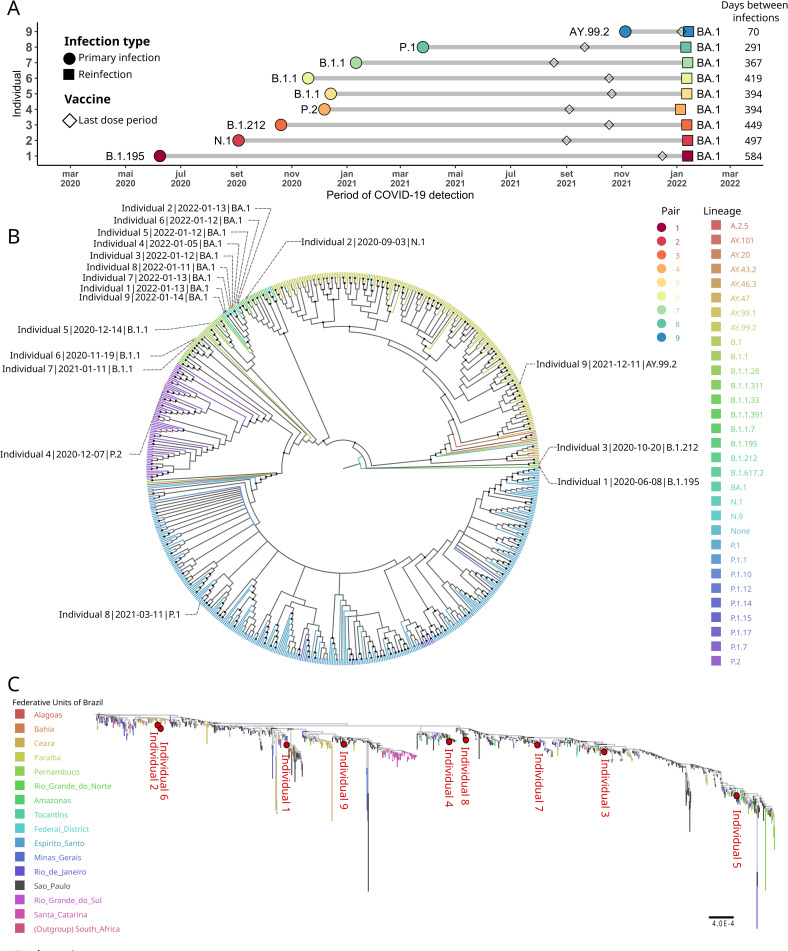
Identification of SARS-CoV-2 reinfection cases in Rio Grande do Norte, Brazil. (**A**) Time course of primary infection and reinfection and different virus lineages. The circles and squares indicate the primary infection and reinfection, respectively. The polygonal rhomb shows the time of the last COVID-19 vaccine shot. The x-axis indicates the time period in months. The y-axis denotes a random ID number assigned to each individual. (**B**) Maximum-likelihood tree of all 709 SARS-CoV-2 whole-genome sequences available at GISAID repository and downloaded on January 20, 2022. The colors assigned to the branches are related to their respective lineages and the sequenced samples in this work are labeled according to their paired, sampling dates, and virus lineages, respectively. The dark dots at the dichotomies represent those nodes with high bootstrap support (≥ 80). The tree’s root was set on the youngest isolate (from June 8, 2020), sequenced in this work, which belongs to the B.1.195 lineage. (**C**) ML tree composed of 1700 other Omicron sequences isolated in Brazil plus a B.1.1.529 reference as an outgroup (EPI_ISL_6704867), with the branches colored according to the Brazilian state where the virus was isolated. The red dots indicate where each of the nine sequences is placed in the tree. Each of the nine isolates and the other sequences from Brazil close to them can be seen in S2 Fig.

Of the 444 individuals with documented SARS-CoV-2 reinfection, nine immunocompetent and fully vaccinated individuals were chosen to have their isolates sequenced (Figs 2A and S1). Among those, five were males and the mean age of the subjects was 32.4 years (± 7.4). The first infections ranged from June 2020 to November 2021. All reinfection cases occurred in January 2022. The time course between the two episodes of COVID-19 ranged between 70 and 584 days, with mild clinical presentation for primary infection and reinfection (Fig 2A). The most reported symptoms during the primary infection and reinfection were headache, nasal congestion, fatigue, rhinorrhea, myalgia, pharyngitis, fever, and cough (S3 Table). None of the subjects were hospitalized during the two episodes of COVID-19. In addition, all subjects were fully vaccinated and three had already received a booster.

### SARS-CoV-2 isolates sequencing

In order to distinguish between reinfection and residual RNA from a previous infection, so-called long-term viral persistence, we performed a maximum-likelihood (ML) phylogenetic analysis of all SARS-CoV-2 whole genomes from Rio Grande do Norte state, recovered from GISAID on January 20, 2021, and combined with two pairs of isolates taken from each individual during SARS-CoV-2 primary infection and reinfection (Fig 2B). The earliest isolate reported from Rio Grande do Norte state was obtained on July 02, 2020, and belongs to the BA.1.1.311 lineage (EPI_ISL_2466179), but herein we sequenced the oldest one, from June 08, 2020, which was assigned to the B.1.195 lineage by the Pangolin tool when the ML tree root was set. The tree topology revealed that all sequences belonged to the variant of concern Omicron (BA.1) lineage and they were clustered in a monophyletic clade while their respective pairs, from the previous SARS-CoV-2 infection, were found spread throughout the tree. A sequence from individual # 2, isolated during the first infection and assigned to the N.1 lineage, was placed close to the Omicron branch. The N.1 lineage had been detected in Brazil during the early epidemic in 2020, according to the *outbreak*.*info* website.

Among the nine individuals who had reinfection, the elapsed time between infections was from 584 to 70 days (Fig 2A). Interestingly, a female individual had a reinfection 70 days apart from primary infection and 8 days after the COVID-19 vaccine booster shot, in which, despite this short interval between overt disease, the virus lineages diverged between the two infections (Fig 2A and 2B); the primary was an AY.99.2 variant, while the reinfection was by BA.1. It is important to note the high frequency of AY lineages (Delta VOC) on the topology ML tree, representing more than 30% of all sequences in Rio Grande do Norte state, and also that variants arising from Delta were found on almost 100% of the sequences isolated in Brazil in December 2021 [23].

To better characterize the Omicron variant detected during the reinfection cases, we aligned it against all other Brazilian BA.1 whole genomes available in the GISAID database until January 20, 2022. In addition, we also selected an African B.1.1.529 reference sequence as an outgroup (accession EPI_ISL_6704867). The second ML tree (Fig 2C) revealed that on a broader scale of this single lineage, the genomes isolated from the reinfection are spread through the tree, emphasizing the multiple introductions of the BA.1 lineage into the Rio Grande do Norte state. S2 Fig shows the relationship of the 9 BA.1 isolates sequenced here, with a notable presence of isolates from the states of São Paulo, Pernambuco, and Ceará. From early December, 2021 to mid-January, 2021, São Paulo and Pernambuco were, respectively, the first and second Brazilian states with the highest number of flights departing to Rio Grande do Norte (S3 Fig).

## Discussion

SARS-CoV-2 infection episodes by emerging variants in previously naturally infected or vaccinated individuals are already a real phenomenon worldwide, which poses an important issue for understanding human immune responses to this virus and its response to vaccines [11]. Reinfection cases have been rising profoundly since the Omicron variant of SARS-CoV-2 started to disseminate in November 2021 [24]. To date, such a fact has not been observed at the same rate in previously reported variants of concern of SARS-CoV-2. In our study, we demonstrate that 62.3% (277/444) of all reinfection cases detected over 22 months occurred during the beginning of the Omicron-surge (Fig 1). To confirm those reinfection events, we investigated a small cohort study of nine individuals with reinfection of COVID-19 diagnosed in our center in Natal, state of Rio Grande do Norte, Brazil. This state receives a high number of tourists year-round, and this may have been one of the reasons for the diversity of introductions in the region. From early-December 2021 to mid-January 2022, São Paulo was the Brazilian state responsible for 44.9% of flights that landed in Natal (S3 Fig), which may explain the similarities between the majority of the isolates sequenced herein and the ones sequenced in São Paulo. This, together with the fact that São Paulo is one of the states most affected by the omicron variant [25], due to being a hub for intercontinental flights, means that it has an increased risk of new variants, as demonstrated by the Omicron case, as well as the means of dissemination throughout the country.

The Brazilian Health Ministry criteria for a SARS-CoV-2 reinfection is two positive RT-qPCR confirmations at least 90 days apart, with clinical manifestations characteristics of COVID-19, positive viral culture, and with additional viral RNA sequencing from both episodes showing different strains [10,26]. Our work lacks the viral culture. Nevertheless, we demonstrated one case of an individual with two infection episodes, 70 days apart, and the sequences of the two isolates showed clearly different lineages. Reinfection cases less than 90 days apart have already been described [27].

Recent studies have demonstrated that Omicron-infected cases tend to cause mild symptoms such as pharyngitis, headache, and nasal congestion [28], which corroborates our data. Despite three subjects each receiving a booster shot, either with the third dose (BNT162b2) or an additional dose (Ad26.COV2.S) (S1 Fig), each developed symptomatic COVID-19, as did the other individuals who had complete vaccination, suggesting that even with a booster shot, these subjects were still susceptible and they did not present an effective immune response capable of preventing a new SARS-COV-2 infection, similarly as reported previously by Andrews and colleagues [29]. Beyond that, it is out of notice that those individuals were immunocompetent, they had no comorbidity, were not taking any medication, and were not aware of any other ongoing infection that could compromise their immune system and had been otherwise previously healthy.

Our data support the findings that neither the current vaccines available nor previous history of COVID-19 is sufficient to establish a robust response against the Omicron variant and to promote a reduced risk of infection and overt disease. Although we did not perform any neutralizing antibody assays on the nine participants in this study, others have found that the ability to neutralize Omicron was lower in those who were vaccinated and had previously been infected, or in those who were only vaccinated, compared to prior lineages [29–31], although a recent study showed that booster vaccination increases the neutralization titers to Omicron [9].

We have only begun to explore the many ways that the Omicron lineage or newer variants are capable of causing reinfections and continuous genomic surveillance is needed to understand the role of SARS-CoV-2 mutations, which could lead to immune response evasion generated by prior infections due to other variants or to the available vaccination. A recent study has hypothesized that some mutations in the spike RBD domain, present on Omicron, are enough to anchor the virus on the ACE2 receptor, leaving the rest of the receptor binding motif free to develop further mutations, which would facilitate the evasion of the neutralizing antibodies [32]. Although the currently available vaccines reduced the severity of COVID-19, an increase in mortality was observed in Brazil in January, at the time of Omicron surge when compared to the Delta variant surge [33]. This underscores the need to develop more effective vaccines with the potential for reducing transmission and a lengthier protective immune response. Genomic surveillance is key for assessing new variants and therefore suitable for planning new strategies to battle COVID-19.

Our study does have some limitations. The number of RT-PCR tests performed on samples collected from people with respiratory symptoms decreased as a result of the increased use of COVID-19 rapid antigen testing and the outbreak of influenza, concomitant to the introduction of Omicron. In addition, people who had mild symptoms tended not to seek testing or care, or did at home COVID test and not reporting the results, therefore limiting the detection of more reinfection cases and true estimate of the surge. We sequenced only 18 isolate samples (two per subject) since we aimed to carry on surveillance of new variants, not only reinfection cases. Aside from that, those who received the same vaccine did not have comparable dose intervals to assess long term immunity and we have a limited number of subjects who received the second–third dose of vaccine. In regards to the participant’s immunocompetence, we were unable to determine whether they were capable of producing antibodies in response to vaccinations and/or natural infections, which could be properly assessed using a neutralization assay [34], considering that immunocompromised individuals have a lower rate of seroconversion when exposed to SARS-CoV-2 particles [35,36]. Our aim was to demonstrate the Omicron variant’s ability to cause reinfections, and not to diminish the role of naturally acquired and/or vaccinated immunity, which has already been established by preventing COVID-19 severity and reducing hospitalizations and lethality [37]. SARS-CoV-2 should be regarded as a virus that is still in the process of constant changes and as result, the disease it causes, COVID-19, is not a single event.

In summary, our data suggest that the Omicron variant evades immunity provided by different types of vaccines or from natural infection by any other earlier SARS-CoV-2 variants of concern and may be related mostly to the mild symptoms of the upper respiratory tract for the majority of the subjects. Thus, along with vaccination, the reassurance of protective measures needs to be held to prevent further spread of SARS-CoV-2 variants or the risk of the appearance of new variants.

## Supporting information

S1 Table Sample collection and vaccination dates. (XLSX)

S2 Table COVD-19 signs and symptoms in the first infection and reinfection in the nine individuals included in this study. (PDF)

S3 Table GISAID sequences used in this study. (XLSX)

S1 Fig Schematic representation of different types of vaccine against SARS-CoV-2 in all reinfection cases. (PDF)

S2 Fig Subset of the Omicron (BA.1) ML phylogenetic tree indicating the closest isolates from different areas of Brazil. The Omicron genomes sequenced here are indicated with the red tips.
(PDF)

S3 Fig Cumulative sum of flights according to the Brazilian federative unit or Country of origin. The order from the Brazilian federative unit with the most flights to Natal (from December 01, 2021 to January 20, 2022) from, to the lesser one, is: São Paulo (604 flights); Pernambuco (227 flights); Brasília (199 flights); Minas Gerais (85 flights); Rio de Janeiro (78 flights); Bahia (53 flights); Ceará (52 flights); Rio Grande do Norte (20 flights); Paraná (4 flights); Goiás (1 flight); and also Portugal with 22 international flights.
(PDF)
