# Supplementary material for: SARS-CoV-2 reinfections with BA.1 (Omicron) variant among fully vaccinated individuals in northeastern Brazil
Source: PLoS Negl Trop Dis. 2022 Oct 3;16(10):e0010337. doi: 10.1371/journal.pntd.0010337 (PMC9560550; doi:10.1371/journal.pntd.0010337)
Supplement: S2 Table — (PDF) [file pntd.0010337.s002.pdf]

We gratefully acknowledge the following Authors from the Originating laboratories responsible for obtaining the specimens, as well as the Submitting laboratories where the genome data were generated and shared via GISAID, on which this research is based.

All Submitters of data may be contacted directly via www.gisaid.org

Authors are sorted alphabetically.

Accession ID	Originating Laboratory	Submitting Laboratory	Authors
EPI_ISL_7883749, EPI_ISL_7883750, EPI_ISL_8184812, EPI_ISL_8184813, EPI_ISL_8184814, EPI_ISL_8184815, EPI_ISL_8184816, EPI_ISL_8184817, EPI_ISL_8184818, EPI_ISL_8184819, EPI_ISL_8184820, EPI_ISL_8184821, EPI_ISL_8184822, EPI_ISL_8184823, EPI_ISL_8184824, EPI_ISL_8184825, EPI_ISL_8184826, EPI_ISL_8184827, EPI_ISL_8184828, EPI_ISL_8721233, EPI_ISL_8721247, EPI_ISL_8721297, EPI_ISL_8721302, EPI_ISL_8721377, EPI_ISL_8721418, EPI_ISL_8721432, EPI_ISL_8721433, EPI_ISL_8721457, EPI_ISL_8721464, EPI_ISL_8721526, EPI_ISL_8721527, EPI_ISL_8721568, EPI_ISL_8721589, EPI_ISL_8721627, EPI_ISL_8721699, EPI_ISL_8721700, EPI_ISL_8721723, EPI_ISL_8721724, EPI_ISL_8721725, EPI_ISL_8721726, EPI_ISL_8721751, EPI_ISL_8721770, EPI_ISL_8721842, EPI_ISL_8721899, EPI_ISL_8721923, EPI_ISL_8721943, EPI_ISL_8721944, EPI_ISL_8721964, EPI_ISL_8721996			
see above	AFIP	Instituto Butantan	Antonio Jorge Martins; Claudia Renata dos Santos Barros; David Schlesinger; Debora Botequio Moretti; Dimas Tadeu Covas; Elaine Cristina Marqueze; Elaine Vieira Santos; Evandra Strazza Rodrigues; Heidge Fukumasu; Jayme Augusto de Souza-Neto; José Salvatore Leister Patané; Luiz Alcantara; Luiz Lehmann Coutinho; Maria Carolina Elias; Maurício Lacerda Nogueira; Rafael dos Santos Bezerra; Raul Machado Neto; Rejane Maria Tommasini Grotto; Ricardo Haddad; Sandra Coccuzzo Sampaio Vessoni; Simone Kashima; Svetoslav Nanev Slavov; Vincent Louis Viala
EPI_ISL_5915117, EPI_ISL_5915120, EPI_ISL_5915123, EPI_ISL_5915124, EPI_ISL_5915125, EPI_ISL_5915127, EPI_ISL_5915128, EPI_ISL_5915129, EPI_ISL_5915131, EPI_ISL_5915132, EPI_ISL_5915133, EPI_ISL_5915134, EPI_ISL_5915135, EPI_ISL_5915138, EPI_ISL_5915140, EPI_ISL_5915142, EPI_ISL_5915143, EPI_ISL_5915144, EPI_ISL_5915146, EPI_ISL_5915147, EPI_ISL_5915150, EPI_ISL_5915151, EPI_ISL_5915152, EPI_ISL_5915157, EPI_ISL_5915159, EPI_ISL_5915161, EPI_ISL_5915162, EPI_ISL_5915163, EPI_ISL_5915169, EPI_ISL_5915171, EPI_ISL_5915172, EPI_ISL_5915173, EPI_ISL_5915174, EPI_ISL_5915177, EPI_ISL_5915180, EPI_ISL_5915181, EPI_ISL_5915183, EPI_ISL_5915186, EPI_ISL_5915187, EPI_ISL_5915188, EPI_ISL_5915189, EPI_ISL_5915190, EPI_ISL_5915191, EPI_ISL_5915192, EPI_ISL_5915193, EPI_ISL_5915194, EPI_ISL_5915195, EPI_ISL_5915196, EPI_ISL_5915197, EPI_ISL_5915201, EPI_ISL_5915202, EPI_ISL_5915203, EPI_ISL_5915204, EPI_ISL_5915205, EPI_ISL_5915206, EPI_ISL_5915207, EPI_ISL_5915208, EPI_ISL_5915209, EPI_ISL_5915210, EPI_ISL_5915211, EPI_ISL_5915212, EPI_ISL_5915213, EPI_ISL_5915214, EPI_ISL_5915215, EPI_ISL_5915216, EPI_ISL_5915217, EPI_ISL_5915218, EPI_ISL_5915219, EPI_ISL_5915220, EPI_ISL_5915221, EPI_ISL_5915222, EPI_ISL_5915223, EPI_ISL_5915224, EPI_ISL_5915225, EPI_ISL_5915226, EPI_ISL_5915227, EPI_ISL_5915228, EPI_ISL_5915229, EPI_ISL_5915230, EPI_ISL_5915231, EPI_ISL_5915232, EPI_ISL_5915233, EPI_ISL_5915234, EPI_ISL_5915235, EPI_ISL_5915236, EPI_ISL_5915237, EPI_ISL_5915238, EPI_ISL_5915239, EPI_ISL_5915240, EPI_ISL_5915241, EPI_ISL_5915242, EPI_ISL_5915243, EPI_ISL_5915244, EPI_ISL_5915245, EPI_ISL_5915246, EPI_ISL_5915247, EPI_ISL_5915248, EPI_ISL_5915249, EPI_ISL_5915250, EPI_ISL_5915251, EPI_ISL_5915252, EPI_ISL_5915253, EPI_ISL_5915254, EPI_ISL_5915255, EPI_ISL_5915256, EPI_ISL_5915257, EPI_ISL_5915258, EPI_ISL_5915259, EPI_ISL_5915260, EPI_ISL_5915261, EPI_ISL_5915262, EPI_ISL_5915263, EPI_ISL_5915264, EPI_ISL_5915265, EPI_ISL_5915266, EPI_ISL_5915267, EPI_ISL_5915268, EPI_ISL_5915269, EPI_ISL_5915270, EPI_ISL_5915271, EPI_ISL_5915272, EPI_ISL_5915273, EPI_ISL_5915274, EPI_ISL_5915275, EPI_ISL_5915276, EPI_ISL_5915277, EPI_ISL_5915278, EPI_ISL_5915279, EPI_ISL_5915282, EPI_ISL_5915283, EPI_ISL_5915284, EPI_ISL_5915285, EPI_ISL_5915286, EPI_ISL_5915287, EPI_ISL_5915288, EPI_ISL_5915289, EPI_ISL_5915290, EPI_ISL_5915291, EPI_ISL_5915292, EPI_ISL_5915293, EPI_ISL_5915294, EPI_ISL_5915295, EPI_ISL_5915296, EPI_ISL_5915297, EPI_ISL_5915298, EPI_ISL_5915299, EPI_ISL_5915300, EPI_ISL_5915301, EPI_ISL_5915302, EPI_ISL_5915303, EPI_ISL_5915304, EPI_ISL_5915305, EPI_ISL_5915306, EPI_ISL_5915307, EPI_ISL_5915308, EPI_ISL_5915309, EPI_ISL_5915310, EPI_ISL_5915311, EPI_ISL_5915312, EPI_ISL_5915313, EPI_ISL_5915314, EPI_ISL_5915315, EPI_ISL_5915316, EPI_ISL_5915317, EPI_ISL_5915318, EPI_ISL_5915319, EPI_ISL_5915323, EPI_ISL_5915325, EPI_ISL_5915326, EPI_ISL_5915327, EPI_ISL_5915328, EPI_ISL_5915377, EPI_ISL_5915378, EPI_ISL_5915379, EPI_ISL_5915380, EPI_ISL_5915381, EPI_ISL_5915382, EPI_ISL_5915383, EPI_ISL_5915384, EPI_ISL_5915385, EPI_ISL_5915386, EPI_ISL_5915387, EPI_ISL_6573783, EPI_ISL_6573786, EPI_ISL_6573787, EPI_ISL_6573788, EPI_ISL_6573789, EPI_ISL_6573790, EPI_ISL_6573791, EPI_ISL_6573792, EPI_ISL_6573793, EPI_ISL_6573797, EPI_ISL_6573799, EPI_ISL_6573806, EPI_ISL_6573807, EPI_ISL_6573809, EPI_ISL_6573810, EPI_ISL_6573815, EPI_ISL_6573817, EPI_ISL_6573818, EPI_ISL_6573819, EPI_ISL_6573820, EPI_ISL_6573821, EPI_ISL_6573822, EPI_ISL_6573823, EPI_ISL_6573824, EPI_ISL_6573825, EPI_ISL_6573826, EPI_ISL_6573827, EPI_ISL_6573828, EPI_ISL_6573829, EPI_ISL_6573830, EPI_ISL_6573833, EPI_ISL_6573834, EPI_ISL_6573836, EPI_ISL_6573837, EPI_ISL_6573838, EPI_ISL_6573841, EPI_ISL_6573843, EPI_ISL_6573852, EPI_ISL_6573853, EPI_ISL_6573854, EPI_ISL_6573855, EPI_ISL_6573856, EPI_ISL_6573857, EPI_ISL_6573858, EPI_ISL_6573859, EPI_ISL_6573861, EPI_ISL_6573862, EPI_ISL_6573863, EPI_ISL_6573865, EPI_ISL_6573866, EPI_ISL_6573867, EPI_ISL_6573868, EPI_ISL_6573869, EPI_ISL_6573872, EPI_ISL_6573873, EPI_ISL_6573874, EPI_ISL_6573875, EPI_ISL_6573876, EPI_ISL_6573878, EPI_ISL_6573879, EPI_ISL_6573880, EPI_ISL_6573915, EPI_ISL_6573919, EPI_ISL_6573924, EPI_ISL_6573929, EPI_ISL_6573930, EPI_ISL_6573938, EPI_ISL_6573945, EPI_ISL_6573946, EPI_ISL_6573948, EPI_ISL_6573951, EPI_ISL_6573952, EPI_ISL_6573955, EPI_ISL_6573956, EPI_ISL_6573957, EPI_ISL_6573960, EPI_ISL_6573961, EPI_ISL_6573962			
see above	AMALAB/FACISA/UFRN	WallauLab on behalf of Fiocruz COVID-19 Genomic Surveillance Network	Alexandre Freitas da Silva; Allan Roberto Dias Nunes; Antonio Marinho da Silva Neto; Cassia Docena; Constância Flávia Junqueira Ayres; Filipe Zimmer Dezordi; Gabriel Luz Wallau; Gustavo Barbosa de Lima; Joana Cristina Medeiros Tavares Marques; Katya Anaya Jacinto; Lais Ceschini Machado; Lilian Caroliny Amorim Silva; Marcelo Henrique dos Santos Paiva; Mariane dos Santos Duarte; Matheus Filgueira Bezerra; Sinval Pinto Brandão Filho
EPI_ISL_7779341, EPI_ISL_7779342, EPI_ISL_8184805, EPI_ISL_8184806, EPI_ISL_8184807, EPI_ISL_8184808, EPI_ISL_8184809, EPI_ISL_8184810, EPI_ISL_8184811, EPI_ISL_8721211, EPI_ISL_8721213, EPI_ISL_8721214, EPI_ISL_8721215, EPI_ISL_8721222, EPI_ISL_8721223, EPI_ISL_8721230, EPI_ISL_8721234, EPI_ISL_8721238, EPI_ISL_8721239, EPI_ISL_8721240, EPI_ISL_8721241, EPI_ISL_8721245, EPI_ISL_8721257, EPI_ISL_8721258, EPI_ISL_8721259, EPI_ISL_8721267, EPI_ISL_8721277, EPI_ISL_8721283, EPI_ISL_8721287, EPI_ISL_8721292, EPI_ISL_8721293, EPI_ISL_8721294, EPI_ISL_8721295, EPI_ISL_8721303, EPI_ISL_8721308, EPI_ISL_8721309, EPI_ISL_8721310, EPI_ISL_8721311, EPI_ISL_8721312, EPI_ISL_8721323, EPI_ISL_8721326, EPI_ISL_8721327, EPI_ISL_8721328, EPI_ISL_8721329, EPI_ISL_8721330, EPI_ISL_8721342, EPI_ISL_8721343, EPI_ISL_8721344, EPI_ISL_8721355, EPI_ISL_8721365, EPI_ISL_8721366, EPI_ISL_8721367, EPI_ISL_8721368, EPI_ISL_8721378, EPI_ISL_8721387, EPI_ISL_8721388, EPI_ISL_8721389, EPI_ISL_8721402, EPI_ISL_8721403, EPI_ISL_8721405, EPI_ISL_8721419, EPI_ISL_8721420, EPI_ISL_8721421, EPI_ISL_8721434, EPI_ISL_8721435, EPI_ISL_8721446, EPI_ISL_8721447, EPI_ISL_8721448, EPI_ISL_8721458, EPI_ISL_8721459, EPI_ISL_8721460, EPI_ISL_8721465, EPI_ISL_8721466, EPI_ISL_8721467, EPI_ISL_8721479, EPI_ISL_8721480, EPI_ISL_8721481, EPI_ISL_8721493, EPI_ISL_8721494, EPI_ISL_8721495, EPI_ISL_8721496, EPI_ISL_8721497, EPI_ISL_8721514, EPI_ISL_8721528, EPI_ISL_8721529, EPI_ISL_8721532, EPI_ISL_8721541, EPI_ISL_8721542, EPI_ISL_8721543, EPI_ISL_8721544, EPI_ISL_8721545, EPI_ISL_8721556, EPI_ISL_8721569, EPI_ISL_8721570, EPI_ISL_8721571, EPI_ISL_8721572, EPI_ISL_8721573, EPI_ISL_8721590, EPI_ISL_8721591, EPI_ISL_8721592, EPI_ISL_8721593, EPI_ISL_8721594, EPI_ISL_8721610, EPI_ISL_8721611, EPI_ISL_8721612, EPI_ISL_8721613, EPI_ISL_8721628, EPI_ISL_8721629, EPI_ISL_8721630, EPI_ISL_8721638, EPI_ISL_8721639, EPI_ISL_8721640, EPI_ISL_8721641, EPI_ISL_8721642, EPI_ISL_8721643, EPI_ISL_8721644, EPI_ISL_8721645, EPI_ISL_8721660, EPI_ISL_8721661, EPI_ISL_8721662, EPI_ISL_8721663, EPI_ISL_8721665, EPI_ISL_8721681, EPI_ISL_8721682, EPI_ISL_8721683, EPI_ISL_8721701, EPI_ISL_8721702, EPI_ISL_8721703, EPI_ISL_8721704, EPI_ISL_8721705, EPI_ISL_8721727, EPI_ISL_8721728, EPI_ISL_8721729, EPI_ISL_8721730, EPI_ISL_8721752, EPI_ISL_8721753, EPI_ISL_8721754, EPI_ISL_8721771, EPI_ISL_8721772, EPI_ISL_8721775, EPI_ISL_8721776, EPI_ISL_8721798, EPI_ISL_8721799, EPI_ISL_8721800, EPI_ISL_8721801, EPI_ISL_8721802, EPI_ISL_8721803, EPI_ISL_8721804, EPI_ISL_8721805, EPI_ISL_8721822, EPI_ISL_8721823, EPI_ISL_8721824, EPI_ISL_8721825, EPI_ISL_8721843, EPI_ISL_8721844, EPI_ISL_8721845, EPI_ISL_8721846, EPI_ISL_8721847, EPI_ISL_8721848, EPI_ISL_8721849, EPI_ISL_8721850, EPI_ISL_8721851, EPI_ISL_8721871, EPI_ISL_8721872, EPI_ISL_8721873, EPI_ISL_8721874, EPI_ISL_8721875, EPI_ISL_8721900, EPI_ISL_8721901, EPI_ISL_8721902, EPI_ISL_8721924, EPI_ISL_8721925, EPI_ISL_8721926, EPI_ISL_8721927, EPI_ISL_8721945, EPI_ISL_8721965, EPI_ISL_8721980, EPI_ISL_8722002, EPI_ISL_8722003, EPI_ISL_8722007, EPI_ISL_8722008			
see above	BIOFAST	Instituto Butantan	Antonio Jorge Martins; Claudia Renata dos Santos Barros; David Schlesinger; Debora Botequio Moretti; Dimas Tadeu Covas; Elaine Cristina Marqueze; Elaine Vieira Santos; Evandra Strazza Rodrigues; Heidge Fukumasu; Jayme Augusto de Souza-Neto; José Salvatore Leister Patané; Luiz Alcantara; Luiz Lehmann Coutinho; Maria Carolina Elias; Maurício Lacerda Nogueira; Rafael dos Santos Bezerra; Raul Machado Neto; Rejane Maria Tommasini Grotto; Ricardo Haddad; Sandra Coccuzzo Sampaio Vessoni; Simone Kashima; Svetoslav Nanev Slavov; Vincent Louis Viala
EPI_ISL_8721224	CADIP VILA REGINA FERNANDOPOLIS	Instituto Butantan	Antonio Jorge Martins; Claudia Renata dos Santos Barros; David Schlesinger; Debora Botequio Moretti; Dimas Tadeu Covas; Elaine Cristina Marqueze; Elaine Vieira Santos; Evandra Strazza Rodrigues; Heidge Fukumasu; Jayme Augusto de Souza-Neto; José Salvatore Leister Patané; Luiz Alcantara; Luiz Lehmann Coutinho; Maria Carolina Elias; Maurício Lacerda Nogueira; Rafael dos Santos Bezerra; Raul Machado Neto; Rejane Maria Tommasini Grotto; Ricardo Haddad; Sandra Coccuzzo Sampaio Vessoni; Simone Kashima; Svetoslav Nanev Slavov; Vincent Louis Viala
EPI_ISL_8353612, EPI_ISL_8721281	CASA DE SAUDE STELLA MARIS	Instituto Butantan	Antonio Jorge Martins; Claudia Renata dos Santos Barros; David Schlesinger; Debora Botequio Moretti; Dimas Tadeu Covas; Elaine Cristina Marqueze; Elaine Vieira Santos; Evandra Strazza Rodrigues; Heidge Fukumasu; Jayme Augusto de Souza-Neto; José Salvatore Leister Patané; Luiz Alcantara; Luiz Lehmann Coutinho; Maria Carolina Elias; Maurício Lacerda Nogueira; Rafael dos Santos Bezerra; Raul Machado Neto; Rejane Maria Tommasini Grotto; Ricardo Haddad; Sandra Coccuzzo Sampaio Vessoni; Simone Kashima; Svetoslav Nanev Slavov; Vincent Louis Viala
EPI_ISL_8184736	CENTRO DE ATENCAO ESPECIALIZADA	Analytical Competence Molecular Epidemiology Lab/ACME, Oswaldo Cruz Foundation, Ceara (FIOCRUZ CE)	Carlos Leonardo de Aragao Araujo; Cleber Furtado Aksenen; Fabio Miyajima; Fernando Braga Stehling; Igor Oliveira Duarte on behalf & Nicole Silva França of COVID-19 FIOCRUZ Genomic Network; Jamille Maria Mendes Bezerra; Joaquim Cesar do Nascimento Sousa Junior; Pedro Miguel Carneiro Jeronimo; Suzana Porto Almeida; Thais Ferreira de Oliveira; Thais de Oliveira Costa; Ticiane Cavalcante de Souza; Veridiana Pessoa Miyajima
EPI_ISL_8353528, EPI_ISL_8353558, EPI_ISL_8353587, EPI_ISL_8353588	CENTRO DE ATENDIMENTO A COVID	Instituto Butantan	Antonio Jorge Martins; Claudia Renata dos Santos Barros; David Schlesinger; Debora Botequio Moretti; Dimas Tadeu Covas; Elaine Cristina Marqueze; Elaine Vieira Santos; Evandra Strazza Rodrigues; Heidge Fukumasu; Jayme Augusto de Souza-Neto; José Salvatore Leister Patané; Luiz Alcantara; Luiz Lehmann Coutinho; Maria Carolina Elias; Maurício Lacerda Nogueira; Rafael dos Santos Bezerra; Raul Machado Neto; Rejane Maria Tommasini Grotto; Ricardo Haddad; Sandra Coccuzzo Sampaio Vessoni; Simone Kashima; Svetoslav Nanev Slavov; Vincent Louis Viala
EPI_ISL_8721676	CENTRO DE ESPECIALIDADES DE AGUAI AGUAI	Instituto Butantan	Antonio Jorge Martins; Claudia Renata dos Santos Barros; David Schlesinger; Debora Botequio Moretti; Dimas Tadeu Covas; Elaine Cristina Marqueze; Elaine Vieira Santos; Evandra Strazza Rodrigues; Heidge Fukumasu; Jayme Augusto de Souza-Neto; José Salvatore Leister Patané; Luiz Alcantara; Luiz Lehmann Coutinho; Maria Carolina Elias; Maurício Lacerda Nogueira; Rafael dos Santos Bezerra; Raul Machado Neto; Rejane Maria Tommasini Grotto; Ricardo Haddad; Sandra Coccuzzo Sampaio Vessoni; Simone Kashima; Svetoslav Nanev Slavov; Vincent Louis Viala
EPI_ISL_8184802, EPI_ISL_8184803, EPI_ISL_8184804	CENTRO DE GENOMAS	Instituto Butantan	Antonio Jorge Martins; Claudia Renata dos Santos Barros; David Schlesinger; Debora Botequio Moretti; Dimas Tadeu Covas; Elaine Cristina Marqueze; Elaine Vieira Santos; Evandra Strazza Rodrigues; Heidge Fukumasu; Jayme Augusto de Souza-Neto; José Salvatore Leister Patané; Luiz Alcantara; Luiz Lehmann Coutinho; Maria Carolina Elias; Maurício Lacerda Nogueira; Rafael dos Santos Bezerra; Raul Machado Neto; Rejane Maria Tommasini Grotto; Ricardo Haddad; Sandra Coccuzzo Sampaio Vessoni; Simone Kashima; Svetoslav Nanev Slavov; Vincent Louis Viala
EPI_ISL_8353541, EPI_ISL_8721397, EPI_ISL_8721717	CENTRO DE REFERENCIA E TREINAMENTO DSTAIDS SAO PAULO	Instituto Butantan	Antonio Jorge Martins; Claudia Renata dos Santos Barros; David Schlesinger; Debora Botequio Moretti; Dimas Tadeu Covas; Elaine Cristina Marqueze; Elaine Vieira Santos; Evandra Strazza Rodrigues; Heidge Fukumasu; Jayme Augusto de Souza-Neto; José Salvatore Leister Patané; Luiz Alcantara; Luiz Lehmann Coutinho; Maria Carolina Elias; Maurício Lacerda Nogueira; Rafael dos Santos Bezerra; Raul Machado Neto; Rejane Maria Tommasini Grotto; Ricardo Haddad; Sandra Coccuzzo Sampaio Vessoni; Simone Kashima; Svetoslav Nanev Slavov; Vincent Louis Viala
EPI_ISL_8721784, EPI_ISL_8721832	CENTRO DE SAUDE ANTONIA BERSI	Instituto Butantan	Antonio Jorge Martins; Claudia Renata dos Santos Barros; David Schlesinger; Debora Botequio Moretti; Dimas Tadeu Covas; Elaine Cristina Marqueze; Elaine Vieira Santos; Evandra Strazza Rodrigues; Heidge Fukumasu; Jayme Augusto de Souza-Neto; José Salvatore Leister Patané; Luiz Alcantara; Luiz Lehmann Coutinho; Maria Carolina Elias; Maurício Lacerda Nogueira; Rafael dos Santos Bezerra; Raul Machado Neto; Rejane Maria Tommasini Grotto; Ricardo Haddad; Sandra Coccuzzo Sampaio Vessoni; Simone Kashima; Svetoslav Nanev Slavov; Vincent Louis Viala
EPI_ISL_8721626, EPI_ISL_8721720, EPI_ISL_8721760, EPI_ISL_8721785	CENTRO DE SAUDE BARAO GERALDO	Instituto Butantan	Antonio Jorge Martins; Claudia Renata dos Santos Barros; David Schlesinger; Debora Botequio Moretti; Dimas Tadeu Covas; Elaine Cristina Marqueze; Elaine Vieira Santos; Evandra Strazza Rodrigues; Heidge Fukumasu; Jayme Augusto de Souza-Neto; José Salvatore Leister Patané; Luiz Alcantara; Luiz Lehmann Coutinho; Maria Carolina Elias; Maurício Lacerda Nogueira; Rafael dos Santos Bezerra; Raul Machado Neto; Rejane Maria Tommasini Grotto; Ricardo Haddad; Sandra Coccuzzo Sampaio Vessoni; Simone Kashima; Svetoslav Nanev Slavov; Vincent Louis Viala
EPI_ISL_8353542	CENTRO DE SAUDE BOA VISTA	Instituto Butantan	Antonio Jorge Martins; Claudia Renata dos Santos Barros; David Schlesinger; Debora Botequio Moretti; Dimas Tadeu Covas; Elaine Cristina Marqueze; Elaine Vieira Santos; Evandra Strazza Rodrigues; Heidge Fukumasu; Jayme Augusto de Souza-Neto; José Salvatore Leister Patané; Luiz Alcantara; Luiz Lehmann Coutinho; Maria Carolina Elias; Maurício Lacerda Nogueira; Rafael dos Santos Bezerra; Raul Machado Neto; Rejane Maria Tommasini Grotto; Ricardo Haddad; Sandra Coccuzzo Sampaio Vessoni; Simone Kashima; Svetoslav Nanev Slavov; Vincent Louis Viala
EPI_ISL_8721264, EPI_ISL_8721399, EPI_ISL_8721510	CENTRO DE SAUDE CAMPOS ELISEOS TANCREDO NEVES	Instituto Butantan	Antonio Jorge Martins; Claudia Renata dos Santos Barros; David Schlesinger; Debora Botequio Moretti; Dimas Tadeu Covas; Elaine Cristina Marqueze; Elaine Vieira Santos; Evandra Strazza Rodrigues; Heidge Fukumasu; Jayme Augusto de Souza-Neto; José Salvatore Leister Patané; Luiz Alcantara; Luiz Lehmann Coutinho; Maria Carolina Elias; Maurício Lacerda Nogueira; Rafael dos Santos Bezerra; Raul Machado Neto; Rejane Maria Tommasini Grotto; Ricardo Haddad; Sandra Coccuzzo Sampaio Vessoni; Simone Kashima; Svetoslav Nanev Slavov; Vincent Louis Viala
EPI_ISL_8721993	CENTRO DE SAUDE CENTRO	Instituto Butantan	Antonio Jorge Martins; Claudia Renata dos Santos Barros; David Schlesinger; Debora Botequio Moretti; Dimas Tadeu Covas; Elaine Cristina Marqueze; Elaine Vieira Santos; Evandra Strazza Rodrigues; Heidge Fukumasu; Jayme Augusto de Souza-Neto; José Salvatore Leister Patané; Luiz Alcantara; Luiz Lehmann Coutinho; Maria Carolina Elias; Maurício Lacerda Nogueira; Rafael dos Santos Bezerra; Raul Machado Neto; Rejane Maria Tommasini Grotto; Ricardo Haddad; Sandra Coccuzzo Sampaio Vessoni; Simone Kashima; Svetoslav Nanev Slavov; Vincent Louis Viala
EPI_ISL_8721339, EPI_ISL_8721371, EPI_ISL_8721487, EPI_ISL_8721623, EPI_ISL_8721895, EPI_ISL_8721968	CENTRO DE SAUDE COSTA E SILVA	Instituto Butantan	Antonio Jorge Martins; Claudia Renata dos Santos Barros; David Schlesinger; Debora Botequio Moretti; Dimas Tadeu Covas; Elaine Cristina Marqueze; Elaine Vieira Santos; Evandra Strazza Rodrigues; Heidge Fukumasu; Jayme Augusto de Souza-Neto; José Salvatore Leister Patané; Luiz Alcantara; Luiz Lehmann Coutinho; Maria Carolina Elias; Maurício Lacerda Nogueira; Rafael dos Santos Bezerra; Raul Machado Neto; Rejane Maria Tommasini Grotto; Ricardo Haddad; Sandra Coccuzzo Sampaio Vessoni; Simone Kashima; Svetoslav Nanev Slavov; Vincent Louis Viala
EPI_ISL_8721284, EPI_ISL_8721298, EPI_ISL_8721351, EPI_ISL_8721890, EPI_ISL_8721951	CENTRO DE SAUDE DE ADAMANTINA	Instituto Butantan	Antonio Jorge Martins; Claudia Renata dos Santos Barros; David Schlesinger; Debora Botequio Moretti; Dimas Tadeu Covas; Elaine Cristina Marqueze; Elaine Vieira Santos; Evandra Strazza Rodrigues; Heidge Fukumasu; Jayme Augusto de Souza-Neto; José Salvatore Leister Patané; Luiz Alcantara; Luiz Lehmann Coutinho; Maria Carolina Elias; Maurício Lacerda Nogueira; Rafael dos Santos Bezerra; Raul Machado Neto; Rejane Maria Tommasini Grotto; Ricardo Haddad; Sandra Coccuzzo Sampaio Vessoni; Simone Kashima; Svetoslav Nanev Slavov; Vincent Louis Viala
EPI_ISL_8353577, EPI_ISL_8721333, EPI_ISL_8721932	CENTRO DE SAUDE DE RIBEIRAO DO SUL	Instituto Butantan	Antonio Jorge Martins; Claudia Renata dos Santos Barros; David Schlesinger; Debora Botequio Moretti; Dimas Tadeu Covas; Elaine Cristina Marqueze; Elaine Vieira Santos; Evandra Strazza Rodrigues; Heidge Fukumasu; Jayme Augusto de Souza-Neto; José Salvatore Leister Patané; Luiz Alcantara; Luiz Lehmann Coutinho; Maria Carolina Elias; Maurício Lacerda Nogueira; Rafael dos Santos Bezerra; Raul Machado Neto; Rejane Maria Tommasini Grotto; Ricardo Haddad; Sandra Coccuzzo Sampaio Vessoni; Simone Kashima; Svetoslav Nanev Slavov; Vincent Louis Viala
EPI_ISL_8721707	CENTRO DE SAUDE DE SPEDRO DO TURVO	Instituto Butantan	Antonio Jorge Martins; Claudia Renata dos Santos Barros; David Schlesinger; Debora Botequio Moretti; Dimas Tadeu Covas; Elaine Cristina Marqueze; Elaine Vieira Santos; Evandra Strazza Rodrigues; Heidge Fukumasu; Jayme Augusto de Souza-Neto; José Salvatore Leister Patané; Luiz Alcantara; Luiz Lehmann Coutinho; Maria Carolina Elias; Maurício Lacerda Nogueira; Rafael dos Santos Bezerra; Raul Machado Neto; Rejane Maria Tommasini Grotto; Ricardo Haddad; Sandra Coccuzzo Sampaio Vessoni; Simone Kashima; Svetoslav Nanev Slavov; Vincent Louis Viala
EPI_ISL_8721263	CENTRO DE SAUDE DIC III	Instituto Butantan	Antonio Jorge Martins; Claudia Renata dos Santos Barros; David Schlesinger; Debora Botequio Moretti; Dimas Tadeu Covas; Elaine Cristina Marqueze; Elaine Vieira Santos; Evandra Strazza Rodrigues; Heidge Fukumasu; Jayme Augusto de Souza-Neto; José Salvatore Leister Patané; Luiz Alcantara; Luiz Lehmann Coutinho; Maria Carolina Elias; Maurício Lacerda Nogueira; Rafael dos Santos Bezerra; Raul Machado Neto; Rejane Maria Tommasini Grotto; Ricardo Haddad; Sandra Coccuzzo Sampaio Vessoni; Simone Kashima; Svetoslav Nanev Slavov; Vincent Louis Viala
EPI_ISL_8721857	CENTRO DE SAUDE FARIA LIMA	Instituto Butantan	Antonio Jorge Martins; Claudia Renata dos Santos Barros; David Schlesinger; Debora Botequio Moretti; Dimas Tadeu Covas; Elaine Cristina Marqueze; Elaine Vieira Santos; Evandra Strazza Rodrigues; Heidge Fukumasu; Jayme Augusto de Souza-Neto; José Salvatore Leister Patané; Luiz Alcantara; Luiz Lehmann Coutinho; Maria Carolina Elias; Maurício Lacerda Nogueira; Rafael dos Santos Bezerra; Raul Machado Neto; Rejane Maria Tommasini Grotto; Ricardo Haddad; Sandra Coccuzzo Sampaio Vessoni; Simone Kashima; Svetoslav Nanev Slavov; Vincent Louis Viala
EPI_ISL_8721632	CENTRO DE SAUDE I AVARE	Instituto Butantan	Antonio Jorge Martins; Claudia Renata dos Santos Barros; David Schlesinger; Debora Botequio Moretti; Dimas Tadeu Covas; Elaine Cristina Marqueze; Elaine Vieira Santos; Evandra Strazza Rodrigues; Heidge Fukumasu; Jayme Augusto de Souza-Neto; José Salvatore Leister Patané; Luiz Alcantara; Luiz Lehmann Coutinho; Maria Carolina Elias; Maurício Lacerda Nogueira; Rafael dos Santos Bezerra; Raul Machado Neto; Rejane Maria Tommasini Grotto; Ricardo Haddad; Sandra Coccuzzo Sampaio Vessoni; Simone Kashima; Svetoslav Nanev Slavov; Vincent Louis

			Viala
EPI_ISL_8184829, EPI_ISL_8353532, EPI_ISL_8353534, EPI_ISL_8353594, EPI_ISL_8353595	CENTRO DE SAUDE III DE SERRANA	Instituto Butantan	Antonio Jorge Martins; Claudia Renata dos Santos Barros; David Schlesinger; Debora Botequio Moretti; Dimas Tadeu Covas; Elaine Cristina Marqueze; Elaine Vieira Santos; Evandra Strazza Rodrigues; Heidge Fukumasu; Jayme Augusto de Souza-Neto; José Salvatore Leister Patané; Luiz Alcantara; Luiz Lehmann Coutinho; Maria Carolina Elias; Maurício Lacerda Nogueira; Rafael dos Santos Bezerra; Raul Machado Neto; Rejane Maria Tommasini Grotto; Ricardo Haddad; Sandra Coccuzzo Sampaio Vessoni; Simone Kashima; Svetoslav Nanev Slavov; Vincent Louis Viala
EPI_ISL_8721962	CENTRO DE SAUDE INTEGRACAO	Instituto Butantan	Antonio Jorge Martins; Claudia Renata dos Santos Barros; David Schlesinger; Debora Botequio Moretti; Dimas Tadeu Covas; Elaine Cristina Marqueze; Elaine Vieira Santos; Evandra Strazza Rodrigues; Heidge Fukumasu; Jayme Augusto de Souza-Neto; José Salvatore Leister Patané; Luiz Alcantara; Luiz Lehmann Coutinho; Maria Carolina Elias; Maurício Lacerda Nogueira; Rafael dos Santos Bezerra; Raul Machado Neto; Rejane Maria Tommasini Grotto; Ricardo Haddad; Sandra Coccuzzo Sampaio Vessoni; Simone Kashima; Svetoslav Nanev Slavov; Vincent Louis Viala
EPI_ISL_8721601	CENTRO DE SAUDE JARDIM AEROPORTO	Instituto Butantan	Antonio Jorge Martins; Claudia Renata dos Santos Barros; David Schlesinger; Debora Botequio Moretti; Dimas Tadeu Covas; Elaine Cristina Marqueze; Elaine Vieira Santos; Evandra Strazza Rodrigues; Heidge Fukumasu; Jayme Augusto de Souza-Neto; José Salvatore Leister Patané; Luiz Alcantara; Luiz Lehmann Coutinho; Maria Carolina Elias; Maurício Lacerda Nogueira; Rafael dos Santos Bezerra; Raul Machado Neto; Rejane Maria Tommasini Grotto; Ricardo Haddad; Sandra Coccuzzo Sampaio Vessoni; Simone Kashima; Svetoslav Nanev Slavov; Vincent Louis Viala
EPI_ISL_8721212, EPI_ISL_8721242, EPI_ISL_8721605	CENTRO DE SAUDE JARDIM FLORENCE	Instituto Butantan	Antonio Jorge Martins; Claudia Renata dos Santos Barros; David Schlesinger; Debora Botequio Moretti; Dimas Tadeu Covas; Elaine Cristina Marqueze; Elaine Vieira Santos; Evandra Strazza Rodrigues; Heidge Fukumasu; Jayme Augusto de Souza-Neto; José Salvatore Leister Patané; Luiz Alcantara; Luiz Lehmann Coutinho; Maria Carolina Elias; Maurício Lacerda Nogueira; Rafael dos Santos Bezerra; Raul Machado Neto; Rejane Maria Tommasini Grotto; Ricardo Haddad; Sandra Coccuzzo Sampaio Vessoni; Simone Kashima; Svetoslav Nanev Slavov; Vincent Louis Viala
EPI_ISL_8721961	CENTRO DE SAUDE JARDIM IPAUSSURAMA	Instituto Butantan	Antonio Jorge Martins; Claudia Renata dos Santos Barros; David Schlesinger; Debora Botequio Moretti; Dimas Tadeu Covas; Elaine Cristina Marqueze; Elaine Vieira Santos; Evandra Strazza Rodrigues; Heidge Fukumasu; Jayme Augusto de Souza-Neto; José Salvatore Leister Patané; Luiz Alcantara; Luiz Lehmann Coutinho; Maria Carolina Elias; Maurício Lacerda Nogueira; Rafael dos Santos Bezerra; Raul Machado Neto; Rejane Maria Tommasini Grotto; Ricardo Haddad; Sandra Coccuzzo Sampaio Vessoni; Simone Kashima; Svetoslav Nanev Slavov; Vincent Louis Viala
EPI_ISL_8721425	CENTRO DE SAUDE JARDIM SAO MARCOS	Instituto Butantan	Antonio Jorge Martins; Claudia Renata dos Santos Barros; David Schlesinger; Debora Botequio Moretti; Dimas Tadeu Covas; Elaine Cristina Marqueze; Elaine Vieira Santos; Evandra Strazza Rodrigues; Heidge Fukumasu; Jayme Augusto de Souza-Neto; José Salvatore Leister Patané; Luiz Alcantara; Luiz Lehmann Coutinho; Maria Carolina Elias; Maurício Lacerda Nogueira; Rafael dos Santos Bezerra; Raul Machado Neto; Rejane Maria Tommasini Grotto; Ricardo Haddad; Sandra Coccuzzo Sampaio Vessoni; Simone Kashima; Svetoslav Nanev Slavov; Vincent Louis Viala
EPI_ISL_8721588	CENTRO DE SAUDE NOVA AMERICA	Instituto Butantan	Antonio Jorge Martins; Claudia Renata dos Santos Barros; David Schlesinger; Debora Botequio Moretti; Dimas Tadeu Covas; Elaine Cristina Marqueze; Elaine Vieira Santos; Evandra Strazza Rodrigues; Heidge Fukumasu; Jayme Augusto de Souza-Neto; José Salvatore Leister Patané; Luiz Alcantara; Luiz Lehmann Coutinho; Maria Carolina Elias; Maurício Lacerda Nogueira; Rafael dos Santos Bezerra; Raul Machado Neto; Rejane Maria Tommasini Grotto; Ricardo Haddad; Sandra Coccuzzo Sampaio Vessoni; Simone Kashima; Svetoslav Nanev Slavov; Vincent Louis Viala
EPI_ISL_8721891	CENTRO DE SAUDE OZIEL	Instituto Butantan	Antonio Jorge Martins; Claudia Renata dos Santos Barros; David Schlesinger; Debora Botequio Moretti; Dimas Tadeu Covas; Elaine Cristina Marqueze; Elaine Vieira Santos; Evandra Strazza Rodrigues; Heidge Fukumasu; Jayme Augusto de Souza-Neto; José Salvatore Leister Patané; Luiz Alcantara; Luiz Lehmann Coutinho; Maria Carolina Elias; Maurício Lacerda Nogueira; Rafael dos Santos Bezerra; Raul Machado Neto; Rejane Maria Tommasini Grotto; Ricardo Haddad; Sandra Coccuzzo Sampaio Vessoni; Simone Kashima; Svetoslav Nanev Slavov; Vincent Louis Viala
EPI_ISL_8721688, EPI_ISL_8721884, EPI_ISL_8721969	CENTRO DE SAUDE PARANAPANEMA	Instituto Butantan	Antonio Jorge Martins; Claudia Renata dos Santos Barros; David Schlesinger; Debora Botequio Moretti; Dimas Tadeu Covas; Elaine Cristina Marqueze; Elaine Vieira Santos; Evandra Strazza Rodrigues; Heidge Fukumasu; Jayme Augusto de Souza-Neto; José Salvatore Leister Patané; Luiz Alcantara; Luiz Lehmann Coutinho; Maria Carolina Elias; Maurício Lacerda Nogueira; Rafael dos Santos Bezerra; Raul Machado Neto; Rejane Maria Tommasini Grotto; Ricardo Haddad; Sandra Coccuzzo Sampaio Vessoni; Simone Kashima; Svetoslav Nanev Slavov; Vincent Louis Viala
EPI_ISL_8721286	CENTRO DE SAUDE PARQUE FLORESTA	Instituto Butantan	Antonio Jorge Martins; Claudia Renata dos Santos Barros; David Schlesinger; Debora Botequio Moretti; Dimas Tadeu Covas; Elaine Cristina Marqueze; Elaine Vieira Santos; Evandra Strazza Rodrigues; Heidge Fukumasu; Jayme Augusto de Souza-Neto; José Salvatore Leister Patané; Luiz Alcantara; Luiz Lehmann Coutinho; Maria Carolina Elias; Maurício Lacerda Nogueira; Rafael dos Santos Bezerra; Raul Machado Neto; Rejane Maria Tommasini Grotto; Ricardo Haddad; Sandra Coccuzzo Sampaio Vessoni; Simone Kashima; Svetoslav Nanev Slavov; Vincent Louis Viala
EPI_ISL_8721488	CENTRO DE SAUDE PARQUE SANTA BARBARA	Instituto Butantan	Antonio Jorge Martins; Claudia Renata dos Santos Barros; David Schlesinger; Debora Botequio Moretti; Dimas Tadeu Covas; Elaine Cristina Marqueze; Elaine Vieira Santos; Evandra Strazza Rodrigues; Heidge Fukumasu; Jayme Augusto de Souza-Neto; José Salvatore Leister Patané; Luiz Alcantara; Luiz Lehmann Coutinho; Maria Carolina Elias; Maurício Lacerda Nogueira; Rafael dos Santos Bezerra; Raul Machado Neto; Rejane Maria Tommasini Grotto; Ricardo Haddad; Sandra Coccuzzo Sampaio Vessoni; Simone Kashima; Svetoslav Nanev Slavov; Vincent Louis Viala
EPI_ISL_8721940	CENTRO DE SAUDE PERSEU LEITE DE BARROS	Instituto Butantan	Antonio Jorge Martins; Claudia Renata dos Santos Barros; David Schlesinger; Debora Botequio Moretti; Dimas Tadeu Covas; Elaine Cristina Marqueze; Elaine Vieira Santos; Evandra Strazza Rodrigues; Heidge Fukumasu; Jayme Augusto de Souza-Neto; José Salvatore Leister Patané; Luiz Alcantara; Luiz Lehmann Coutinho; Maria Carolina Elias; Maurício Lacerda Nogueira; Rafael dos Santos Bezerra; Raul Machado Neto; Rejane Maria Tommasini Grotto; Ricardo Haddad; Sandra Coccuzzo Sampaio Vessoni; Simone Kashima; Svetoslav Nanev Slavov; Vincent Louis Viala
EPI_ISL_8721517	CENTRO DE SAUDE SANTA ODILA	Instituto Butantan	Antonio Jorge Martins; Claudia Renata dos Santos Barros; David Schlesinger; Debora Botequio Moretti; Dimas Tadeu Covas; Elaine Cristina Marqueze; Elaine Vieira Santos; Evandra Strazza Rodrigues; Heidge Fukumasu; Jayme Augusto de Souza-Neto; José Salvatore Leister Patané; Luiz Alcantara; Luiz Lehmann Coutinho; Maria Carolina Elias; Maurício Lacerda Nogueira; Rafael dos Santos Bezerra; Raul Machado Neto; Rejane Maria Tommasini Grotto; Ricardo Haddad; Sandra Coccuzzo Sampaio Vessoni; Simone Kashima; Svetoslav Nanev Slavov; Vincent Louis Viala
EPI_ISL_8721382, EPI_ISL_8721414	CENTRO DE SAUDE SAO CRISTOVAO	Instituto Butantan	Antonio Jorge Martins; Claudia Renata dos Santos Barros; David Schlesinger; Debora Botequio Moretti; Dimas Tadeu Covas; Elaine Cristina Marqueze; Elaine Vieira Santos; Evandra Strazza Rodrigues; Heidge Fukumasu; Jayme Augusto de Souza-Neto; José Salvatore Leister Patané; Luiz Alcantara; Luiz Lehmann Coutinho; Maria Carolina Elias; Maurício Lacerda Nogueira; Rafael dos Santos Bezerra; Raul Machado Neto; Rejane Maria Tommasini Grotto; Ricardo Haddad; Sandra Coccuzzo Sampaio Vessoni; Simone Kashima; Svetoslav Nanev Slavov; Vincent Louis Viala
EPI_ISL_8353537	CENTRO DE SAUDE SAO QUIRINO	Instituto Butantan	Antonio Jorge Martins; Claudia Renata dos Santos Barros; David Schlesinger; Debora Botequio Moretti; Dimas Tadeu Covas; Elaine Cristina Marqueze; Elaine Vieira Santos; Evandra Strazza Rodrigues; Heidge Fukumasu; Jayme Augusto de Souza-Neto; José Salvatore Leister Patané; Luiz Alcantara; Luiz Lehmann Coutinho; Maria Carolina Elias; Maurício Lacerda Nogueira; Rafael dos Santos Bezerra; Raul Machado Neto; Rejane Maria Tommasini Grotto; Ricardo Haddad; Sandra Coccuzzo Sampaio Vessoni; Simone Kashima; Svetoslav Nanev Slavov; Vincent Louis Viala
EPI_ISL_8721624	CENTRO DE SAUDE SOUSAS	Instituto Butantan	Antonio Jorge Martins; Claudia Renata dos Santos Barros; David Schlesinger; Debora Botequio Moretti; Dimas Tadeu Covas; Elaine Cristina Marqueze; Elaine Vieira Santos; Evandra Strazza Rodrigues; Heidge Fukumasu; Jayme Augusto de Souza-Neto; José Salvatore Leister Patané; Luiz Alcantara; Luiz Lehmann Coutinho; Maria Carolina Elias; Maurício Lacerda Nogueira; Rafael dos Santos Bezerra; Raul Machado Neto; Rejane Maria Tommasini Grotto; Ricardo Haddad; Sandra Coccuzzo Sampaio Vessoni; Simone Kashima; Svetoslav Nanev Slavov; Vincent Louis Viala
EPI_ISL_8721835	CENTRO DE SAUDE VILA 31 DE MARCO	Instituto Butantan	Antonio Jorge Martins; Claudia Renata dos Santos Barros; David Schlesinger; Debora Botequio Moretti; Dimas Tadeu Covas; Elaine Cristina Marqueze; Elaine Vieira Santos; Evandra Strazza Rodrigues; Heidge Fukumasu; Jayme Augusto de Souza-Neto; José Salvatore Leister Patané; Luiz Alcantara; Luiz Lehmann Coutinho; Maria Carolina Elias; Maurício Lacerda Nogueira; Rafael dos Santos Bezerra; Raul Machado Neto; Rejane Maria Tommasini Grotto; Ricardo Haddad; Sandra Coccuzzo Sampaio Vessoni; Simone Kashima; Svetoslav Nanev Slavov; Vincent Louis Viala
EPI_ISL_8721305, EPI_ISL_8721413, EPI_ISL_8721694, EPI_ISL_8721743	CENTRO DE SAUDE VILA IPE	Instituto Butantan	Antonio Jorge Martins; Claudia Renata dos Santos Barros; David Schlesinger; Debora Botequio Moretti; Dimas Tadeu Covas; Elaine Cristina Marqueze; Elaine Vieira Santos; Evandra Strazza Rodrigues; Heidge Fukumasu; Jayme Augusto de Souza-Neto; José Salvatore Leister Patané; Luiz Alcantara; Luiz Lehmann Coutinho; Maria Carolina Elias; Maurício Lacerda Nogueira; Rafael dos Santos Bezerra; Raul Machado Neto; Rejane Maria Tommasini Grotto; Ricardo Haddad; Sandra Coccuzzo Sampaio Vessoni; Simone Kashima; Svetoslav Nanev Slavov; Vincent Louis Viala
EPI_ISL_8721869	CENTRO DE SAUDE VILA RICA	Instituto Butantan	Antonio Jorge Martins; Claudia Renata dos Santos Barros; David Schlesinger; Debora Botequio Moretti; Dimas Tadeu Covas; Elaine Cristina Marqueze; Elaine Vieira Santos; Evandra Strazza Rodrigues; Heidge Fukumasu; Jayme Augusto de Souza-Neto; José Salvatore Leister Patané; Luiz Alcantara; Luiz Lehmann Coutinho; Maria Carolina Elias; Maurício Lacerda Nogueira; Rafael dos Santos Bezerra; Raul Machado Neto; Rejane Maria Tommasini Grotto; Ricardo Haddad; Sandra Coccuzzo Sampaio Vessoni; Simone Kashima; Svetoslav Nanev Slavov; Vincent Louis Viala
EPI_ISL_8721735	CENTRO DE SAUDE VISTA ALEGRE	Instituto Butantan	Antonio Jorge Martins; Claudia Renata dos Santos Barros; David Schlesinger; Debora Botequio Moretti; Dimas Tadeu Covas; Elaine Cristina Marqueze; Elaine Vieira Santos; Evandra Strazza Rodrigues; Heidge Fukumasu; Jayme Augusto de Souza-Neto; José Salvatore Leister Patané; Luiz Alcantara; Luiz Lehmann Coutinho; Maria Carolina Elias; Maurício Lacerda Nogueira; Rafael dos Santos Bezerra; Raul Machado Neto; Rejane Maria Tommasini Grotto; Ricardo Haddad; Sandra Coccuzzo Sampaio Vessoni; Simone Kashima; Svetoslav Nanev Slavov; Vincent Louis Viala
EPI_ISL_8465114	CENTRO DE TRIAGEM COVID 19	Instituto Butantan	Antonio Jorge Martins; Claudia Renata dos Santos Barros; David Schlesinger; Debora Botequio Moretti; Dimas Tadeu Covas; Elaine Cristina Marqueze; Elaine Vieira Santos; Evandra Strazza Rodrigues; Heidge Fukumasu; Jayme Augusto de Souza-Neto; José Salvatore Leister Patané; Luiz Alcantara; Luiz Lehmann Coutinho; Maria Carolina Elias; Maurício Lacerda Nogueira; Rafael dos Santos Bezerra; Raul Machado Neto; Rejane Maria Tommasini Grotto; Ricardo Haddad; Sandra Coccuzzo Sampaio Vessoni; Simone Kashima; Svetoslav Nanev Slavov; Vincent Louis Viala
EPI_ISL_8721524	CENTRO MEDICO PMESP	Instituto Butantan	Antonio Jorge Martins; Claudia Renata dos Santos Barros; David Schlesinger; Debora Botequio Moretti; Dimas Tadeu Covas; Elaine Cristina Marqueze; Elaine Vieira Santos; Evandra Strazza Rodrigues; Heidge Fukumasu; Jayme Augusto de Souza-Neto; José Salvatore Leister Patané; Luiz Alcantara; Luiz Lehmann Coutinho; Maria Carolina Elias; Maurício Lacerda Nogueira; Rafael dos Santos Bezerra; Raul Machado Neto; Rejane Maria Tommasini Grotto; Ricardo Haddad; Sandra Coccuzzo Sampaio Vessoni; Simone Kashima; Svetoslav Nanev Slavov; Vincent Louis Viala
EPI_ISL_8353564, EPI_ISL_8353606	CENTRO MEDICO SOCIAL COMUNITARIO JANUARIO TEODORO DE SOUZA	Instituto Butantan	Antonio Jorge Martins; Claudia Renata dos Santos Barros; David Schlesinger; Debora Botequio Moretti; Dimas Tadeu Covas; Elaine Cristina Marqueze; Elaine Vieira Santos; Evandra Strazza Rodrigues; Heidge Fukumasu; Jayme Augusto de Souza-Neto; José Salvatore Leister Patané; Luiz Alcantara; Luiz Lehmann Coutinho; Maria Carolina Elias; Maurício Lacerda Nogueira; Rafael dos Santos Bezerra; Raul Machado Neto; Rejane Maria Tommasini Grotto; Ricardo Haddad; Sandra Coccuzzo Sampaio Vessoni; Simone Kashima; Svetoslav Nanev Slavov; Vincent Louis Viala
EPI_ISL_8721225	CLINICA SALTO SAUDE SANTA CRUZ	Instituto Butantan	Antonio Jorge Martins; Claudia Renata dos Santos Barros; David Schlesinger; Debora Botequio Moretti; Dimas Tadeu Covas; Elaine Cristina Marqueze; Elaine Vieira Santos; Evandra Strazza Rodrigues; Heidge Fukumasu; Jayme Augusto de Souza-Neto; José Salvatore Leister Patané; Luiz Alcantara; Luiz Lehmann Coutinho; Maria Carolina Elias; Maurício Lacerda Nogueira; Rafael dos Santos Bezerra; Raul Machado Neto; Rejane Maria Tommasini Grotto; Ricardo Haddad; Sandra Coccuzzo Sampaio Vessoni; Simone Kashima; Svetoslav Nanev Slavov; Vincent Louis Viala
EPI_ISL_8721428	COMPLEXO HOSPITALAR OURO VERDE DE CAMPINAS	Instituto Butantan	Antonio Jorge Martins; Claudia Renata dos Santos Barros; David Schlesinger; Debora Botequio Moretti; Dimas Tadeu Covas; Elaine Cristina Marqueze; Elaine Vieira Santos; Evandra Strazza Rodrigues; Heidge Fukumasu; Jayme Augusto de Souza-Neto; José Salvatore Leister Patané; Luiz Alcantara; Luiz Lehmann Coutinho; Maria Carolina Elias; Maurício Lacerda Nogueira; Rafael dos Santos Bezerra; Raul Machado Neto; Rejane Maria Tommasini Grotto; Ricardo Haddad; Sandra Coccuzzo Sampaio Vessoni; Simone Kashima; Svetoslav Nanev Slavov; Vincent Louis Viala
EPI_ISL_8353539, EPI_ISL_8353544, EPI_ISL_8353551, EPI_ISL_8353552, EPI_ISL_8353573, EPI_ISL_8353574, EPI_ISL_8353578, EPI_ISL_8353582, EPI_ISL_8721462, EPI_ISL_8721521, EPI_ISL_8721604			
see above	COVID 19 CENTRO DE COMBATE AO CORONAVIRUS CCC JANDIRA	Instituto Butantan	Antonio Jorge Martins; Claudia Renata dos Santos Barros; David Schlesinger; Debora Botequio Moretti; Dimas Tadeu Covas; Elaine Cristina Marqueze; Elaine Vieira Santos; Evandra Strazza Rodrigues; Heidge Fukumasu; Jayme Augusto de Souza-Neto; José Salvatore Leister Patané; Luiz Alcantara; Luiz Lehmann Coutinho; Maria Carolina Elias; Maurício Lacerda Nogueira; Rafael dos Santos Bezerra; Raul Machado Neto; Rejane Maria Tommasini Grotto; Ricardo Haddad; Sandra Coccuzzo Sampaio Vessoni; Simone Kashima; Svetoslav Nanev Slavov; Vincent Louis Viala
EPI_ISL_8353572	CS DE ADOLFO	Instituto Butantan	Antonio Jorge Martins; Claudia Renata dos Santos Barros; David Schlesinger; Debora Botequio Moretti; Dimas Tadeu Covas; Elaine Cristina Marqueze; Elaine Vieira Santos; Evandra Strazza Rodrigues; Heidge Fukumasu; Jayme Augusto de Souza-Neto; José Salvatore Leister Patané; Luiz Alcantara; Luiz Lehmann Coutinho; Maria Carolina Elias; Maurício Lacerda Nogueira; Rafael dos Santos Bezerra; Raul Machado Neto; Rejane Maria Tommasini Grotto; Ricardo Haddad; Sandra Coccuzzo Sampaio Vessoni; Simone Kashima; Svetoslav Nanev Slavov; Vincent Louis Viala
EPI_ISL_8721536	CS DE CAMPOS NOVOS PTA	Instituto Butantan	Antonio Jorge Martins; Claudia Renata dos Santos Barros; David Schlesinger; Debora Botequio Moretti; Dimas Tadeu Covas; Elaine Cristina Marqueze; Elaine Vieira Santos; Evandra Strazza Rodrigues; Heidge Fukumasu; Jayme Augusto de Souza-Neto; José Salvatore Leister Patané; Luiz Alcantara; Luiz Lehmann Coutinho; Maria Carolina Elias; Maurício Lacerda Nogueira; Rafael dos Santos Bezerra; Raul Machado Neto; Rejane Maria Tommasini Grotto; Ricardo Haddad; Sandra Coccuzzo Sampaio Vessoni; Simone Kashima; Svetoslav Nanev Slavov; Vincent Louis Viala
EPI_ISL_8721301, EPI_ISL_8721897	CS DE MIRASSOL	Instituto Butantan	Antonio Jorge Martins; Claudia Renata dos Santos Barros; David Schlesinger; Debora Botequio Moretti; Dimas Tadeu Covas; Elaine Cristina Marqueze; Elaine Vieira Santos; Evandra Strazza Rodrigues; Heidge Fukumasu; Jayme Augusto de Souza-Neto; José Salvatore Leister Patané; Luiz Alcantara; Luiz Lehmann Coutinho; Maria Carolina Elias; Maurício Lacerda Nogueira; Rafael dos Santos Bezerra; Raul Machado Neto; Rejane Maria Tommasini Grotto; Ricardo Haddad; Sandra Coccuzzo Sampaio Vessoni; Simone Kashima; Svetoslav Nanev Slavov; Vincent Louis Viala
EPI_ISL_8721836	CS IACRI	Instituto Butantan	Antonio Jorge Martins; Claudia Renata dos Santos Barros; David Schlesinger; Debora Botequio Moretti; Dimas Tadeu Covas; Elaine Cristina Marqueze; Elaine Vieira Santos; Evandra Strazza Rodrigues; Heidge Fukumasu; Jayme Augusto de Souza-Neto; José Salvatore Leister Patané; Luiz Alcantara; Luiz Lehmann Coutinho; Maria Carolina Elias; Maurício Lacerda Nogueira; Rafael dos Santos Bezerra; Raul Machado Neto; Rejane Maria Tommasini Grotto; Ricardo Haddad; Sandra Coccuzzo Sampaio Vessoni; Simone Kashima; Svetoslav Nanev Slavov; Vincent Louis Viala
EPI_ISL_8721336, EPI_ISL_8721587, EPI_ISL_8721620, EPI_ISL_8721691, EPI_ISL_8721816, EPI_ISL_8721833, EPI_ISL_8721834			
see above	CS II DR JOSE CARQUEIJO SCRPARDO	Instituto Butantan	Antonio Jorge Martins; Claudia Renata dos Santos Barros; David Schlesinger; Debora Botequio Moretti; Dimas Tadeu Covas; Elaine Cristina Marqueze; Elaine Vieira Santos; Evandra Strazza Rodrigues; Heidge Fukumasu; Jayme Augusto de Souza-Neto; José Salvatore Leister Patané; Luiz Alcantara; Luiz Lehmann Coutinho; Maria Carolina Elias; Maurício Lacerda Nogueira; Rafael dos Santos Bezerra; Raul Machado Neto; Rejane Maria Tommasini Grotto; Ricardo Haddad; Sandra Coccuzzo Sampaio Vessoni; Simone Kashima; Svetoslav Nanev Slavov; Vincent Louis Viala

EPI_ISL_8721285, EPI_ISL_8721921	CS SES GERALDO DE PAULA SOUZA	Instituto Butantan	Antonio Jorge Martins; Claudia Renata dos Santos Barros; David Schlesinger; Debora Botequio Moretti; Dimas Tadeu Covas; Elaine Cristina Marqueze; Elaine Vieira Santos; Evandra Strazza Rodrigues; Heidge Fukumasu; Jayme Augusto de Souza-Neto; José Salvatore Leister Patané; Luiz Alcantara; Luiz Lehmann Coutinho; Maria Carolina Elias; Maurício Lacerda Nogueira; Rafael dos Santos Bezerra; Raul Machado Neto; Rejane Maria Tommasini Grotto; Ricardo Haddad; Sandra Coccuzzo Sampaio Vessoni; Simone Kashima; Svetoslav Nanev Slavov; Vincent Louis Viala
EPI_ISL_7899761	CS de Mirassol	Instituto Butantan	Antonio Jorge Martins; Claudia Renata dos Santos Barros; David Schlesinger; Debora Botequio Moretti; Dimas Tadeu Covas; Elaine Cristina Marqueze; Elaine Vieira Santos; Evandra Strazza Rodrigues; Heidge Fukumasu; Jayme Augusto de Souza-Neto; José Salvatore Leister Patané; Luiz Alcantara; Luiz Lehmann Coutinho; Maria Carolina Elias; Maurício Lacerda Nogueira; Rafael dos Santos Bezerra; Raul Machado Neto; Rejane Maria Tommasini Grotto; Ricardo Haddad; Sandra Coccuzzo Sampaio Vessoni; Simone Kashima; Svetoslav Nanev Slavov; Vincent Louis Viala
EPI_ISL_8622821, EPI_ISL_8622826, EPI_ISL_8622828, EPI_ISL_8622830, EPI_ISL_8622833, EPI_ISL_8622840, EPI_ISL_8622841, EPI_ISL_8622842, EPI_ISL_8622843, EPI_ISL_8622844, EPI_ISL_8622845, EPI_ISL_8622847			
see above	Central Public Health Laboratory - LACEN -Bahia, Salvador, Brazil	Central Public Health Laboratory - LACEN - Bahia, Salvador, Brazil	Arabela Leal; Felicidade Pereira; Gabriela Menezes; Jaqueline Gomes; Jessica Araujo; Luciana Oliveira; Luiz Alcantara; Marcela Gómez; Marta Giovanetti; Vagner Fonseca; Vanessa Nardy
EPI_ISL_8124024, EPI_ISL_8124025, EPI_ISL_8124026, EPI_ISL_8124027, EPI_ISL_8124028	DASA	DASA	Adriano Bonaldi; Angelica Hristov; Annelise Lopes; Bianca Cota; Camila Romano; Cristina Oliveira; Jose Levi; Lidia Yamamoto; Luciane Sussuchi; Paulo Pierry; Rodrigo Guarischi; Rodrigo Salazar
EPI_ISL_8721221, EPI_ISL_8721618, EPI_ISL_8722006	DEPARTAMENTO MUNICIPAL DE SAUDE CAJATI	Instituto Butantan	Antonio Jorge Martins; Claudia Renata dos Santos Barros; David Schlesinger; Debora Botequio Moretti; Dimas Tadeu Covas; Elaine Cristina Marqueze; Elaine Vieira Santos; Evandra Strazza Rodrigues; Heidge Fukumasu; Jayme Augusto de Souza-Neto; José Salvatore Leister Patané; Luiz Alcantara; Luiz Lehmann Coutinho; Maria Carolina Elias; Maurício Lacerda Nogueira; Rafael dos Santos Bezerra; Raul Machado Neto; Rejane Maria Tommasini Grotto; Ricardo Haddad; Sandra Coccuzzo Sampaio Vessoni; Simone Kashima; Svetoslav Nanev Slavov; Vincent Louis Viala
EPI_ISL_8721390	DEPARTAMENTO MUNICIPAL DE SAUDE DE BURITAMA	Instituto Butantan	Antonio Jorge Martins; Claudia Renata dos Santos Barros; David Schlesinger; Debora Botequio Moretti; Dimas Tadeu Covas; Elaine Cristina Marqueze; Elaine Vieira Santos; Evandra Strazza Rodrigues; Heidge Fukumasu; Jayme Augusto de Souza-Neto; José Salvatore Leister Patané; Luiz Alcantara; Luiz Lehmann Coutinho; Maria Carolina Elias; Maurício Lacerda Nogueira; Rafael dos Santos Bezerra; Raul Machado Neto; Rejane Maria Tommasini Grotto; Ricardo Haddad; Sandra Coccuzzo Sampaio Vessoni; Simone Kashima; Svetoslav Nanev Slavov; Vincent Louis Viala
EPI_ISL_8184798, EPI_ISL_8184799, EPI_ISL_8184800, EPI_ISL_8353565, EPI_ISL_8353566, EPI_ISL_8721216, EPI_ISL_8721227, EPI_ISL_8721236, EPI_ISL_8721253, EPI_ISL_8721260, EPI_ISL_8721261, EPI_ISL_8721271, EPI_ISL_8721272, EPI_ISL_8721278, EPI_ISL_8721304, EPI_ISL_8721356, EPI_ISL_8721369, EPI_ISL_8721436, EPI_ISL_8721437, EPI_ISL_8721449, EPI_ISL_8721468, EPI_ISL_8721469, EPI_ISL_8721470, EPI_ISL_8721482, EPI_ISL_8721498, EPI_ISL_8721499, EPI_ISL_8721515, EPI_ISL_8721533, EPI_ISL_8721546, EPI_ISL_8721547, EPI_ISL_8721548, EPI_ISL_8721574, EPI_ISL_8721595, EPI_ISL_8721614, EPI_ISL_8721615, EPI_ISL_8721646, EPI_ISL_8721684, EPI_ISL_8721731, EPI_ISL_8721755, EPI_ISL_8721756, EPI_ISL_8721777, EPI_ISL_8721778, EPI_ISL_8721826, EPI_ISL_8721904, EPI_ISL_8721905, EPI_ISL_8721906, EPI_ISL_8721947, EPI_ISL_8721948, EPI_ISL_8721973			
see above	FACULDADE DE MEDICINA ABC	Instituto Butantan	Antonio Jorge Martins; Claudia Renata dos Santos Barros; David Schlesinger; Debora Botequio Moretti; Dimas Tadeu Covas; Elaine Cristina Marqueze; Elaine Vieira Santos; Evandra Strazza Rodrigues; Heidge Fukumasu; Jayme Augusto de Souza-Neto; José Salvatore Leister Patané; Luiz Alcantara; Luiz Lehmann Coutinho; Maria Carolina Elias; Maurício Lacerda Nogueira; Rafael dos Santos Bezerra; Raul Machado Neto; Rejane Maria Tommasini Grotto; Ricardo Haddad; Sandra Coccuzzo Sampaio Vessoni; Simone Kashima; Svetoslav Nanev Slavov; Vincent Louis Viala
EPI_ISL_8149393, EPI_ISL_8149425, EPI_ISL_8149461	FRANCA - PRONTO SOCORRO DE REFERENCIA DR ALVARO AZZUZ	Instituto Butantan	Antonio Jorge Martins; Claudia Renata dos Santos Barros; David Schlesinger; Debora Botequio Moretti; Dimas Tadeu Covas; Elaine Cristina Marqueze; Elaine Vieira Santos; Evandra Strazza Rodrigues; Heidge Fukumasu; Jayme Augusto de Souza-Neto; José Salvatore Leister Patané; Luiz Alcantara; Luiz Lehmann Coutinho; Maria Carolina Elias; Maurício Lacerda Nogueira; Rafael dos Santos Bezerra; Raul Machado Neto; Rejane Maria Tommasini Grotto; Ricardo Haddad; Sandra Coccuzzo Sampaio Vessoni; Simone Kashima; Svetoslav Nanev Slavov; Vincent Louis Viala
EPI_ISL_8353529, EPI_ISL_8353530, EPI_ISL_8353531, EPI_ISL_8353559, EPI_ISL_8353560, EPI_ISL_8353561, EPI_ISL_8353562, EPI_ISL_8353563, EPI_ISL_8353589, EPI_ISL_8353590, EPI_ISL_8353591, EPI_ISL_8353592, EPI_ISL_8353593, EPI_ISL_8353610, EPI_ISL_8353611			
see above	FUNDACAO BUTANTAN	Instituto Butantan	Antonio Jorge Martins; Claudia Renata dos Santos Barros; David Schlesinger; Debora Botequio Moretti; Dimas Tadeu Covas; Elaine Cristina Marqueze; Elaine Vieira Santos; Evandra Strazza Rodrigues; Heidge Fukumasu; Jayme Augusto de Souza-Neto; José Salvatore Leister Patané; Luiz Alcantara; Luiz Lehmann Coutinho; Maria Carolina Elias; Maurício Lacerda Nogueira; Rafael dos Santos Bezerra; Raul Machado Neto; Rejane Maria Tommasini Grotto; Ricardo Haddad; Sandra Coccuzzo Sampaio Vessoni; Simone Kashima; Svetoslav Nanev Slavov; Vincent Louis Viala
EPI_ISL_7795619, EPI_ISL_7795645, EPI_ISL_7795674, EPI_ISL_8012387, EPI_ISL_8012388, EPI_ISL_8012390, EPI_ISL_8012391, EPI_ISL_8012392, EPI_ISL_8012393, EPI_ISL_8012394, EPI_ISL_8012395, EPI_ISL_8012396, EPI_ISL_8012397			
see above	Fundação Ezequiel Dias (FUNED)	Fundação Ezequiel Dias (FUNED)	Adriana Ribeiro; Andre Leal; Emerson de Castro; Felipe Iani; Glauco Carvalho.; Joilson Xavier; Luiz Alcantara; Marta Giovanetti; Natalia Guimaraes; Natália Guimarães; Talita Adelino; Vagner Fonseca
EPI_ISL_6913917	Grupo CR Diagnosticos	Instituto Adolfo Lutz Strategic Laboratory	Claudio Tavares Sacchi; Karoline Rodrigues Campos
EPI_ISL_8621464	H J M A HOSPITAL JOSE MARTINIANO DE ALENCAR	Analytical Competence Molecular Epidemiology Lab/ACME, Oswaldo Cruz Foundation, Ceara (FIOCRUZ CE)	Carlos Leonardo de Aragao Araujo; Cleber Furtado Aksenen; Fabio Miyajima; Fernando Braga Stehling; Jamille Maria Mendes Bezerra; Joaquim Cesar do Nascimento Sousa Junior; Pedro Miguel Carneiro Jeronimo; Suzana Porto Almeida & Igor Oliveira Duarte on behalf of COVID-19 FIOCRUZ Genomic Network; Thais Ferreira de Oliveira; Thais de Oliveira Costa; Ticiane Cavalcante de Souza; Veridiana Pessoa Miyajima
EPI_ISL_8184729, EPI_ISL_8184730, EPI_ISL_8621441, EPI_ISL_8621449, EPI_ISL_8621450, EPI_ISL_8621455, EPI_ISL_8621465, EPI_ISL_8621475, EPI_ISL_8621479, EPI_ISL_8621483, EPI_ISL_8621484, EPI_ISL_8621485, EPI_ISL_8621490, EPI_ISL_8621491, EPI_ISL_8621495, EPI_ISL_8621498, EPI_ISL_8621499, EPI_ISL_8621502, EPI_ISL_8621506, EPI_ISL_8621514, EPI_ISL_8621521, EPI_ISL_8621523, EPI_ISL_8621532, EPI_ISL_8621534, EPI_ISL_8621535, EPI_ISL_8621538			
see above	HEMOCE CENTRO DE HEMATOLOGIA E HEMOTERAPIA DO CEARA	Analytical Competence Molecular Epidemiology Lab/ACME, Oswaldo Cruz Foundation, Ceara (FIOCRUZ CE)	Carlos Leonardo de Aragao Araujo; Cleber Furtado Aksenen; Fabio Miyajima; Fernando Braga Stehling; Jamille Maria Mendes Bezerra; Joaquim Cesar do Nascimento Sousa Junior; Pedro Miguel Carneiro Jeronimo; Suzana Porto Almeida & Igor Oliveira Duarte on behalf of COVID-19 FIOCRUZ Genomic Network; Thais Ferreira de Oliveira; Thais de Oliveira Costa; Ticiane Cavalcante de Souza; Veridiana Pessoa Miyajima
EPI_ISL_8184732, EPI_ISL_8184737, EPI_ISL_8184739, EPI_ISL_8621456, EPI_ISL_8621477, EPI_ISL_8621486	HGCC HOSPITAL GERAL DR CESAR CALS	Analytical Competence Molecular Epidemiology Lab/ACME, Oswaldo Cruz Foundation, Ceara (FIOCRUZ CE)	Carlos Leonardo de Aragao Araujo; Cleber Furtado Aksenen; Fabio Miyajima; Fernando Braga Stehling; Jamille Maria Mendes Bezerra; Joaquim Cesar do Nascimento Sousa Junior; Pedro Miguel Carneiro Jeronimo; Suzana Porto Almeida & Igor Oliveira Duarte on behalf of COVID-19 FIOCRUZ Genomic Network; Thais Ferreira de Oliveira; Thais de Oliveira Costa; Ticiane Cavalcante de Souza; Veridiana Pessoa Miyajima
EPI_ISL_8621472, EPI_ISL_8621473, EPI_ISL_8621476, EPI_ISL_8621528, EPI_ISL_8621529, EPI_ISL_8621530	HIAS HOSPITAL INFANTIL ALBERT SABIN	Analytical Competence Molecular Epidemiology Lab/ACME, Oswaldo Cruz Foundation, Ceara (FIOCRUZ CE)	Carlos Leonardo de Aragao Araujo; Cleber Furtado Aksenen; Fabio Miyajima; Fernando Braga Stehling; Jamille Maria Mendes Bezerra; Joaquim Cesar do Nascimento Sousa Junior; Pedro Miguel Carneiro Jeronimo; Suzana Porto Almeida & Igor Oliveira Duarte on behalf of COVID-19 FIOCRUZ Genomic Network; Thais Ferreira de Oliveira; Thais de Oliveira Costa; Ticiane Cavalcante de Souza; Veridiana Pessoa Miyajima
EPI_ISL_8621442, EPI_ISL_8621451, EPI_ISL_8621452, EPI_ISL_8621454, EPI_ISL_8621467, EPI_ISL_8621468, EPI_ISL_8621469, EPI_ISL_8621470, EPI_ISL_8621471, EPI_ISL_8621474, EPI_ISL_8621481, EPI_ISL_8621482, EPI_ISL_8621492, EPI_ISL_8621501, EPI_ISL_8621508, EPI_ISL_8621524, EPI_ISL_8621525, EPI_ISL_8621526, EPI_ISL_8621539			
see above	HM HOSPITAL DE MESSEJANA DR CARLOS ALBERTO STUDART GOMES	Analytical Competence Molecular Epidemiology Lab/ACME, Oswaldo Cruz Foundation, Ceara (FIOCRUZ CE)	Carlos Leonardo de Aragao Araujo; Cleber Furtado Aksenen; Fabio Miyajima; Fernando Braga Stehling; Jamille Maria Mendes Bezerra; Joaquim Cesar do Nascimento Sousa Junior; Pedro Miguel Carneiro Jeronimo; Suzana Porto Almeida & Igor Oliveira Duarte on behalf of COVID-19 FIOCRUZ Genomic Network; Thais Ferreira de Oliveira; Thais de Oliveira Costa; Ticiane Cavalcante de Souza; Veridiana Pessoa Miyajima
EPI_ISL_8721427, EPI_ISL_8721740, EPI_ISL_8721911	HOSP E MATERNIDADE MUNICIPAL N SRA MONTE SERRAT	Instituto Butantan	Antonio Jorge Martins; Claudia Renata dos Santos Barros; David Schlesinger; Debora Botequio Moretti; Dimas Tadeu Covas; Elaine Cristina Marqueze; Elaine Vieira Santos; Evandra Strazza Rodrigues; Heidge Fukumasu; Jayme Augusto de Souza-Neto; José Salvatore Leister Patané; Luiz Alcantara; Luiz Lehmann Coutinho; Maria Carolina Elias; Maurício Lacerda Nogueira; Rafael dos Santos Bezerra; Raul Machado Neto; Rejane Maria Tommasini Grotto; Ricardo Haddad; Sandra Coccuzzo Sampaio Vessoni; Simone Kashima; Svetoslav Nanev Slavov; Vincent Louis Viala
EPI_ISL_8184831, EPI_ISL_8721270, EPI_ISL_8721513, EPI_ISL_8721658, EPI_ISL_8721659	HOSP MUN DE MOGI DAS CRUZES PREF WALDEMAR COSTA FILHO	Instituto Butantan	Antonio Jorge Martins; Claudia Renata dos Santos Barros; David Schlesinger; Debora Botequio Moretti; Dimas Tadeu Covas; Elaine Cristina Marqueze; Elaine Vieira Santos; Evandra Strazza Rodrigues; Heidge Fukumasu; Jayme Augusto de Souza-Neto; José Salvatore Leister Patané; Luiz Alcantara; Luiz Lehmann Coutinho; Maria Carolina Elias; Maurício Lacerda Nogueira; Rafael dos Santos Bezerra; Raul Machado Neto; Rejane Maria Tommasini Grotto; Ricardo Haddad; Sandra Coccuzzo Sampaio Vessoni; Simone Kashima; Svetoslav Nanev Slavov; Vincent Louis Viala
EPI_ISL_8721217	HOSP MUN ERMELINO MATARAZZO ALIPIO CORREA NETTO	Instituto Butantan	Antonio Jorge Martins; Claudia Renata dos Santos Barros; David Schlesinger; Debora Botequio Moretti; Dimas Tadeu Covas; Elaine Cristina Marqueze; Elaine Vieira Santos; Evandra Strazza Rodrigues; Heidge Fukumasu; Jayme Augusto de Souza-Neto; José Salvatore Leister Patané; Luiz Alcantara; Luiz Lehmann Coutinho; Maria Carolina Elias; Maurício Lacerda Nogueira; Rafael dos Santos Bezerra; Raul Machado Neto; Rejane Maria Tommasini Grotto; Ricardo Haddad; Sandra Coccuzzo Sampaio Vessoni; Simone Kashima; Svetoslav Nanev Slavov; Vincent Louis Viala
EPI_ISL_8721322	HOSPITAL DA COSTA SUL DE SAO SEBASTIAO	Instituto Butantan	Antonio Jorge Martins; Claudia Renata dos Santos Barros; David Schlesinger; Debora Botequio Moretti; Dimas Tadeu Covas; Elaine Cristina Marqueze; Elaine Vieira Santos; Evandra Strazza Rodrigues; Heidge Fukumasu; Jayme Augusto de Souza-Neto; José Salvatore Leister Patané; Luiz Alcantara; Luiz Lehmann Coutinho; Maria Carolina Elias; Maurício Lacerda Nogueira; Rafael dos Santos Bezerra; Raul Machado Neto; Rejane Maria Tommasini Grotto; Ricardo Haddad; Sandra Coccuzzo Sampaio Vessoni; Simone Kashima; Svetoslav Nanev Slavov; Vincent Louis Viala
EPI_ISL_8621507	HOSPITAL DE SAUDE MENTAL DE MESSEJANA	Analytical Competence Molecular Epidemiology Lab/ACME, Oswaldo Cruz Foundation, Ceara (FIOCRUZ CE)	Carlos Leonardo de Aragao Araujo; Cleber Furtado Aksenen; Fabio Miyajima; Fernando Braga Stehling; Jamille Maria Mendes Bezerra; Joaquim Cesar do Nascimento Sousa Junior; Pedro Miguel Carneiro Jeronimo; Suzana Porto Almeida & Igor Oliveira Duarte on behalf of COVID-19 FIOCRUZ Genomic Network; Thais Ferreira de Oliveira; Thais de Oliveira Costa; Ticiane Cavalcante de Souza; Veridiana Pessoa Miyajima
EPI_ISL_8184830	HOSPITAL DOS FORNECEDORES DE CANA DE PIRACICABA	Instituto Butantan	Antonio Jorge Martins; Claudia Renata dos Santos Barros; David Schlesinger; Debora Botequio Moretti; Dimas Tadeu Covas; Elaine Cristina Marqueze; Elaine Vieira Santos; Evandra Strazza Rodrigues; Heidge Fukumasu; Jayme Augusto de Souza-Neto; José Salvatore Leister Patané; Luiz Alcantara; Luiz Lehmann Coutinho; Maria Carolina Elias; Maurício Lacerda Nogueira; Rafael dos Santos Bezerra; Raul Machado Neto; Rejane Maria Tommasini Grotto; Ricardo Haddad; Sandra Coccuzzo Sampaio Vessoni; Simone Kashima; Svetoslav Nanev Slavov; Vincent Louis Viala
EPI_ISL_8621460, EPI_ISL_8621461, EPI_ISL_8621462, EPI_ISL_8621463, EPI_ISL_8621500, EPI_ISL_8621515, EPI_ISL_8621516, EPI_ISL_8621531			
see above	HOSPITAL E MATERNIDADE DRA ZILDA ARNS NEUMANN	Analytical Competence Molecular Epidemiology Lab/ACME, Oswaldo Cruz Foundation, Ceara (FIOCRUZ CE)	Carlos Leonardo de Aragao Araujo; Cleber Furtado Aksenen; Fabio Miyajima; Fernando Braga Stehling; Jamille Maria Mendes Bezerra; Joaquim Cesar do Nascimento Sousa Junior; Pedro Miguel Carneiro Jeronimo; Suzana Porto Almeida & Igor Oliveira Duarte on behalf of COVID-19 FIOCRUZ Genomic Network; Thais Ferreira de Oliveira; Thais de Oliveira Costa; Ticiane Cavalcante de Souza; Veridiana Pessoa Miyajima
EPI_ISL_8184738, EPI_ISL_8621466, EPI_ISL_8621533, EPI_ISL_8621537	HOSPITAL ESTADUAL LEONARDO DA VINCI	Analytical Competence Molecular Epidemiology Lab/ACME, Oswaldo Cruz Foundation, Ceara (FIOCRUZ CE)	Carlos Leonardo de Aragao Araujo; Cleber Furtado Aksenen; Fabio Miyajima; Fernando Braga Stehling; Igor Oliveira Duarte & Nicole Silva França on behalf of COVID-19 FIOCRUZ Genomic Network; Jamille Maria Mendes Bezerra; Joaquim Cesar do Nascimento Sousa Junior; Pedro Miguel Carneiro Jeronimo; Suzana Porto Almeida; Suzana Porto Almeida & Igor Oliveira Duarte on behalf of COVID-19 FIOCRUZ Genomic Network; Thais Ferreira de Oliveira; Thais de Oliveira Costa; Ticiane Cavalcante de Souza; Veridiana Pessoa Miyajima
EPI_ISL_8721507	HOSPITAL GERAL DE ITAPEVI	Instituto Butantan	Antonio Jorge Martins; Claudia Renata dos Santos Barros; David Schlesinger; Debora Botequio Moretti; Dimas Tadeu Covas; Elaine Cristina Marqueze; Elaine Vieira Santos; Evandra Strazza Rodrigues; Heidge Fukumasu; Jayme Augusto de Souza-Neto; José Salvatore Leister Patané; Luiz Alcantara; Luiz Lehmann Coutinho; Maria Carolina Elias; Maurício Lacerda Nogueira; Rafael dos Santos Bezerra; Raul Machado Neto; Rejane Maria Tommasini Grotto; Ricardo Haddad; Sandra Coccuzzo Sampaio Vessoni; Simone Kashima; Svetoslav Nanev Slavov; Vincent Louis Viala
EPI_ISL_8721463	HOSPITAL GERAL JESUS TEIXEIRA DA COSTA GUAIANASES SAO PAULO	Instituto Butantan	Antonio Jorge Martins; Claudia Renata dos Santos Barros; David Schlesinger; Debora Botequio Moretti; Dimas Tadeu Covas; Elaine Cristina Marqueze; Elaine Vieira Santos; Evandra Strazza Rodrigues; Heidge Fukumasu; Jayme Augusto de Souza-Neto; José Salvatore Leister Patané; Luiz Alcantara; Luiz Lehmann Coutinho; Maria Carolina Elias; Maurício Lacerda Nogueira; Rafael dos Santos Bezerra; Raul Machado Neto; Rejane Maria Tommasini Grotto; Ricardo Haddad; Sandra Coccuzzo Sampaio Vessoni; Simone Kashima; Svetoslav Nanev Slavov; Vincent Louis Viala
EPI_ISL_8353554	HOSPITAL GERAL SANTA MARCELINA DE ITAIM PAULISTA SAO PAULO	Instituto Butantan	Antonio Jorge Martins; Claudia Renata dos Santos Barros; David Schlesinger; Debora Botequio Moretti; Dimas Tadeu Covas; Elaine Cristina Marqueze; Elaine Vieira Santos; Evandra Strazza Rodrigues; Heidge Fukumasu; Jayme Augusto de Souza-Neto; José Salvatore Leister Patané; Luiz Alcantara; Luiz Lehmann Coutinho; Maria Carolina Elias; Maurício Lacerda Nogueira; Rafael dos Santos Bezerra; Raul Machado Neto; Rejane Maria Tommasini Grotto; Ricardo Haddad; Sandra Coccuzzo Sampaio Vessoni; Simone Kashima; Svetoslav Nanev Slavov; Vincent Louis Viala
EPI_ISL_8465112	HOSPITAL MUNICIPAL DE CONCHAS	Instituto Butantan	Antonio Jorge Martins; Claudia Renata dos Santos Barros; David Schlesinger; Debora Botequio Moretti; Dimas Tadeu Covas; Elaine Cristina Marqueze; Elaine Vieira Santos; Evandra Strazza Rodrigues; Heidge Fukumasu; Jayme Augusto de Souza-Neto; José Salvatore Leister Patané; Luiz Alcantara; Luiz Lehmann Coutinho; Maria Carolina Elias; Maurício Lacerda Nogueira; Rafael dos Santos Bezerra; Raul Machado Neto; Rejane Maria Tommasini Grotto; Ricardo Haddad; Sandra Coccuzzo Sampaio Vessoni; Simone Kashima; Svetoslav Nanev Slavov; Vincent Louis Viala
EPI_ISL_8353549, EPI_ISL_8353550, EPI_ISL_8353555, EPI_ISL_8353556, EPI_ISL_8353569, EPI_ISL_8353570, EPI_ISL_8353575, EPI_ISL_8353576, EPI_ISL_8721226, EPI_ISL_8721243, EPI_ISL_8721249, EPI_ISL_8721291, EPI_ISL_8721299, EPI_ISL_8721319, EPI_ISL_8721337, EPI_ISL_8721338, EPI_ISL_8721354, EPI_ISL_8721375, EPI_ISL_8721376, EPI_ISL_8721383, EPI_ISL_8721410, EPI_ISL_8721416, EPI_ISL_8721426, EPI_ISL_8721444, EPI_ISL_8721489, EPI_ISL_8721508, EPI_ISL_8721520, EPI_ISL_8721522, EPI_ISL_8721523, EPI_ISL_8721537, EPI_ISL_8721540, EPI_ISL_8721553, EPI_ISL_8721554, EPI_ISL_8721555, EPI_ISL_8721565, EPI_ISL_8721586, EPI_ISL_8721625, EPI_ISL_8721637, EPI_ISL_8721669, EPI_ISL_8721670, EPI_ISL_8721712, EPI_ISL_8721747, EPI_ISL_8721763, EPI_ISL_8721787, EPI_ISL_8721788, EPI_ISL_8721789, EPI_ISL_8721839, EPI_ISL_8721861, EPI_ISL_8721887, EPI_ISL_8721892, EPI_ISL_8721971, EPI_ISL_8721995, EPI_ISL_8721999			
see above	HOSPITAL MUNICIPAL DE ILHABELA GOV MARIO COVAS JR	Instituto Butantan	Antonio Jorge Martins; Claudia Renata dos Santos Barros; David Schlesinger; Debora Botequio Moretti; Dimas Tadeu Covas; Elaine Cristina Marqueze; Elaine Vieira Santos; Evandra Strazza Rodrigues; Heidge Fukumasu; Jayme Augusto de Souza-Neto; José Salvatore Leister Patané; Luiz Alcantara; Luiz Lehmann Coutinho; Maria Carolina Elias; Maurício Lacerda Nogueira; Rafael dos Santos Bezerra; Raul Machado Neto; Rejane Maria Tommasini Grotto; Ricardo Haddad; Sandra Coccuzzo Sampaio Vessoni; Simone Kashima; Svetoslav Nanev Slavov; Vincent Louis

			Viala
EPI_ISL_8721455, EPI_ISL_8721608, EPI_ISL_8721677	HOSPITAL MUNICIPAL DE ITAPIRA	Instituto Butantan	Antonio Jorge Martins; Claudia Renata dos Santos Barros; David Schlesinger; Debora Botequio Moretti; Dimas Tadeu Covas; Elaine Cristina Marqueze; Elaine Vieira Santos; Evandra Strazza Rodrigues; Heidge Fukumasu; Jayme Augusto de Souza-Neto; José Salvatore Leister Patané; Luiz Alcantara; Luiz Lehmann Coutinho; Maria Carolina Elias; Maurício Lacerda Nogueira; Rafael dos Santos Bezerra; Raul Machado Neto; Rejane Maria Tommasini Grotto; Ricardo Haddad; Sandra Coccuzzo Sampaio Vessoni; Simone Kashima; Svetoslav Nanev Slavov; Vincent Louis Viala
EPI_ISL_7649952, EPI_ISL_8184833, EPI_ISL_8721252, EPI_ISL_8721567, EPI_ISL_8721606, EPI_ISL_8721655, EPI_ISL_8721690, EPI_ISL_8721697, EPI_ISL_8721765, EPI_ISL_8721793, EPI_ISL_8721888			
see above	HOSPITAL MUNICIPAL DR JOSE DE CARVALHO FLORENCE	Instituto Butantan	Antonio Jorge Martins; Claudia Renata dos Santos Barros; David Schlesinger; Debora Botequio Moretti; Dimas Tadeu Covas; Elaine Cristina Marqueze; Elaine Vieira Santos; Evandra Strazza Rodrigues; Heidge Fukumasu; Jayme Augusto de Souza-Neto; José Salvatore Leister Patané; Luiz Alcantara; Luiz Lehmann Coutinho; Maria Carolina Elias; Maurício Lacerda Nogueira; Rafael dos Santos Bezerra; Raul Machado Neto; Rejane Maria Tommasini Grotto; Ricardo Haddad; Sandra Coccuzzo Sampaio Vessoni; Simone Kashima; Svetoslav Nanev Slavov; Vincent Louis Viala
EPI_ISL_8721256, EPI_ISL_8721429, EPI_ISL_8721511, EPI_ISL_8721602, EPI_ISL_8721698, EPI_ISL_8721742, EPI_ISL_8721744, EPI_ISL_8721748, EPI_ISL_8721767, EPI_ISL_8721768, EPI_ISL_8721792, EPI_ISL_8721837, EPI_ISL_8721840, EPI_ISL_8721863, EPI_ISL_8721939, EPI_ISL_8721983			
see above	HOSPITAL MUNICIPAL DR MARIO GATTI CAMPINAS	Instituto Butantan	Antonio Jorge Martins; Claudia Renata dos Santos Barros; David Schlesinger; Debora Botequio Moretti; Dimas Tadeu Covas; Elaine Cristina Marqueze; Elaine Vieira Santos; Evandra Strazza Rodrigues; Heidge Fukumasu; Jayme Augusto de Souza-Neto; José Salvatore Leister Patané; Luiz Alcantara; Luiz Lehmann Coutinho; Maria Carolina Elias; Maurício Lacerda Nogueira; Rafael dos Santos Bezerra; Raul Machado Neto; Rejane Maria Tommasini Grotto; Ricardo Haddad; Sandra Coccuzzo Sampaio Vessoni; Simone Kashima; Svetoslav Nanev Slavov; Vincent Louis Viala
EPI_ISL_8880834	HOSPITAL NEREU RAMOS - FLORIANOPOLIS	Laboratório de Bioinformática - Universidade Federal de Santa Catarina	Alexandra Crispim Boing; Ariane Nicaretta Amorim; Darcita Buerger Rovaris; Dayane Azevedo Padilha; Doris Sobral Marques Souza; Eric Kazuo Kawagoe; Fernanda Rosene Melo; Fernando Hartmann Barazzetti; Gislaine Fongaro; Glauber Wagner; João Augusto Brancher Fuck; Juliana Righetto Moser; Luiz Felipe Valter de Oliveira; Marcos André Schörner; Maria Luiza Bazzo; Marlei Pickler Debiasi dos Anjos; Milene Moehr de Moraes; Sandra Bianchini Fernandes; Vilmar Benetti Filho; on behalf of the Santa Catarina COVID-19 Genomic Surveillance Network (Genoma COVID SC)
EPI_ISL_8184733, EPI_ISL_8184734, EPI_ISL_8184735, EPI_ISL_8621493, EPI_ISL_8621494	HOSPITAL OTOCLINICA	Analytical Competence Molecular Epidemiology Lab/ACME, Oswaldo Cruz Foundation, Ceara (FIOCRUZ CE)	Carlos Leonardo de Aragao Araujo; Cleber Furtado Aksenen; Fabio Miyajima; Fernando Braga Stehling; Igor Oliveira Duarte & Nicole Silva França on behalf of COVID-19 FIOCRUZ Genomic Network; Igor Oliveira Duarte on behalf & Nicole Silva França of COVID-19 FIOCRUZ Genomic Network; Jamille Maria Mendes Bezerra; Joaquim Cesar do Nascimento Sousa Junior; Pedro Miguel Carneiro Jeronimo; Suzana Porto Almeida; Thais Ferreira de Oliveira; Thais de Oliveira Costa; Ticiane Cavalcante de Souza; Veridiana Pessoa Miyajima
EPI_ISL_8721307	HOSPITAL REGIONAL DE ITAPETININGA	Instituto Butantan	Antonio Jorge Martins; Claudia Renata dos Santos Barros; David Schlesinger; Debora Botequio Moretti; Dimas Tadeu Covas; Elaine Cristina Marqueze; Elaine Vieira Santos; Evandra Strazza Rodrigues; Heidge Fukumasu; Jayme Augusto de Souza-Neto; José Salvatore Leister Patané; Luiz Alcantara; Luiz Lehmann Coutinho; Maria Carolina Elias; Maurício Lacerda Nogueira; Rafael dos Santos Bezerra; Raul Machado Neto; Rejane Maria Tommasini Grotto; Ricardo Haddad; Sandra Coccuzzo Sampaio Vessoni; Simone Kashima; Svetoslav Nanev Slavov; Vincent Louis Viala
EPI_ISL_8721266	HOSPITAL SANTO ANTONIO DE JUQUIA JUQUIA	Instituto Butantan	Antonio Jorge Martins; Claudia Renata dos Santos Barros; David Schlesinger; Debora Botequio Moretti; Dimas Tadeu Covas; Elaine Cristina Marqueze; Elaine Vieira Santos; Evandra Strazza Rodrigues; Heidge Fukumasu; Jayme Augusto de Souza-Neto; José Salvatore Leister Patané; Luiz Alcantara; Luiz Lehmann Coutinho; Maria Carolina Elias; Maurício Lacerda Nogueira; Rafael dos Santos Bezerra; Raul Machado Neto; Rejane Maria Tommasini Grotto; Ricardo Haddad; Sandra Coccuzzo Sampaio Vessoni; Simone Kashima; Svetoslav Nanev Slavov; Vincent Louis Viala
EPI_ISL_8621443, EPI_ISL_8621444, EPI_ISL_8621445, EPI_ISL_8621446, EPI_ISL_8621447, EPI_ISL_8621448, EPI_ISL_8621457, EPI_ISL_8621458, EPI_ISL_8621459, EPI_ISL_8621478, EPI_ISL_8621480, EPI_ISL_8621487, EPI_ISL_8621488, EPI_ISL_8621489, EPI_ISL_8621496, EPI_ISL_8621497, EPI_ISL_8621503, EPI_ISL_8621510, EPI_ISL_8621511, EPI_ISL_8621512, EPI_ISL_8621513, EPI_ISL_8621517, EPI_ISL_8621518, EPI_ISL_8621519, EPI_ISL_8621520, EPI_ISL_8621522			
see above	HOSPITAL SAO JOSE DE DOENCAS INFECCIOSAS	Analytical Competence Molecular Epidemiology Lab/ACME, Oswaldo Cruz Foundation, Ceara (FIOCRUZ CE)	Carlos Leonardo de Aragao Araujo; Cleber Furtado Aksenen; Fabio Miyajima; Fernando Braga Stehling; Jamille Maria Mendes Bezerra; Joaquim Cesar do Nascimento Sousa Junior; Pedro Miguel Carneiro Jeronimo; Suzana Porto Almeida & Igor Oliveira Duarte on behalf of COVID-19 FIOCRUZ Genomic Network; Thais Ferreira de Oliveira; Thais de Oliveira Costa; Ticiane Cavalcante de Souza; Veridiana Pessoa Miyajima
EPI_ISL_8621453, EPI_ISL_8621504, EPI_ISL_8621505, EPI_ISL_8621527, EPI_ISL_8621536	HOSPITAL UNIVERSITARIO WALTER CANTIDIO	Analytical Competence Molecular Epidemiology Lab/ACME, Oswaldo Cruz Foundation, Ceara (FIOCRUZ CE)	Carlos Leonardo de Aragao Araujo; Cleber Furtado Aksenen; Fabio Miyajima; Fernando Braga Stehling; Jamille Maria Mendes Bezerra; Joaquim Cesar do Nascimento Sousa Junior; Pedro Miguel Carneiro Jeronimo; Suzana Porto Almeida & Igor Oliveira Duarte on behalf of COVID-19 FIOCRUZ Genomic Network; Thais Ferreira de Oliveira; Thais de Oliveira Costa; Ticiane Cavalcante de Souza; Veridiana Pessoa Miyajima
EPI_ISL_3368634, EPI_ISL_3368635	IEC- Instituto Evandro Chagas	ITV-Vale Institute of Technology	Amanda Vidal; Guilherme Oliveira; Mirleide Cordeiro dos Santos; Tatianne Costa Negri
EPI_ISL_1213173, EPI_ISL_1213175, EPI_ISL_1213177, EPI_ISL_1213178, EPI_ISL_1213180, EPI_ISL_1213182, EPI_ISL_1213183, EPI_ISL_1213185, EPI_ISL_1213187, EPI_ISL_1213192, EPI_ISL_1213194, EPI_ISL_1213196, EPI_ISL_1213197, EPI_ISL_1213199, EPI_ISL_1213209, EPI_ISL_1213275, EPI_ISL_1213277, EPI_ISL_1213279, EPI_ISL_1213281, EPI_ISL_1213282, EPI_ISL_1213284, EPI_ISL_1213286, EPI_ISL_1213288, EPI_ISL_1213289, EPI_ISL_1213291, EPI_ISL_1213293, EPI_ISL_1213315, EPI_ISL_1213317, EPI_ISL_1213319, EPI_ISL_1213320, EPI_ISL_1213322, EPI_ISL_1213329, EPI_ISL_1213345, EPI_ISL_1213346, EPI_ISL_1213348, EPI_ISL_1213350, EPI_ISL_1213352, EPI_ISL_1213353, EPI_ISL_1213355, EPI_ISL_1213357, EPI_ISL_1213358, EPI_ISL_1213360, EPI_ISL_1213362, EPI_ISL_1213364			
see above	IMT-UFRN/RN	Bioinformatics Laboratory / LNCC	Alessandra P Lamarca; Alexandra L Gerber; Ana Paula Melo Mariano; Ana Paula de C Guimarães; Ana Tereza R Vasconcelos; Angela Maria Guimarães Santos; Bianca Mendes Maciel; Danielle Angst Secco; Eduardo Sérgio Soares Sousa; Eloiza Helena Campana; Francisco Paulo Freire Neto; George Rego Albuquerque; Kátia Castanho Scortecci; Lucymara Fassarella Agnez Lima; Luiz G P de Almeida; Luís Cristóvão Porto; Otavio J. Brustolini; Paulo Ricardo Nascimento; Ronaldo da Silva Francisco Jr; Sandra Rocha Gadelha; Selma Maria Bezerra Jeronimo; Vinícius Pietta Perez
EPI_ISL_8184801	INSEDE	Instituto Butantan	Antonio Jorge Martins; Claudia Renata dos Santos Barros; David Schlesinger; Debora Botequio Moretti; Dimas Tadeu Covas; Elaine Cristina Marqueze; Elaine Vieira Santos; Evandra Strazza Rodrigues; Heidge Fukumasu; Jayme Augusto de Souza-Neto; José Salvatore Leister Patané; Luiz Alcantara; Luiz Lehmann Coutinho; Maria Carolina Elias; Maurício Lacerda Nogueira; Rafael dos Santos Bezerra; Raul Machado Neto; Rejane Maria Tommasini Grotto; Ricardo Haddad; Sandra Coccuzzo Sampaio Vessoni; Simone Kashima; Svetoslav Nanev Slavov; Vincent Louis Viala
EPI_ISL_7883751, EPI_ISL_7883752, EPI_ISL_8184778, EPI_ISL_8184781, EPI_ISL_8184782, EPI_ISL_8184783, EPI_ISL_8184784, EPI_ISL_8184785, EPI_ISL_8184786, EPI_ISL_8184787, EPI_ISL_8184788, EPI_ISL_8184789, EPI_ISL_8184790, EPI_ISL_8184791, EPI_ISL_8184792, EPI_ISL_8184793, EPI_ISL_8184794, EPI_ISL_8184795, EPI_ISL_8184796, EPI_ISL_8184797, EPI_ISL_8353523, EPI_ISL_8353535, EPI_ISL_8353536, EPI_ISL_8353567, EPI_ISL_8353568, EPI_ISL_8353597, EPI_ISL_8353598, EPI_ISL_8353599, EPI_ISL_8353600, EPI_ISL_8353601, EPI_ISL_8353602, EPI_ISL_8353603, EPI_ISL_8353604, EPI_ISL_8353605, EPI_ISL_8687817, EPI_ISL_8687818, EPI_ISL_8687820, EPI_ISL_8687821, EPI_ISL_8687822, EPI_ISL_8687823, EPI_ISL_8687824, EPI_ISL_8687825, EPI_ISL_8687826, EPI_ISL_8687827, EPI_ISL_8687828, EPI_ISL_8687829, EPI_ISL_8687830, EPI_ISL_8687831, EPI_ISL_8687832, EPI_ISL_8687833, EPI_ISL_8687834, EPI_ISL_8687835, EPI_ISL_8687836, EPI_ISL_8687837, EPI_ISL_8687838, EPI_ISL_8687839, EPI_ISL_8687840, EPI_ISL_8687841, EPI_ISL_8687842, EPI_ISL_8687843, EPI_ISL_8687844, EPI_ISL_8687845, EPI_ISL_8687848, EPI_ISL_8687849, EPI_ISL_8687850, EPI_ISL_8687851, EPI_ISL_8687852, EPI_ISL_8687853, EPI_ISL_8687854, EPI_ISL_8687855, EPI_ISL_8687856, EPI_ISL_8687857, EPI_ISL_8687858, EPI_ISL_8687859, EPI_ISL_8687860, EPI_ISL_8687861, EPI_ISL_8687862, EPI_ISL_8687863, EPI_ISL_8687864, EPI_ISL_8687865, EPI_ISL_8687866, EPI_ISL_8687867, EPI_ISL_8687868, EPI_ISL_8687869, EPI_ISL_8687870, EPI_ISL_8687871, EPI_ISL_8687872, EPI_ISL_8687873, EPI_ISL_8687874, EPI_ISL_8687876, EPI_ISL_8687877, EPI_ISL_8687878, EPI_ISL_8687879, EPI_ISL_8687880, EPI_ISL_8687881, EPI_ISL_8687882, EPI_ISL_8687883, EPI_ISL_8687884, EPI_ISL_8687885			
see above	INSIDE	Instituto Butantan	Antonio Jorge Martins; Claudia Renata dos Santos Barros; David Schlesinger; Debora Botequio Moretti; Dimas Tadeu Covas; Elaine Cristina Marqueze; Elaine Vieira Santos; Evandra Strazza Rodrigues; Heidge Fukumasu; Jayme Augusto de Souza-Neto; José Salvatore Leister Patané; Luiz Alcantara; Luiz Lehmann Coutinho; Maria Carolina Elias; Maurício Lacerda Nogueira; Rafael dos Santos Bezerra; Raul Machado Neto; Rejane Maria Tommasini Grotto; Ricardo Haddad; Sandra Coccuzzo Sampaio Vessoni; Simone Kashima; Svetoslav Nanev Slavov; Vincent Louis Viala
EPI_ISL_7779340	INSIDE DIAGNOSTICOS	Instituto Butantan	Antonio Jorge Martins; Claudia Renata dos Santos Barros; David Schlesinger; Debora Botequio Moretti; Dimas Tadeu Covas; Elaine Cristina Marqueze; Elaine Vieira Santos; Evandra Strazza Rodrigues; Heidge Fukumasu; Jayme Augusto de Souza-Neto; José Salvatore Leister Patané; Luiz Alcantara; Luiz Lehmann Coutinho; Maria Carolina Elias; Maurício Lacerda Nogueira; Rafael dos Santos Bezerra; Raul Machado Neto; Rejane Maria Tommasini Grotto; Ricardo Haddad; Sandra Coccuzzo Sampaio Vessoni; Simone Kashima; Svetoslav Nanev Slavov; Vincent Louis Viala
EPI_ISL_8721248, EPI_ISL_8721268, EPI_ISL_8721273, EPI_ISL_8721288, EPI_ISL_8721313, EPI_ISL_8721314, EPI_ISL_8721331, EPI_ISL_8721357, EPI_ISL_8721358, EPI_ISL_8721359, EPI_ISL_8721360, EPI_ISL_8721361, EPI_ISL_8721362, EPI_ISL_8721370, EPI_ISL_8721422, EPI_ISL_8721461, EPI_ISL_8721516, EPI_ISL_8721530, EPI_ISL_8721549, EPI_ISL_8721550, EPI_ISL_8721557, EPI_ISL_8721558, EPI_ISL_8721575, EPI_ISL_8721596, EPI_ISL_8721597, EPI_ISL_8721631, EPI_ISL_8721647, EPI_ISL_8721648, EPI_ISL_8721649, EPI_ISL_8721685, EPI_ISL_8721686, EPI_ISL_8721687, EPI_ISL_8721706, EPI_ISL_8721732, EPI_ISL_8721733, EPI_ISL_8721734, EPI_ISL_8721757, EPI_ISL_8721779, EPI_ISL_8721780, EPI_ISL_8721781, EPI_ISL_8721806, EPI_ISL_8721807, EPI_ISL_8721827, EPI_ISL_8721852, EPI_ISL_8721853, EPI_ISL_8721854, EPI_ISL_8721876, EPI_ISL_8721877, EPI_ISL_8721878, EPI_ISL_8721928, EPI_ISL_8721929, EPI_ISL_8721949, EPI_ISL_8721974, EPI_ISL_8721981, EPI_ISL_8721987			
see above	INSIDE DIAGNÓSTICOS	Instituto Butantan	Antonio Jorge Martins; Claudia Renata dos Santos Barros; David Schlesinger; Debora Botequio Moretti; Dimas Tadeu Covas; Elaine Cristina Marqueze; Elaine Vieira Santos; Evandra Strazza Rodrigues; Heidge Fukumasu; Jayme Augusto de Souza-Neto; José Salvatore Leister Patané; Luiz Alcantara; Luiz Lehmann Coutinho; Maria Carolina Elias; Maurício Lacerda Nogueira; Rafael dos Santos Bezerra; Raul Machado Neto; Rejane Maria Tommasini Grotto; Ricardo Haddad; Sandra Coccuzzo Sampaio Vessoni; Simone Kashima; Svetoslav Nanev Slavov; Vincent Louis Viala
EPI_ISL_8721220, EPI_ISL_8721280, EPI_ISL_8721317, EPI_ISL_8721318, EPI_ISL_8721372, EPI_ISL_8721380, EPI_ISL_8721381, EPI_ISL_8721401, EPI_ISL_8721440, EPI_ISL_8721450, EPI_ISL_8721451, EPI_ISL_8721472, EPI_ISL_8721599, EPI_ISL_8721600, EPI_ISL_8721617, EPI_ISL_8721651, EPI_ISL_8721652, EPI_ISL_8721810, EPI_ISL_8721811, EPI_ISL_8721829, EPI_ISL_8721830, EPI_ISL_8721855, EPI_ISL_8721856, EPI_ISL_8721858, EPI_ISL_8721870, EPI_ISL_8721882, EPI_ISL_8721967			
see above	INSTITUTO BUTANTAN	Instituto Butantan	Antonio Jorge Martins; Claudia Renata dos Santos Barros; David Schlesinger; Debora Botequio Moretti; Dimas Tadeu Covas; Elaine Cristina Marqueze; Elaine Vieira Santos; Evandra Strazza Rodrigues; Heidge Fukumasu; Jayme Augusto de Souza-Neto; José Salvatore Leister Patané; Luiz Alcantara; Luiz Lehmann Coutinho; Maria Carolina Elias; Maurício Lacerda Nogueira; Rafael dos Santos Bezerra; Raul Machado Neto; Rejane Maria Tommasini Grotto; Ricardo Haddad; Sandra Coccuzzo Sampaio Vessoni; Simone Kashima; Svetoslav Nanev Slavov; Vincent Louis Viala
EPI_ISL_8501809, EPI_ISL_8501810, EPI_ISL_8501811	Instituto Adolfo Lutz - Regional de Bauru	Instituto Adolfo Lutz, Interdiciplinary Procedures Center, Strategic Laboratory	Ariadne Ferreira Amarante; Caio Vinicius Dias Lopes; Claudia Regina Gonçalves; Claudio Tavares Sacchi; Karoline Rodrigues Campos; Marlon Benedito Nascimento Santos
EPI_ISL_8170650, EPI_ISL_8501812, EPI_ISL_8501813	Instituto Adolfo Lutz - Regional de Campinas	Instituto Adolfo Lutz, Interdiciplinary Procedures Center, Strategic Laboratory	Ariadne Ferreira Amarante; Caio Vinicius Dias Lopes; Claudia Regina Gonçalves; Claudio Tavares Sacchi; Karoline Rodrigues Campos; Leonardo Tadeu de Araujo; Marlon Benedito Nascimento Santos
EPI_ISL_8501822, EPI_ISL_8501823, EPI_ISL_8501824, EPI_ISL_8501825, EPI_ISL_8501826, EPI_ISL_8501827, EPI_ISL_8501828, EPI_ISL_8501829, EPI_ISL_8501830			
see above	Instituto Adolfo Lutz - Regional de Presidente Prudente	Instituto Adolfo Lutz, Interdiciplinary Procedures Center, Strategic Laboratory	Ariadne Ferreira Amarante; Caio Vinicius Dias Lopes; Claudia Regina Gonçalves; Claudio Tavares Sacchi; Karoline Rodrigues Campos; Marlon Benedito Nascimento Santos
EPI_ISL_8501831, EPI_ISL_8501832, EPI_ISL_8501833	Instituto Adolfo Lutz - Regional de Ribeirao Preto	Instituto Adolfo Lutz, Interdiciplinary Procedures Center, Strategic Laboratory	Ariadne Ferreira Amarante; Caio Vinicius Dias Lopes; Claudia Regina Gonçalves; Claudio Tavares Sacchi; Karoline Rodrigues Campos; Marlon Benedito Nascimento Santos
EPI_ISL_7550075, EPI_ISL_8501804, EPI_ISL_8501805, EPI_ISL_8501814	Instituto Adolfo Lutz - Regional de Rio Claro	Instituto Adolfo Lutz, Interdiciplinary Procedures Center, Strategic Laboratory	Ariadne Ferreira Amarante; Caio Vinicius Dias Lopes; Claudia Regina Gonçalves; Claudio Tavares Sacchi; Karoline Rodrigues Campos; Marlon Benedito Nascimento Santos
EPI_ISL_8501806, EPI_ISL_8501807	Instituto Adolfo Lutz - Regional de Santo Andre	Instituto Adolfo Lutz, Interdiciplinary Procedures Center, Strategic Laboratory	Ariadne Ferreira Amarante; Caio Vinicius Dias Lopes; Claudia Regina Gonçalves; Claudio Tavares Sacchi; Karoline Rodrigues Campos; Marlon Benedito Nascimento Santos
EPI_ISL_8023723, EPI_ISL_8501808	Instituto Adolfo Lutz - Regional de Santos	Instituto Adolfo Lutz, Interdiciplinary Procedures Center, Strategic Laboratory	Ariadne Ferreira Amarante; Caio Vinicius Dias Lopes; Claudia Regina Gonçalves; Claudio Tavares Sacchi; Karoline Rodrigues Campos; Marlon Benedito Nascimento Santos
EPI_ISL_8026941, EPI_ISL_8501815, EPI_ISL_8501816, EPI_ISL_8501818, EPI_ISL_8501819	Instituto Adolfo Lutz - Regional de Sorocaba	Instituto Adolfo Lutz, Interdiciplinary Procedures Center, Strategic Laboratory	Ariadne Ferreira Amarante; Caio Vinicius Dias Lopes; Claudia Regina Gonçalves; Claudio Tavares Sacchi; Karoline Rodrigues Campos; Marlon Benedito Nascimento Santos
EPI_ISL_8501821	Instituto Adolfo Lutz - Regional de Taubate	Instituto Adolfo Lutz, Interdiciplinary Procedures Center, Strategic Laboratory	Ariadne Ferreira Amarante; Caio Vinicius Dias Lopes; Claudia Regina Gonçalves; Claudio Tavares Sacchi; Karoline Rodrigues Campos; Marlon Benedito Nascimento Santos
EPI_ISL_8023725, EPI_ISL_8501801, EPI_ISL_8501802, EPI_ISL_8501803, EPI_ISL_8519045	Instituto Adolfo Lutz Central	Instituto Adolfo Lutz, Interdiciplinary Procedures Center, Strategic Laboratory	Ariadne Ferreira Amarante; Caio Vinicius Dias Lopes; Claudia Regina Gonçalves; Claudio Tavares Sacchi; Karoline Rodrigues Campos; Marlon Benedito Nascimento Santos
EPI_ISL_8082284, EPI_ISL_8082285, EPI_ISL_8082289	Instituto de Biotecnologia - UNESP-Botucatu-SP	Instituto de Biotecnologia - UNESP-Botucatu-SP	Cecília Artico Banho; Cíntia Bittar; Fábio Sossai Possebon; Guilherme Campos; Helena Lage Ferreira; Jorge A. Petroli Marchesi; João Pessoa Araújo Jr.; Leila Sabrina Ullmann; Lívia Sacchetto; Maisa C. Pereira Parra; Marília Moraes; Maurício L. Nogueira; Paula Rahal; Paulo Inacio da Costa
EPI_ISL_8880895	LABORATORIO MUNICIPAL DE FLORIANOPOLIS - LAMUF	Laboratório de Bioinformática - Universidade Federal de Santa Catarina	Alexandra Crispim Boing; Ariane Nicaretta Amorim; Darcita Buerger Rovaris; Dayane Azevedo Padilha; Doris Sobral Marques Souza; Eric Kazuo Kawagoe; Fernanda Rosene Melo; Fernando Hartmann Barazzetti; Gislaine Fongaro; Glauber Wagner; João Augusto Brancher Fuck; Juliana Righetto Moser; Luiz Felipe Valter de Oliveira; Marcos André Schörner; Maria Luiza Bazzo; Marlei Pickler Debiasi dos Anjos; Milene Moehr de Moraes; Sandra Bianchini Fernandes; Vilmar Benetti Filho; on behalf of the Santa Catarina COVID-19 Genomic Surveillance Network (Genoma COVID SC)
EPI_ISL_6721332, EPI_ISL_6721336, EPI_ISL_6721339, EPI_ISL_6721346, EPI_ISL_6721355, EPI_ISL_6721359, EPI_ISL_6721368, EPI_ISL_6721376, EPI_ISL_6721385, EPI_ISL_6721390, EPI_ISL_6721396, EPI_ISL_6721401, EPI_ISL_6721414, EPI_ISL_6721419, EPI_ISL_6721776, EPI_ISL_6721784			
see above	LACEN - Laboratório Central de	Evandro Chagas Institute	Delana Andreza Melo Bezerra; Dielle Monteiro Teixeira; Jedson Ferreira Cardoso; Jessylene de Almeida Ferreira; Kenny da Costa Pinheiro; Luana Soares Barbagelata; Luana da Silva Soares; Mirleide Cordeiro dos Santos; Patrícia dos Santos Lobo; Rayssa Layna da Silva Bedran; Sandro

	Saúde Pública do Rio Grande do Norte		Patroca da Silva
EPI_ISL_7224971	LACEN do Distrito Federal	Instituto Adolfo Lutz Strategic Laboratory	Claudio Tavares Sacchi; Karoline Rodrigues Campos; Marlon Benedito Nascimento Santos
EPI_ISL_7550076	LACEN do Distrito Federal	Instituto Adolfo Lutz, Interdiciplinary Procedures Center, Strategic Laboratory	Claudio Tavares Sacchi; Karoline Rodrigues Campos
EPI_ISL_8708739, EPI_ISL_8708746, EPI_ISL_8708849	LACEN/AL	WallauLab on behalf of Fiocruz COVID-19 Genomic Surveillance Network	Alexandre Freitas da Silva; Andressa Oliveira; Antônio Marinho da Silva Neto; Cassia Docena; Claudeane Santo; Constância Flávia Junqueira Ayres; Filipe Zimmer Dezordi; Gabriel Luz Wallau; Giovanna Albernaz; Gustavo Barbosa de Lima; Hazerral Oliveira; Lais Ceschini Machado; Leandro de Mattos; Lilian Caroliny Amorim Silva; Marcelo Henrique dos Santos Paiva; Matheus Filgueira Bezerra; Sinval Pinto Brandão Filho; Túlio de Lima Campos
EPI_ISL_8466824, EPI_ISL_8466831, EPI_ISL_8466832, EPI_ISL_8466833, EPI_ISL_8466834, EPI_ISL_8466835, EPI_ISL_8466836, EPI_ISL_8466837, EPI_ISL_8466838, EPI_ISL_8466840, EPI_ISL_8466842, EPI_ISL_8466844, EPI_ISL_8466846, EPI_ISL_8466849, EPI_ISL_8466850, EPI_ISL_8466853, EPI_ISL_8466894, EPI_ISL_8466906, EPI_ISL_8708717, EPI_ISL_8708718, EPI_ISL_8708722, EPI_ISL_8708724, EPI_ISL_8708725, EPI_ISL_8708726, EPI_ISL_8708729, EPI_ISL_8708731, EPI_ISL_8708732, EPI_ISL_8708733, EPI_ISL_8708734, EPI_ISL_8708737, EPI_ISL_8708740, EPI_ISL_8708742, EPI_ISL_8708743, EPI_ISL_8708744, EPI_ISL_8708745, EPI_ISL_8708747, EPI_ISL_8708748, EPI_ISL_8708749, EPI_ISL_8708750, EPI_ISL_8708751, EPI_ISL_8708752, EPI_ISL_8708754, EPI_ISL_8708755, EPI_ISL_8708756, EPI_ISL_8708757, EPI_ISL_8708758, EPI_ISL_8708759, EPI_ISL_8708760, EPI_ISL_8708761, EPI_ISL_8708762, EPI_ISL_8708763, EPI_ISL_8708764, EPI_ISL_8708765, EPI_ISL_8708766, EPI_ISL_8708767, EPI_ISL_8708768, EPI_ISL_8708769, EPI_ISL_8708770, EPI_ISL_8708771, EPI_ISL_8708772, EPI_ISL_8708773, EPI_ISL_8708774, EPI_ISL_8708775, EPI_ISL_8708776, EPI_ISL_8708777, EPI_ISL_8708778, EPI_ISL_8708779, EPI_ISL_8708780, EPI_ISL_8708781, EPI_ISL_8708782, EPI_ISL_8708784, EPI_ISL_8708785, EPI_ISL_8708786, EPI_ISL_8708788, EPI_ISL_8708789, EPI_ISL_8708791, EPI_ISL_8708793, EPI_ISL_8708794, EPI_ISL_8708795, EPI_ISL_8708796, EPI_ISL_8708797, EPI_ISL_8708799, EPI_ISL_8708801, EPI_ISL_8708802, EPI_ISL_8708803, EPI_ISL_8708804, EPI_ISL_8708805, EPI_ISL_8708806, EPI_ISL_8708808, EPI_ISL_8708809, EPI_ISL_8708810, EPI_ISL_8708811, EPI_ISL_8708812, EPI_ISL_8708813, EPI_ISL_8708814, EPI_ISL_8708815, EPI_ISL_8708816, EPI_ISL_8708817, EPI_ISL_8708818, EPI_ISL_8708819, EPI_ISL_8708820, EPI_ISL_8708821, EPI_ISL_8708824, EPI_ISL_8708825, EPI_ISL_8708826, EPI_ISL_8708828, EPI_ISL_8708829, EPI_ISL_8708831, EPI_ISL_8708832, EPI_ISL_8708833, EPI_ISL_8708834, EPI_ISL_8708835, EPI_ISL_8708837, EPI_ISL_8708838, EPI_ISL_8708839, EPI_ISL_8708842, EPI_ISL_8708843, EPI_ISL_8708844, EPI_ISL_8708845, EPI_ISL_8708846, EPI_ISL_8708847, EPI_ISL_8708851, EPI_ISL_8708852, EPI_ISL_8708853, EPI_ISL_8708854, EPI_ISL_8708857, EPI_ISL_8708858, EPI_ISL_8708859, EPI_ISL_8708860, EPI_ISL_8708861, EPI_ISL_8708866, EPI_ISL_8708867, EPI_ISL_8708868, EPI_ISL_8708869, EPI_ISL_8708870, EPI_ISL_8708871, EPI_ISL_8708872, EPI_ISL_8708873			
see above	LACEN/PE	WallauLab on behalf of Fiocruz COVID-19 Genomic Surveillance Network	Alexandre Freitas da Silva; Antonio Marinho da Silva Neto; Cassia Docena; Constância Flávia Junqueira Ayres; Filipe Zimmer Dezordi; Gabriel Luz Wallau; Gustavo Barbosa de Lima; Lais Ceschini Machado; Lilian Caroliny Amorim Silva; Marcelo Henrique dos Santos Paiva; Matheus Filgueira Bezerra; Sinval Pinto Brandão Filho
EPI_ISL_6704867	LANCET LABORATORY	National Institute for Communicable Diseases of the National Health Laboratory Service	Amoako DG; Bhiman JN; Everatt J; Ismail A; Mahlangu B; Mnguni A; Mohale T; Ntuli N; Scheepers C; Wolter N
EPI_ISL_6901960, EPI_ISL_6901961, EPI_ISL_7473154, EPI_ISL_7699339, EPI_ISL_7699342, EPI_ISL_7699344, EPI_ISL_7699346, EPI_ISL_7699349, EPI_ISL_7699351, EPI_ISL_7699353, EPI_ISL_7963778, EPI_ISL_8151799, EPI_ISL_8151800, EPI_ISL_8151801, EPI_ISL_8151802, EPI_ISL_8151803, EPI_ISL_8151804, EPI_ISL_8151805, EPI_ISL_8151807, EPI_ISL_8151808, EPI_ISL_8151809, EPI_ISL_8317587, EPI_ISL_8317588, EPI_ISL_8317589, EPI_ISL_8317591, EPI_ISL_8317593, EPI_ISL_8317594, EPI_ISL_8317595, EPI_ISL_8317596, EPI_ISL_8317597			
see above	LATE - Laboratório de Técnicas Especiais - Hospital Israelita Albert Einstein	LATE - Laboratório de Técnicas Especiais - Hospital Israelita Albert Einstein	Alexandre Hideaki Takara; Ana Paula Moreira Salles; Anelisie da Silva Santos; Deyvid Amgarten; Erick Gustavo Dorlass; Fernanda de Mello Malta; João Renato Rebello Pinho; Luiz Vicente Rizzo; Marcio Anunciacao Menezes; Pedro Henrique Sebe Rodrigues; Raquel Riyuzo
EPI_ISL_2385528	Laboratorio Central Noel Nutels	Bioinformatics Laboratory / LNCC	Alessandra P Lamarca; Alexandra L Gerber; Amilcar Tanuri; Ana Paula de C Guimaraes; Ana Tereza R Vasconcelos; Andrea Cony Cavalcanti; Caio Luiz Pereira Ribeiro; Cassia Alves; Cintia Policarpo; Claudia Maria Braga de Mello; Cristiane Gomes da Silva; Diana Mariani; Douglas Terra Machado; Flavio Dias da Silva; Gleidson da Silva de Oliveira; Leandro Magalhaes de Souza; Liliane Cavalcante; Luiz G P de Almeida; Marcio Henrique de Oliveira Garcia; Mario Sergio Ribeiro; Ronaldo da Silva F Jr; Silvia Carvalho
EPI_ISL_8430487	Laboratorio Central de Saude Publica do Estado da Paraiba (LACEN/PB)	Laboratory of Respiratory Viruses and Measles, Oswaldo Cruz Institute, FIOCRUZ	Alice Sampaio Rocha; Bruna Mendonça da Silva; Dalane Loudal Florentino Teixeira; Elisa Cavalcante Pereira; Fernando Motta; Ighor Arantes; Joao Felipe Bezerra; Jéssica Graça Macedo de Carvalho; Larissa Macedo Pinto; Luciana Appolinario; Marilda Siqueira on behalf of the Fiocruz COVID-19 Genomic Surveillance Network; Paola Resende; Victor Guimaraes
EPI_ISL_8151564, EPI_ISL_8266384, EPI_ISL_8266390, EPI_ISL_8266391, EPI_ISL_8266392, EPI_ISL_8266393, EPI_ISL_8266394, EPI_ISL_8266395, EPI_ISL_8266396, EPI_ISL_8266397, EPI_ISL_8266398, EPI_ISL_8266399, EPI_ISL_8266400, EPI_ISL_8266401, EPI_ISL_8266402, EPI_ISL_8266403, EPI_ISL_8266404, EPI_ISL_8266405, EPI_ISL_8266406, EPI_ISL_8266407, EPI_ISL_8266408, EPI_ISL_8266409, EPI_ISL_8266410, EPI_ISL_8266411, EPI_ISL_8266412, EPI_ISL_8266413, EPI_ISL_8266414, EPI_ISL_8266415, EPI_ISL_8266416, EPI_ISL_8266417, EPI_ISL_8266418, EPI_ISL_8266419, EPI_ISL_8266420, EPI_ISL_8266421, EPI_ISL_8266422, EPI_ISL_8266423, EPI_ISL_8325362, EPI_ISL_8325363, EPI_ISL_8325364, EPI_ISL_8325365, EPI_ISL_8325366, EPI_ISL_8325367, EPI_ISL_8325368, EPI_ISL_8325369, EPI_ISL_8325370, EPI_ISL_8325371, EPI_ISL_8325372, EPI_ISL_8325373, EPI_ISL_8325374, EPI_ISL_8430488, EPI_ISL_8797052, EPI_ISL_8797053, EPI_ISL_8797054, EPI_ISL_8797055, EPI_ISL_8797056, EPI_ISL_8797057, EPI_ISL_8797058, EPI_ISL_8797059, EPI_ISL_8797060, EPI_ISL_8797061, EPI_ISL_8797069, EPI_ISL_8797072, EPI_ISL_8797073, EPI_ISL_8797074, EPI_ISL_8797075, EPI_ISL_8797076, EPI_ISL_8797077, EPI_ISL_8797078, EPI_ISL_8797079, EPI_ISL_8797080, EPI_ISL_8797081, EPI_ISL_8797082, EPI_ISL_8797083, EPI_ISL_8797084, EPI_ISL_8797085, EPI_ISL_8797086, EPI_ISL_8797087, EPI_ISL_8797088, EPI_ISL_8797089, EPI_ISL_8797090, EPI_ISL_8797091, EPI_ISL_8797092, EPI_ISL_8797093, EPI_ISL_8797094, EPI_ISL_8797095, EPI_ISL_8797096, EPI_ISL_8797097, EPI_ISL_8797098, EPI_ISL_8797099, EPI_ISL_8797100, EPI_ISL_8797101, EPI_ISL_8797102, EPI_ISL_8797103, EPI_ISL_8797104, EPI_ISL_8797105, EPI_ISL_8797106, EPI_ISL_8797107, EPI_ISL_8797108, EPI_ISL_8797109, EPI_ISL_8797110, EPI_ISL_8797111, EPI_ISL_8797112, EPI_ISL_8797113, EPI_ISL_8797114, EPI_ISL_8797115, EPI_ISL_8797116, EPI_ISL_8797117, EPI_ISL_8797118, EPI_ISL_8797119, EPI_ISL_8797120, EPI_ISL_8797121, EPI_ISL_8797123, EPI_ISL_8797124, EPI_ISL_8797125, EPI_ISL_8797126, EPI_ISL_8797127, EPI_ISL_8797128, EPI_ISL_8797129, EPI_ISL_8797130, EPI_ISL_8797131, EPI_ISL_8797133, EPI_ISL_8797134, EPI_ISL_8797136, EPI_ISL_8797137, EPI_ISL_8797138, EPI_ISL_8797139, EPI_ISL_8797140, EPI_ISL_8797141, EPI_ISL_8797142, EPI_ISL_8797143, EPI_ISL_8797144, EPI_ISL_8797145, EPI_ISL_8797146, EPI_ISL_8797147, EPI_ISL_8797149			
see above	Laboratorio Central de Saude Publica do Estado de Santa Catarina (LACEN/SC)	Laboratory of Respiratory Viruses and Measles, Oswaldo Cruz Institute, FIOCRUZ	Alice Sampaio Rocha; Ana Beatriz Machado Lima; Bruna Mendonça da Silva; Darcita Buerger Rovaris; Elisa Cavalcante Pereira; Fernando Motta; Ighor Arantes; Jéssica Graça Macedo de Carvalho; Larissa Macedo Pinto; Luciana Appolinario; Luiz Fernando Lopez Tort; Marilda Siqueira on behalf of the Fiocruz COVID-19 Genomic Surveillance Network; Mia Ferreira de Araujo; Natália Valente da Silva; Paola Resende; Sandra Bianchini Fernandes; Victor Guimaraes
EPI_ISL_8633918, EPI_ISL_8633919, EPI_ISL_8633920, EPI_ISL_8633921, EPI_ISL_8633922, EPI_ISL_8633923, EPI_ISL_8633924, EPI_ISL_8633925, EPI_ISL_8633926, EPI_ISL_8633927, EPI_ISL_8633928, EPI_ISL_8633929, EPI_ISL_8633930, EPI_ISL_8633931, EPI_ISL_8633932, EPI_ISL_8633933, EPI_ISL_8633934, EPI_ISL_8633935, EPI_ISL_8633936, EPI_ISL_8633937			
see above	Laboratorio Central de Saude Publica do Estado do Espirito Santo (LACEN/ES)	Laboratory of Respiratory Viruses and Measles, Oswaldo Cruz Institute, FIOCRUZ	Alice Sampaio Rocha; Bruna Mendonça da Silva; Elisa Cavalcante Pereira; Fernando Motta; Ighor Arantes; Jéssica Graça Macedo de Carvalho; Larissa Macedo Pinto; Luciana Appolinario; Marilda Siqueira on behalf of the Fiocruz COVID-19 Genomic Surveillance Network; Paola Resende; Rodrigo Ribeiro Rodrigues; Victor Guimaraes
EPI_ISL_3190392, EPI_ISL_3190393, EPI_ISL_3190394, EPI_ISL_3190395, EPI_ISL_3190396, EPI_ISL_3190397, EPI_ISL_3190398, EPI_ISL_3190399, EPI_ISL_3190400, EPI_ISL_3190401, EPI_ISL_3190402, EPI_ISL_3190403, EPI_ISL_3190404, EPI_ISL_3235268, EPI_ISL_3235269, EPI_ISL_3235270, EPI_ISL_3235271, EPI_ISL_3235272, EPI_ISL_3235273, EPI_ISL_3235274, EPI_ISL_3235275, EPI_ISL_3539831, EPI_ISL_3539832, EPI_ISL_3539833, EPI_ISL_3539834, EPI_ISL_3539835, EPI_ISL_3539836, EPI_ISL_3539837, EPI_ISL_3539838, EPI_ISL_3539839, EPI_ISL_3828023, EPI_ISL_3832392, EPI_ISL_3832401, EPI_ISL_3832402, EPI_ISL_3832403, EPI_ISL_3832404, EPI_ISL_3832405, EPI_ISL_3832406, EPI_ISL_3832407, EPI_ISL_3832408, EPI_ISL_3832409, EPI_ISL_4212798, EPI_ISL_4212799, EPI_ISL_4212800, EPI_ISL_4212801, EPI_ISL_4212802, EPI_ISL_4212803, EPI_ISL_4212804, EPI_ISL_4212805, EPI_ISL_4212806, EPI_ISL_4212807, EPI_ISL_4212808, EPI_ISL_4212809, EPI_ISL_4212810, EPI_ISL_4212828, EPI_ISL_4212867, EPI_ISL_4212873, EPI_ISL_4212876, EPI_ISL_4212886, EPI_ISL_4212892, EPI_ISL_4212896, EPI_ISL_4212897, EPI_ISL_4212900, EPI_ISL_4212901, EPI_ISL_4212912, EPI_ISL_4212918, EPI_ISL_4271114, EPI_ISL_4418871, EPI_ISL_4418872, EPI_ISL_4418873, EPI_ISL_4418874, EPI_ISL_4418875, EPI_ISL_4418876, EPI_ISL_4418877, EPI_ISL_4418878, EPI_ISL_4919923, EPI_ISL_4919924, EPI_ISL_4919925, EPI_ISL_4919926, EPI_ISL_5328874, EPI_ISL_5328875, EPI_ISL_5328876, EPI_ISL_5328877, EPI_ISL_5328878, EPI_ISL_5328879, EPI_ISL_5328880, EPI_ISL_5328881, EPI_ISL_5328882, EPI_ISL_5328883, EPI_ISL_5328884, EPI_ISL_5328885, EPI_ISL_5328886, EPI_ISL_5328887, EPI_ISL_5328888, EPI_ISL_5328889, EPI_ISL_5328890, EPI_ISL_5328891, EPI_ISL_5328892, EPI_ISL_5328893, EPI_ISL_5328894, EPI_ISL_5328895, EPI_ISL_5328896, EPI_ISL_5328897, EPI_ISL_5328898, EPI_ISL_5328899, EPI_ISL_5328900, EPI_ISL_5328901, EPI_ISL_5328902, EPI_ISL_5328903, EPI_ISL_5328904, EPI_ISL_5328905, EPI_ISL_5328906, EPI_ISL_5328907, EPI_ISL_5328908, EPI_ISL_5328909, EPI_ISL_5328910, EPI_ISL_5328911, EPI_ISL_5328912, EPI_ISL_5328913, EPI_ISL_5328914, EPI_ISL_5328915, EPI_ISL_5328916, EPI_ISL_5328917, EPI_ISL_5328918, EPI_ISL_5328919, EPI_ISL_5328920, EPI_ISL_5328921, EPI_ISL_5328922, EPI_ISL_5328923, EPI_ISL_5328924, EPI_ISL_5328925, EPI_ISL_5328926, EPI_ISL_5328927, EPI_ISL_5328928, EPI_ISL_5328929, EPI_ISL_5328930, EPI_ISL_5328931, EPI_ISL_5328932, EPI_ISL_5328933, EPI_ISL_5328934, EPI_ISL_5328935, EPI_ISL_5328936, EPI_ISL_5328937, EPI_ISL_5328938, EPI_ISL_5328939, EPI_ISL_5328940, EPI_ISL_5328941, EPI_ISL_5328942, EPI_ISL_5328943, EPI_ISL_5328944, EPI_ISL_5328945, EPI_ISL_5328946, EPI_ISL_5328947, EPI_ISL_5328948, EPI_ISL_5328949, EPI_ISL_5328950, EPI_ISL_5328951, EPI_ISL_5328952, EPI_ISL_5328953, EPI_ISL_5328954, EPI_ISL_5328955, EPI_ISL_5328956, EPI_ISL_5328957, EPI_ISL_5328958, EPI_ISL_5328959, EPI_ISL_5328960, EPI_ISL_5328961, EPI_ISL_5328962, EPI_ISL_5328963, EPI_ISL_5328964, EPI_ISL_5328965, EPI_ISL_5328966, EPI_ISL_5328967, EPI_ISL_5328968, EPI_ISL_5328969, EPI_ISL_5328970, EPI_ISL_5328971, EPI_ISL_5328972, EPI_ISL_5328973, EPI_ISL_5328974, EPI_ISL_5328975, EPI_ISL_5328976, EPI_ISL_5328977, EPI_ISL_5329018, EPI_ISL_5329019, EPI_ISL_5329020, EPI_ISL_5329021, EPI_ISL_5329022, EPI_ISL_5329025, EPI_ISL_5329027, EPI_ISL_5329032, EPI_ISL_5329033, EPI_ISL_5329034, EPI_ISL_5329035, EPI_ISL_5329044, EPI_ISL_5329045, EPI_ISL_5329046, EPI_ISL_5329047, EPI_ISL_5329048, EPI_ISL_5329049, EPI_ISL_5329054, EPI_ISL_5329056, EPI_ISL_5329057, EPI_ISL_5329061, EPI_ISL_5329062, EPI_ISL_5329068, EPI_ISL_6173085, EPI_ISL_6173086, EPI_ISL_6898941, EPI_ISL_6898942, EPI_ISL_6898943, EPI_ISL_6898944, EPI_ISL_6898945, EPI_ISL_6898984, EPI_ISL_6899067, EPI_ISL_6899073, EPI_ISL_6943957, EPI_ISL_6943958, EPI_ISL_6943959, EPI_ISL_6943960, EPI_ISL_6943961, EPI_ISL_6943962, EPI_ISL_6943977, EPI_ISL_6943978, EPI_ISL_7456176, EPI_ISL_7456183, EPI_ISL_7456190, EPI_ISL_7456198, EPI_ISL_7456207, EPI_ISL_7456208, EPI_ISL_7456209, EPI_ISL_7456211, EPI_ISL_7456212, EPI_ISL_8013192, EPI_ISL_8013193, EPI_ISL_8013194, EPI_ISL_8013195, EPI_ISL_8013196, EPI_ISL_8013197, EPI_ISL_8013198, EPI_ISL_8013199, EPI_ISL_8013200, EPI_ISL_8013201, EPI_ISL_8325342, EPI_ISL_8325343, EPI_ISL_8325344, EPI_ISL_8325345, EPI_ISL_8325346, EPI_ISL_8325347, EPI_ISL_8325348, EPI_ISL_8325349, EPI_ISL_8325350, EPI_ISL_8325351, EPI_ISL_8325352, EPI_ISL_8325353, EPI_ISL_8325354, EPI_ISL_8325355, EPI_ISL_8325356, EPI_ISL_8325357, EPI_ISL_8325358, EPI_ISL_8325359, EPI_ISL_8325360, EPI_ISL_8325361, EPI_ISL_8351332, EPI_ISL_8430550, EPI_ISL_8634026			
see above	Laboratorio Central de Saude Publica do Estado do Rio Grande do Norte (LACEN/RN)	Laboratory of Respiratory Viruses and Measles, Oswaldo Cruz Institute, FIOCRUZ	Agatha Soares; Alice Sampaio Rocha; Ana Carolina Mendonca; Ana Paula Ferreira Costa; Anna Carolina Paixao; Antonnyo Palmielly Diogenes Lima; Aurélio de Oliveira Bento; Bruna Mendonça da Silva; Elisa Cavalcante Pereira; Fernando Motta; Gessika Brenna Costa Alves; Heglayne Pereira Vital da Silva; Iago de Souza Gomes; Ighor Arantes; Isabelle Cristina Clemente dos Santos; Janaina Sonale Cavalcante Nogueira de Oliveira; Jayra Juliana Paiva Alves Abrantes; Jonas José da Silva; Jéssica Graça Macedo de Carvalho; Larissa Macedo Pinto; Luciana Appolinario; Marilda Siqueira on behalf of the Fiocruz COVID-19 Genomic Surveillance Network; Paola Resende; Renata Serrano Lopes; Taina Venas; Themis Rocha de Souza; Victor Guimaraes; Vítor Gabriel Saldanha Fernandes
EPI_ISL_8013188, EPI_ISL_8325214, EPI_ISL_8332308, EPI_ISL_8430493, EPI_ISL_8430494, EPI_ISL_8430495, EPI_ISL_8430496, EPI_ISL_8430497, EPI_ISL_8430498, EPI_ISL_8430499, EPI_ISL_8430500, EPI_ISL_8430502, EPI_ISL_8430503, EPI_ISL_8430504, EPI_ISL_8430505, EPI_ISL_8430506, EPI_ISL_8430507, EPI_ISL_8430508, EPI_ISL_8430509, EPI_ISL_8430510, EPI_ISL_8430511, EPI_ISL_8430512, EPI_ISL_8430513, EPI_ISL_8430514, EPI_ISL_8430515, EPI_ISL_8430516, EPI_ISL_8430517, EPI_ISL_8430518, EPI_ISL_8430519, EPI_ISL_8430520, EPI_ISL_8430521, EPI_ISL_8430522, EPI_ISL_8430523, EPI_ISL_8430524, EPI_ISL_8430525, EPI_ISL_8430526, EPI_ISL_8430527, EPI_ISL_8430528, EPI_ISL_8430529, EPI_ISL_8430530, EPI_ISL_8430531, EPI_ISL_8430532, EPI_ISL_8430533, EPI_ISL_8430534, EPI_ISL_8430535, EPI_ISL_8430536, EPI_ISL_8430537, EPI_ISL_8430538, EPI_ISL_8430539, EPI_ISL_8430540, EPI_ISL_8430541, EPI_ISL_8430542, EPI_ISL_8430543, EPI_ISL_8430544, EPI_ISL_8430545, EPI_ISL_8430546, EPI_ISL_8430548, EPI_ISL_8430549, EPI_ISL_8633983, EPI_ISL_8633984, EPI_ISL_8633985, EPI_ISL_8633986, EPI_ISL_8633987, EPI_ISL_8633988, EPI_ISL_8633989, EPI_ISL_8633990, EPI_ISL_8633991, EPI_ISL_8633992, EPI_ISL_8633993, EPI_ISL_8633994, EPI_ISL_8633995, EPI_ISL_8633996, EPI_ISL_8633997, EPI_ISL_8633998, EPI_ISL_8633999, EPI_ISL_8634000, EPI_ISL_8634001, EPI_ISL_8634002, EPI_ISL_8634003, EPI_ISL_8634004, EPI_ISL_8634005, EPI_ISL_8634006, EPI_ISL_8634007, EPI_ISL_8634008, EPI_ISL_8634009, EPI_ISL_8634010, EPI_ISL_8634011, EPI_ISL_8634012, EPI_ISL_8634013, EPI_ISL_8634014, EPI_ISL_8634015, EPI_ISL_8634016, EPI_ISL_8634017, EPI_ISL_8634018, EPI_ISL_8634019, EPI_ISL_8634020, EPI_ISL_8634021, EPI_ISL_8634022, EPI_ISL_8634023, EPI_ISL_8634024, EPI_ISL_8634025, EPI_ISL_8797026, EPI_ISL_8797027, EPI_ISL_8797028, EPI_ISL_8797029, EPI_ISL_8797030, EPI_ISL_8797031, EPI_ISL_8797032, EPI_ISL_8797033			
see above	Laboratorio Central de Saude Publica do Estado do Rio de Janeiro (LACEN/RJ)	Laboratory of Respiratory Viruses and Measles, Oswaldo Cruz Institute, FIOCRUZ	Alice Sampaio Rocha; Andrea Cony Cavalcanti; Bruna Mendonça da Silva; Elisa Cavalcante Pereira; Fernando Motta; Ighor Arantes; Jéssica Graça Macedo de Carvalho; Larissa Macedo Pinto; Luciana Appolinario; Marilda Siqueira on behalf of the Fiocruz COVID-19 Genomic Surveillance Network; Paola Resende; Victor Guimaraes
EPI_ISL_7309168	Laboratorio Central de Saude Publica do Rio Grande do Sul/Centro Estadual de Vigilancia em Saude	Centro de Desenvolvimento Cientifico e Tecnologico (CDCT)/Centro Estadual de Vigilancia em Saude	Alana Rossetti; Cláudia Maria Dornelles da Silva; Fernanda Marques Godinho; Larissa Vitoria da Silva; Ludmila Fiorenzano Baethgen; Miguel S Andrade.; Regina Bones Barcellos; Richard Steiner Salvato; Tatiana Schaffer Gregianini
EPI_ISL_8665286	Laboratorio de Pesquisa em Virologia, FAMERP, SJRP	Laboratorio de Pesquisa em Virologia, FAMERP, SJRP	Beatriz de Carvalho Marques; Cecília Artico Banho; Cíntia Bittar; Fábio Sossai Possebon; Guilherme Campos; Helena Lage Ferreira; Jorge A. Petroli Marchesi; João Pessoa Araújo Jr.; Leila Sabrina Ullmann; Lívia Sacchetto; Maisa C. Pereira Parra; Marília Moraes; Maurício L. Nogueira.; Paula Rahal; Paulo Inacio da Costa
EPI_ISL_8633858, EPI_ISL_8633859, EPI_ISL_8633860, EPI_ISL_8633861, EPI_ISL_8633862, EPI_ISL_8633863, EPI_ISL_8633864, EPI_ISL_8633865, EPI_ISL_8633866, EPI_ISL_8633867, EPI_ISL_8633868, EPI_ISL_8633869, EPI_ISL_8633870, EPI_ISL_8633871, EPI_ISL_8633872, EPI_ISL_8633873, EPI_ISL_8633874, EPI_ISL_8633875, EPI_ISL_8633876, EPI_ISL_8633877, EPI_ISL_8633878, EPI_ISL_8633879, EPI_ISL_8633880, EPI_ISL_8633881, EPI_ISL_8633883, EPI_ISL_8633884, EPI_ISL_8633885, EPI_ISL_8633886, EPI_ISL_8633887, EPI_ISL_8633888, EPI_ISL_8633889, EPI_ISL_8633890, EPI_ISL_8633891, EPI_ISL_8633892, EPI_ISL_8633893, EPI_ISL_8633894, EPI_ISL_8633895, EPI_ISL_8633896, EPI_ISL_8797034, EPI_ISL_8797035, EPI_ISL_8797036, EPI_ISL_8797037, EPI_ISL_8797038, EPI_ISL_8797039, EPI_ISL_8797040, EPI_ISL_8797041, EPI_ISL_8797042, EPI_ISL_8797043, EPI_ISL_8797044, EPI_ISL_8797045, EPI_ISL_8797046, EPI_ISL_8797047, EPI_ISL_8797048, EPI_ISL_8797049, EPI_ISL_8797050, EPI_ISL_8797051			
see above	Laboratory of Respiratory Viruses and Measles, Oswaldo Cruz Institute, FIOCRUZ	Laboratory of Respiratory Viruses and Measles, Oswaldo Cruz Institute, FIOCRUZ	Alice Sampaio Rocha; Bruna Mendonça da Silva; Elisa Cavalcante Pereira; Fernando Motta; Ighor Arantes; Jéssica Graça Macedo de Carvalho; Larissa Macedo Pinto; Luciana Appolinario; Marilda Siqueira on behalf of the Fiocruz COVID-19 Genomic Surveillance Network; Paola Resende; Victor Guimaraes
EPI_ISL_8515148, EPI_ISL_8554881, EPI_ISL_8554884, EPI_ISL_8554885, EPI_ISL_8554886, EPI_ISL_8554887, EPI_ISL_8554888, EPI_ISL_8554889, EPI_ISL_8554890, EPI_ISL_8554891, EPI_ISL_8554892, EPI_ISL_8554893, EPI_ISL_8554894, EPI_ISL_8554896, EPI_ISL_8554898, EPI_ISL_8554899, EPI_ISL_8554900, EPI_ISL_8554901, EPI_ISL_8554930, EPI_ISL_8554945, EPI_ISL_8554956, EPI_ISL_8554957			
see above	Laboratório Central de Saúde Pública do Amazonas - LACEN-AM	Laboratório de Ecologia de Doenças Transmissíveis na Amazônia, Instituto Leônidas e Maria Deane - Fiocruz Amazônia	André Corado; Arlesson Viana Da Silva; Dejanane Silva; Felipe Naveca on behalf of the Fiocruz COVID-19 Genomic Surveillance Network; Fernanda Nascimento; George Silva; Luciana Gonçalves; Maria Júlia Brandão; Matilde Mejía; Valdinete Nascimento; Victor Souza
EPI_ISL_8170818	Laboratório Central de Saúde Pública do Estado do Tocantins (LACEN/TO)	Bioinformatics and Biotechnology Laboratory (Labinftec/Federal University of Tocantins, Campus of Gurupi)	Fabrício Souza Campos; Jucimária Dantas Galvão; Raíssa Nunes dos Santos; Ueric José Borges de Souza
EPI_ISL_2241501, EPI_ISL_2241502, EPI_ISL_2241503, EPI_ISL_2241504, EPI_ISL_2241505, EPI_ISL_2241506, EPI_ISL_2241507, EPI_ISL_2241508, EPI_ISL_2241509, EPI_ISL_2241510, EPI_ISL_2241511, EPI_ISL_2241512, EPI_ISL_2241513, EPI_ISL_2241534, EPI_ISL_2241537, EPI_ISL_2241538, EPI_ISL_2241539, EPI_ISL_2241543, EPI_ISL_2241544, EPI_ISL_2241545, EPI_ISL_2241546, EPI_ISL_2241548, EPI_ISL_2241549, EPI_ISL_2241550, EPI_ISL_2241551, EPI_ISL_2241553, EPI_ISL_2241574, EPI_ISL_2241606, EPI_ISL_2241608, EPI_ISL_2241610, EPI_ISL_2308399, EPI_ISL_2308404, EPI_ISL_2308407, EPI_ISL_2308433, EPI_ISL_2308439, EPI_ISL_2308460, EPI_ISL_2308464			
see above	Laboratório Central de Saúde Pública do Rio Grande do Norte	Coordenação Geral de Laboratórios de Saúde Pública (CGLAB/DAEVS/SVS/MS)	Vagner Fonseca; et al.
EPI_ISL_8128237, EPI_ISL_8175742, EPI_ISL_8179172, EPI_ISL_8179432, EPI_ISL_8179641	Laboratório de Análises Clínicas do Centro Universitário FMABC	Laboratório de Análises Clínicas do Centro Universitário FMABC	Beatriz da Costa Aguiar Alves; Fernando Luiz Affonso Fonseca
EPI_ISL_8128236	Laboratório de Análises Clínicas do centro Universitário FMABC	Laboratório de Análises Clínicas do centro Universitário FMABC	Beatriz da Costa Aguiar Alves; Fernando Luiz Affonso Fonseca

EPI_ISL_2466139, EPI_ISL_2466140, EPI_ISL_2466141, EPI_ISL_2466142, EPI_ISL_2466143, EPI_ISL_2466144, EPI_ISL_2466145, EPI_ISL_2466146, EPI_ISL_2466147, EPI_ISL_2466148, EPI_ISL_2466149, EPI_ISL_2466150, EPI_ISL_2466151, EPI_ISL_2466152, EPI_ISL_2466153, EPI_ISL_2466154, EPI_ISL_2466155, EPI_ISL_2466156, EPI_ISL_2466157, EPI_ISL_2466158, EPI_ISL_2466159, EPI_ISL_2466160, EPI_ISL_2466161, EPI_ISL_2466162, EPI_ISL_2466163, EPI_ISL_2466164, EPI_ISL_2466165, EPI_ISL_2466166, EPI_ISL_2466167, EPI_ISL_2466168, EPI_ISL_2466169, EPI_ISL_2466170, EPI_ISL_2466171, EPI_ISL_2466172, EPI_ISL_2466173, EPI_ISL_2466174, EPI_ISL_2466175, EPI_ISL_2466176, EPI_ISL_2466177, EPI_ISL_2466178, EPI_ISL_2466179, EPI_ISL_2466180, EPI_ISL_2466181, EPI_ISL_2466182, EPI_ISL_2466183, EPI_ISL_2466184, EPI_ISL_2466185, EPI_ISL_2466186, EPI_ISL_2466188, EPI_ISL_2466189, EPI_ISL_2466200, EPI_ISL_2466201, EPI_ISL_2466202, EPI_ISL_2466203, EPI_ISL_2466204, EPI_ISL_2466205, EPI_ISL_2466206, EPI_ISL_2466207, EPI_ISL_2466208, EPI_ISL_2466209, EPI_ISL_2466210, EPI_ISL_2466211, EPI_ISL_2466212, EPI_ISL_2466214, EPI_ISL_2466215, EPI_ISL_2466216, EPI_ISL_2466217, EPI_ISL_2466218, EPI_ISL_2466219, EPI_ISL_2466220, EPI_ISL_2466221, EPI_ISL_2466222, EPI_ISL_2466223, EPI_ISL_2466224, EPI_ISL_2466225, EPI_ISL_2466226, EPI_ISL_2466227, EPI_ISL_2466228, EPI_ISL_2466229, EPI_ISL_2536268			
see above	Laboratório de Biologia Molecular de Doenças Infecciosas e do Câncer (LADIC - UFRN)	Laboratory of Respiratory Viruses and Measles, Oswaldo Cruz Institute, FIOCRUZ	Alice Sampaio Rocha; Ana Carolina Mendonca; Anna Carolina Paixao; Elisa Cavalcante Pereira; Fernando Motta; Josélio Araújo; Luciana Appolinario; Marilda Siqueira on behalf of the Fiocruz COVID-19 Genomic Surveillance Network; Paola Resende; Renata Serrano Lopes; Taina Venas
EPI_ISL_8621509	MATERNIDADE ESCOLA ASSIS CHATEAUBRIAND	Analytical Competence Molecular Epidemiology Lab/ACME, Oswaldo Cruz Foundation, Ceara (FIOCRUZ CE)	Carlos Leonardo de Aragao Araujo; Cleber Furtado Aksenen; Fabio Miyajima; Fernando Braga Stehling; Jamille Maria Mendes Bezerra; Joaquim Cesar do Nascimento Sousa Junior; Pedro Miguel Carneiro Jeronimo; Suzana Porto Almeida & Igor Oliveira Duarte on behalf of COVID-19 FIOCRUZ Genomic Network; Thais Ferreira de Oliveira; Thais de Oliveira Costa; Ticiane Cavalcante de Souza; Veridiana Pessoa Miyajima
EPI_ISL_8721898	PA CAPELA	Instituto Butantan	Antonio Jorge Martins; Claudia Renata dos Santos Barros; David Schlesinger; Debora Botequio Moretti; Dimas Tadeu Covas; Elaine Cristina Marqueze; Elaine Vieira Santos; Evandra Strazza Rodrigues; Heidge Fukumasu; Jayme Augusto de Souza-Neto; José Salvatore Leister Patané; Luiz Alcantara; Luiz Lehmann Coutinho; Maria Carolina Elias; Maurício Lacerda Nogueira; Rafael dos Santos Bezerra; Raul Machado Neto; Rejane Maria Tommasini Grotto; Ricardo Haddad; Sandra Coccuzzo Sampaio Vessoni; Simone Kashima; Svetoslav Nanev Slavov; Vincent Louis Viala
EPI_ISL_8149410	PRADOPOLIS - PRONTO ATENDIMENTO MUNICIPAL WALDEMAR BALATORE - LABMÓVEL	Instituto Butantan	Antonio Jorge Martins; Claudia Renata dos Santos Barros; David Schlesinger; Debora Botequio Moretti; Dimas Tadeu Covas; Elaine Cristina Marqueze; Elaine Vieira Santos; Evandra Strazza Rodrigues; Heidge Fukumasu; Jayme Augusto de Souza-Neto; José Salvatore Leister Patané; Luiz Alcantara; Luiz Lehmann Coutinho; Maria Carolina Elias; Maurício Lacerda Nogueira; Rafael dos Santos Bezerra; Raul Machado Neto; Rejane Maria Tommasini Grotto; Ricardo Haddad; Sandra Coccuzzo Sampaio Vessoni; Simone Kashima; Svetoslav Nanev Slavov; Vincent Louis Viala
EPI_ISL_8353522	PREFEITURA MUNICIPAL DE TAQUARITINGA - PRUDENTE DE MORAES - PCR	Instituto Butantan	Antonio Jorge Martins; Claudia Renata dos Santos Barros; David Schlesinger; Debora Botequio Moretti; Dimas Tadeu Covas; Elaine Cristina Marqueze; Elaine Vieira Santos; Evandra Strazza Rodrigues; Heidge Fukumasu; Jayme Augusto de Souza-Neto; José Salvatore Leister Patané; Luiz Alcantara; Luiz Lehmann Coutinho; Maria Carolina Elias; Maurício Lacerda Nogueira; Rafael dos Santos Bezerra; Raul Machado Neto; Rejane Maria Tommasini Grotto; Ricardo Haddad; Sandra Coccuzzo Sampaio Vessoni; Simone Kashima; Svetoslav Nanev Slavov; Vincent Louis Viala
EPI_ISL_8353571	PRONTO ATENDIMENTO DE ARACARIGUAMA	Instituto Butantan	Antonio Jorge Martins; Claudia Renata dos Santos Barros; David Schlesinger; Debora Botequio Moretti; Dimas Tadeu Covas; Elaine Cristina Marqueze; Elaine Vieira Santos; Evandra Strazza Rodrigues; Heidge Fukumasu; Jayme Augusto de Souza-Neto; José Salvatore Leister Patané; Luiz Alcantara; Luiz Lehmann Coutinho; Maria Carolina Elias; Maurício Lacerda Nogueira; Rafael dos Santos Bezerra; Raul Machado Neto; Rejane Maria Tommasini Grotto; Ricardo Haddad; Sandra Coccuzzo Sampaio Vessoni; Simone Kashima; Svetoslav Nanev Slavov; Vincent Louis Viala
EPI_ISL_7898477, EPI_ISL_7898479	PRONTO ATENDIMENTO MUNICIPAL WALDEMAR BALATORE	Instituto Butantan	Antonio Jorge Martins; Claudia Renata dos Santos Barros; David Schlesinger; Debora Botequio Moretti; Dimas Tadeu Covas; Elaine Cristina Marqueze; Elaine Vieira Santos; Evandra Strazza Rodrigues; Heidge Fukumasu; Jayme Augusto de Souza-Neto; José Salvatore Leister Patané; Luiz Alcantara; Luiz Lehmann Coutinho; Maria Carolina Elias; Maurício Lacerda Nogueira; Rafael dos Santos Bezerra; Raul Machado Neto; Rejane Maria Tommasini Grotto; Ricardo Haddad; Sandra Coccuzzo Sampaio Vessoni; Simone Kashima; Svetoslav Nanev Slavov; Vincent Louis Viala
EPI_ISL_8721411, EPI_ISL_8721538, EPI_ISL_8721796, EPI_ISL_8721838	PRONTO ATENDIMENTO SAO JOSE	Instituto Butantan	Antonio Jorge Martins; Claudia Renata dos Santos Barros; David Schlesinger; Debora Botequio Moretti; Dimas Tadeu Covas; Elaine Cristina Marqueze; Elaine Vieira Santos; Evandra Strazza Rodrigues; Heidge Fukumasu; Jayme Augusto de Souza-Neto; José Salvatore Leister Patané; Luiz Alcantara; Luiz Lehmann Coutinho; Maria Carolina Elias; Maurício Lacerda Nogueira; Rafael dos Santos Bezerra; Raul Machado Neto; Rejane Maria Tommasini Grotto; Ricardo Haddad; Sandra Coccuzzo Sampaio Vessoni; Simone Kashima; Svetoslav Nanev Slavov; Vincent Louis Viala
EPI_ISL_8353525	PRONTO SOCORRO DE JARDINOPOLIS	Instituto Butantan	Antonio Jorge Martins; Claudia Renata dos Santos Barros; David Schlesinger; Debora Botequio Moretti; Dimas Tadeu Covas; Elaine Cristina Marqueze; Elaine Vieira Santos; Evandra Strazza Rodrigues; Heidge Fukumasu; Jayme Augusto de Souza-Neto; José Salvatore Leister Patané; Luiz Alcantara; Luiz Lehmann Coutinho; Maria Carolina Elias; Maurício Lacerda Nogueira; Rafael dos Santos Bezerra; Raul Machado Neto; Rejane Maria Tommasini Grotto; Ricardo Haddad; Sandra Coccuzzo Sampaio Vessoni; Simone Kashima; Svetoslav Nanev Slavov; Vincent Louis Viala
EPI_ISL_8353586	PRONTO SOCORRO DE MONTE ALTO	Instituto Butantan	Antonio Jorge Martins; Claudia Renata dos Santos Barros; David Schlesinger; Debora Botequio Moretti; Dimas Tadeu Covas; Elaine Cristina Marqueze; Elaine Vieira Santos; Evandra Strazza Rodrigues; Heidge Fukumasu; Jayme Augusto de Souza-Neto; José Salvatore Leister Patané; Luiz Alcantara; Luiz Lehmann Coutinho; Maria Carolina Elias; Maurício Lacerda Nogueira; Rafael dos Santos Bezerra; Raul Machado Neto; Rejane Maria Tommasini Grotto; Ricardo Haddad; Sandra Coccuzzo Sampaio Vessoni; Simone Kashima; Svetoslav Nanev Slavov; Vincent Louis Viala
EPI_ISL_8465109, EPI_ISL_8721231, EPI_ISL_8721966	PRONTO SOCORRO MUNICIPAL DE ARACATUBA AIDA VANZO DOLCE	Instituto Butantan	Antonio Jorge Martins; Claudia Renata dos Santos Barros; David Schlesinger; Debora Botequio Moretti; Dimas Tadeu Covas; Elaine Cristina Marqueze; Elaine Vieira Santos; Evandra Strazza Rodrigues; Heidge Fukumasu; Jayme Augusto de Souza-Neto; José Salvatore Leister Patané; Luiz Alcantara; Luiz Lehmann Coutinho; Maria Carolina Elias; Maurício Lacerda Nogueira; Rafael dos Santos Bezerra; Raul Machado Neto; Rejane Maria Tommasini Grotto; Ricardo Haddad; Sandra Coccuzzo Sampaio Vessoni; Simone Kashima; Svetoslav Nanev Slavov; Vincent Louis Viala
EPI_ISL_8721325, EPI_ISL_8721353, EPI_ISL_8721442, EPI_ISL_8721718, EPI_ISL_8721722, EPI_ISL_8721960, EPI_ISL_8721991			
see above	PRONTO SOCORRO MUNICIPAL DE TAUBATE	Instituto Butantan	Antonio Jorge Martins; Claudia Renata dos Santos Barros; David Schlesinger; Debora Botequio Moretti; Dimas Tadeu Covas; Elaine Cristina Marqueze; Elaine Vieira Santos; Evandra Strazza Rodrigues; Heidge Fukumasu; Jayme Augusto de Souza-Neto; José Salvatore Leister Patané; Luiz Alcantara; Luiz Lehmann Coutinho; Maria Carolina Elias; Maurício Lacerda Nogueira; Rafael dos Santos Bezerra; Raul Machado Neto; Rejane Maria Tommasini Grotto; Ricardo Haddad; Sandra Coccuzzo Sampaio Vessoni; Simone Kashima; Svetoslav Nanev Slavov; Vincent Louis Viala
EPI_ISL_8721244, EPI_ISL_8721386, EPI_ISL_8721750, EPI_ISL_8721820	PS E MATERNIDADE NAIR FONSECA LEITAO ARANTES	Instituto Butantan	Antonio Jorge Martins; Claudia Renata dos Santos Barros; David Schlesinger; Debora Botequio Moretti; Dimas Tadeu Covas; Elaine Cristina Marqueze; Elaine Vieira Santos; Evandra Strazza Rodrigues; Heidge Fukumasu; Jayme Augusto de Souza-Neto; José Salvatore Leister Patané; Luiz Alcantara; Luiz Lehmann Coutinho; Maria Carolina Elias; Maurício Lacerda Nogueira; Rafael dos Santos Bezerra; Raul Machado Neto; Rejane Maria Tommasini Grotto; Ricardo Haddad; Sandra Coccuzzo Sampaio Vessoni; Simone Kashima; Svetoslav Nanev Slavov; Vincent Louis Viala
EPI_ISL_8353607, EPI_ISL_8721352, EPI_ISL_8721417, EPI_ISL_8721566	PSF DR ANTONIO PIRES DE ALMEIDA PORTO FELIZ	Instituto Butantan	Antonio Jorge Martins; Claudia Renata dos Santos Barros; David Schlesinger; Debora Botequio Moretti; Dimas Tadeu Covas; Elaine Cristina Marqueze; Elaine Vieira Santos; Evandra Strazza Rodrigues; Heidge Fukumasu; Jayme Augusto de Souza-Neto; José Salvatore Leister Patané; Luiz Alcantara; Luiz Lehmann Coutinho; Maria Carolina Elias; Maurício Lacerda Nogueira; Rafael dos Santos Bezerra; Raul Machado Neto; Rejane Maria Tommasini Grotto; Ricardo Haddad; Sandra Coccuzzo Sampaio Vessoni; Simone Kashima; Svetoslav Nanev Slavov; Vincent Louis Viala
EPI_ISL_8149379, EPI_ISL_8149381, EPI_ISL_8149413, EPI_ISL_8149453	RIBEIRAO PRETO - UPA 13 DE MAIO – DR LUIS ATILIO LOSI VIANA	Instituto Butantan	Antonio Jorge Martins; Claudia Renata dos Santos Barros; David Schlesinger; Debora Botequio Moretti; Dimas Tadeu Covas; Elaine Cristina Marqueze; Elaine Vieira Santos; Evandra Strazza Rodrigues; Heidge Fukumasu; Jayme Augusto de Souza-Neto; José Salvatore Leister Patané; Luiz Alcantara; Luiz Lehmann Coutinho; Maria Carolina Elias; Maurício Lacerda Nogueira; Rafael dos Santos Bezerra; Raul Machado Neto; Rejane Maria Tommasini Grotto; Ricardo Haddad; Sandra Coccuzzo Sampaio Vessoni; Simone Kashima; Svetoslav Nanev Slavov; Vincent Louis Viala
EPI_ISL_8149414	RIBEIRAO PRETO - UPA NORTE – DR NELSON MANDELA	Instituto Butantan	Antonio Jorge Martins; Claudia Renata dos Santos Barros; David Schlesinger; Debora Botequio Moretti; Dimas Tadeu Covas; Elaine Cristina Marqueze; Elaine Vieira Santos; Evandra Strazza Rodrigues; Heidge Fukumasu; Jayme Augusto de Souza-Neto; José Salvatore Leister Patané; Luiz Alcantara; Luiz Lehmann Coutinho; Maria Carolina Elias; Maurício Lacerda Nogueira; Rafael dos Santos Bezerra; Raul Machado Neto; Rejane Maria Tommasini Grotto; Ricardo Haddad; Sandra Coccuzzo Sampaio Vessoni; Simone Kashima; Svetoslav Nanev Slavov; Vincent Louis Viala
EPI_ISL_8149403	RIBEIRAO PRETO - UPA SUL	Instituto Butantan	Antonio Jorge Martins; Claudia Renata dos Santos Barros; David Schlesinger; Debora Botequio Moretti; Dimas Tadeu Covas; Elaine Cristina Marqueze; Elaine Vieira Santos; Evandra Strazza Rodrigues; Heidge Fukumasu; Jayme Augusto de Souza-Neto; José Salvatore Leister Patané; Luiz Alcantara; Luiz Lehmann Coutinho; Maria Carolina Elias; Maurício Lacerda Nogueira; Rafael dos Santos Bezerra; Raul Machado Neto; Rejane Maria Tommasini Grotto; Ricardo Haddad; Sandra Coccuzzo Sampaio Vessoni; Simone Kashima; Svetoslav Nanev Slavov; Vincent Louis Viala
EPI_ISL_8721276, EPI_ISL_8721334, EPI_ISL_8721477, EPI_ISL_8721486, EPI_ISL_8721818, EPI_ISL_8721819, EPI_ISL_8721868, EPI_ISL_8721883, EPI_ISL_8721908, EPI_ISL_8721918, EPI_ISL_8721919, EPI_ISL_8721920, EPI_ISL_8721934, EPI_ISL_8721963			
see above	SANTA CASA DE ATIBAIA PRO SAUDE	Instituto Butantan	Antonio Jorge Martins; Claudia Renata dos Santos Barros; David Schlesinger; Debora Botequio Moretti; Dimas Tadeu Covas; Elaine Cristina Marqueze; Elaine Vieira Santos; Evandra Strazza Rodrigues; Heidge Fukumasu; Jayme Augusto de Souza-Neto; José Salvatore Leister Patané; Luiz Alcantara; Luiz Lehmann Coutinho; Maria Carolina Elias; Maurício Lacerda Nogueira; Rafael dos Santos Bezerra; Raul Machado Neto; Rejane Maria Tommasini Grotto; Ricardo Haddad; Sandra Coccuzzo Sampaio Vessoni; Simone Kashima; Svetoslav Nanev Slavov; Vincent Louis Viala
EPI_ISL_8353533	SANTA CASA DE CRAVINHOS	Instituto Butantan	Antonio Jorge Martins; Claudia Renata dos Santos Barros; David Schlesinger; Debora Botequio Moretti; Dimas Tadeu Covas; Elaine Cristina Marqueze; Elaine Vieira Santos; Evandra Strazza Rodrigues; Heidge Fukumasu; Jayme Augusto de Souza-Neto; José Salvatore Leister Patané; Luiz Alcantara; Luiz Lehmann Coutinho; Maria Carolina Elias; Maurício Lacerda Nogueira; Rafael dos Santos Bezerra; Raul Machado Neto; Rejane Maria Tommasini Grotto; Ricardo Haddad; Sandra Coccuzzo Sampaio Vessoni; Simone Kashima; Svetoslav Nanev Slavov; Vincent Louis Viala
EPI_ISL_8721374, EPI_ISL_8721385, EPI_ISL_8721408, EPI_ISL_8721430, EPI_ISL_8721622, EPI_ISL_8721716, EPI_ISL_8721916, EPI_ISL_8721979			
see above	SANTA CASA DE GUARAREMA	Instituto Butantan	Antonio Jorge Martins; Claudia Renata dos Santos Barros; David Schlesinger; Debora Botequio Moretti; Dimas Tadeu Covas; Elaine Cristina Marqueze; Elaine Vieira Santos; Evandra Strazza Rodrigues; Heidge Fukumasu; Jayme Augusto de Souza-Neto; José Salvatore Leister Patané; Luiz Alcantara; Luiz Lehmann Coutinho; Maria Carolina Elias; Maurício Lacerda Nogueira; Rafael dos Santos Bezerra; Raul Machado Neto; Rejane Maria Tommasini Grotto; Ricardo Haddad; Sandra Coccuzzo Sampaio Vessoni; Simone Kashima; Svetoslav Nanev Slavov; Vincent Louis Viala
EPI_ISL_8721445	SANTA CASA DE MISERICORDIA DE BRAGANCA PAULISTA	Instituto Butantan	Antonio Jorge Martins; Claudia Renata dos Santos Barros; David Schlesinger; Debora Botequio Moretti; Dimas Tadeu Covas; Elaine Cristina Marqueze; Elaine Vieira Santos; Evandra Strazza Rodrigues; Heidge Fukumasu; Jayme Augusto de Souza-Neto; José Salvatore Leister Patané; Luiz Alcantara; Luiz Lehmann Coutinho; Maria Carolina Elias; Maurício Lacerda Nogueira; Rafael dos Santos Bezerra; Raul Machado Neto; Rejane Maria Tommasini Grotto; Ricardo Haddad; Sandra Coccuzzo Sampaio Vessoni; Simone Kashima; Svetoslav Nanev Slavov; Vincent Louis Viala
EPI_ISL_8721431, EPI_ISL_8721746	SANTA CASA DE MISERICORDIA DE UBATUBA	Instituto Butantan	Antonio Jorge Martins; Claudia Renata dos Santos Barros; David Schlesinger; Debora Botequio Moretti; Dimas Tadeu Covas; Elaine Cristina Marqueze; Elaine Vieira Santos; Evandra Strazza Rodrigues; Heidge Fukumasu; Jayme Augusto de Souza-Neto; José Salvatore Leister Patané; Luiz Alcantara; Luiz Lehmann Coutinho; Maria Carolina Elias; Maurício Lacerda Nogueira; Rafael dos Santos Bezerra; Raul Machado Neto; Rejane Maria Tommasini Grotto; Ricardo Haddad; Sandra Coccuzzo Sampaio Vessoni; Simone Kashima; Svetoslav Nanev Slavov; Vincent Louis Viala
EPI_ISL_8465106	SANTA CASA DE TAQUARITUBA	Instituto Butantan	Antonio Jorge Martins; Claudia Renata dos Santos Barros; David Schlesinger; Debora Botequio Moretti; Dimas Tadeu Covas; Elaine Cristina Marqueze; Elaine Vieira Santos; Evandra Strazza Rodrigues; Heidge Fukumasu; Jayme Augusto de Souza-Neto; José Salvatore Leister Patané; Luiz Alcantara; Luiz Lehmann Coutinho; Maria Carolina Elias; Maurício Lacerda Nogueira; Rafael dos Santos Bezerra; Raul Machado Neto; Rejane Maria Tommasini Grotto; Ricardo Haddad; Sandra Coccuzzo Sampaio Vessoni; Simone Kashima; Svetoslav Nanev Slavov; Vincent Louis Viala
EPI_ISL_8621414	SECRETARIA MUNICIPAL DA SAUDE DE RUSSAS	Analytical Competence Molecular Epidemiology Lab/ACME, Oswaldo Cruz Foundation, Ceara (FIOCRUZ CE)	Carlos Leonardo de Aragao Araujo; Cleber Furtado Aksenen; Fabio Miyajima; Fernando Braga Stehling; Igor Oliveira Duarte & Nicole Silva França on behalf of COVID-19 FIOCRUZ Genomic Network; Jamille Maria Mendes Bezerra; Joaquim Cesar do Nascimento Sousa Junior; Pedro Miguel Carneiro Jeronimo; Suzana Porto Almeida; Thais Ferreira de Oliveira; Thais de Oliveira Costa; Ticiane Cavalcante de Souza; Veridiana Pessoa Miyajima
EPI_ISL_8721392, EPI_ISL_8721828	SECRETARIA MUNICIPAL DE BASTOS	Instituto Butantan	Antonio Jorge Martins; Claudia Renata dos Santos Barros; David Schlesinger; Debora Botequio Moretti; Dimas Tadeu Covas; Elaine Cristina Marqueze; Elaine Vieira Santos; Evandra Strazza Rodrigues; Heidge Fukumasu; Jayme Augusto de Souza-Neto; José Salvatore Leister Patané; Luiz Alcantara; Luiz Lehmann Coutinho; Maria Carolina Elias; Maurício Lacerda Nogueira; Rafael dos Santos Bezerra; Raul Machado Neto; Rejane Maria Tommasini Grotto; Ricardo Haddad; Sandra Coccuzzo Sampaio Vessoni; Simone Kashima; Svetoslav Nanev Slavov; Vincent Louis Viala
EPI_ISL_8353527, EPI_ISL_8353584, EPI_ISL_8353585	SECRETARIA MUNICIPAL DE SAUDE	Instituto Butantan	Antonio Jorge Martins; Claudia Renata dos Santos Barros; David Schlesinger; Debora Botequio Moretti; Dimas Tadeu Covas; Elaine Cristina Marqueze; Elaine Vieira Santos; Evandra Strazza Rodrigues; Heidge Fukumasu; Jayme Augusto de Souza-Neto; José Salvatore Leister Patané; Luiz Alcantara; Luiz Lehmann Coutinho; Maria Carolina Elias; Maurício Lacerda Nogueira; Rafael dos Santos Bezerra; Raul Machado Neto; Rejane Maria Tommasini Grotto; Ricardo Haddad; Sandra Coccuzzo Sampaio Vessoni; Simone Kashima; Svetoslav Nanev Slavov; Vincent Louis Viala
EPI_ISL_8721674	SECRETARIA MUNICIPAL DE SAUDE DE LINDOIA	Instituto Butantan	Antonio Jorge Martins; Claudia Renata dos Santos Barros; David Schlesinger; Debora Botequio Moretti; Dimas Tadeu Covas; Elaine Cristina Marqueze; Elaine Vieira Santos; Evandra Strazza Rodrigues; Heidge Fukumasu; Jayme Augusto de Souza-Neto; José Salvatore Leister Patané; Luiz Alcantara; Luiz Lehmann Coutinho; Maria Carolina Elias; Maurício Lacerda Nogueira; Rafael dos Santos Bezerra; Raul Machado Neto; Rejane Maria Tommasini Grotto; Ricardo Haddad; Sandra Coccuzzo Sampaio Vessoni; Simone Kashima; Svetoslav Nanev Slavov; Vincent Louis Viala
EPI_ISL_8721695, EPI_ISL_8721766, EPI_ISL_8721865	SECRETARIA MUNICIPAL DE SAUDE DE PIRACAIA	Instituto Butantan	Antonio Jorge Martins; Claudia Renata dos Santos Barros; David Schlesinger; Debora Botequio Moretti; Dimas Tadeu Covas; Elaine Cristina Marqueze; Elaine Vieira Santos; Evandra Strazza Rodrigues; Heidge Fukumasu; Jayme Augusto de Souza-Neto; José Salvatore Leister Patané; Luiz Alcantara; Luiz Lehmann Coutinho; Maria Carolina Elias; Maurício Lacerda Nogueira; Rafael dos Santos Bezerra; Raul Machado Neto; Rejane Maria Tommasini Grotto; Ricardo Haddad; Sandra Coccuzzo Sampaio Vessoni; Simone Kashima; Svetoslav Nanev Slavov; Vincent Louis Viala
EPI_ISL_8184834, EPI_ISL_8353540, EPI_ISL_8353548, EPI_ISL_8721250, EPI_ISL_8721395, EPI_ISL_8721396, EPI_ISL_8721398, EPI_ISL_8721415, EPI_ISL_8721443, EPI_ISL_8721453, EPI_ISL_8721454, EPI_ISL_8721475, EPI_ISL_8721505, EPI_ISL_8721506, EPI_ISL_8721672, EPI_ISL_8721673, EPI_ISL_8721745, EPI_ISL_8721749, EPI_ISL_8721794, EPI_ISL_8721817, EPI_ISL_8721893, EPI_ISL_8721912, EPI_ISL_8721917, EPI_ISL_8721941, EPI_ISL_8721985			
see above	SECRETARIA MUNICIPAL DE SAUDE SOROCABA	Instituto Butantan	Antonio Jorge Martins; Claudia Renata dos Santos Barros; David Schlesinger; Debora Botequio Moretti; Dimas Tadeu Covas; Elaine Cristina Marqueze; Elaine Vieira Santos; Evandra Strazza Rodrigues; Heidge Fukumasu; Jayme Augusto de Souza-Neto; José Salvatore Leister Patané; Luiz Alcantara; Luiz Lehmann Coutinho; Maria Carolina Elias; Maurício Lacerda Nogueira; Rafael dos Santos Bezerra; Raul Machado Neto; Rejane Maria Tommasini Grotto; Ricardo Haddad; Sandra Coccuzzo Sampaio Vessoni; Simone Kashima; Svetoslav Nanev Slavov; Vincent Louis Viala

EPI_ISL_8149382, EPI_ISL_8149454	SERTAOZINHO - UPA DR PEDRO THOME FRANCISCO DOS REIS	Instituto Butantan	Antonio Jorge Martins; Claudia Renata dos Santos Barros; David Schlesinger; Debora Botequio Moretti; Dimas Tadeu Covas; Elaine Cristina Marqueze; Elaine Vieira Santos; Evandra Strazza Rodrigues; Heidge Fukumasu; Jayme Augusto de Souza-Neto; José Salvatore Leister Patané; Luiz Alcantara; Luiz Lehmann Coutinho; Maria Carolina Elias; Maurício Lacerda Nogueira; Rafael dos Santos Bezerra; Raul Machado Neto; Rejane Maria Tommasini Grotto; Ricardo Haddad; Sandra Coccuzzo Sampaio Vessoni; Simone Kashima; Svetoslav Nanev Slavov; Vincent Louis Viala
EPI_ISL_8721254, EPI_ISL_8721347, EPI_ISL_8721441, EPI_ISL_8721561, EPI_ISL_8721736, EPI_ISL_8721761, EPI_ISL_8721813, EPI_ISL_8721814, EPI_ISL_8721859, EPI_ISL_8721860, EPI_ISL_8721952, EPI_ISL_8721970			
see above	SMS SP SECRETARIA MUNICIPAL DE SAUDE SAO JOAO DA BOA VISTA	Instituto Butantan	Antonio Jorge Martins; Claudia Renata dos Santos Barros; David Schlesinger; Debora Botequio Moretti; Dimas Tadeu Covas; Elaine Cristina Marqueze; Elaine Vieira Santos; Evandra Strazza Rodrigues; Heidge Fukumasu; Jayme Augusto de Souza-Neto; José Salvatore Leister Patané; Luiz Alcantara; Luiz Lehmann Coutinho; Maria Carolina Elias; Maurício Lacerda Nogueira; Rafael dos Santos Bezerra; Raul Machado Neto; Rejane Maria Tommasini Grotto; Ricardo Haddad; Sandra Coccuzzo Sampaio Vessoni; Simone Kashima; Svetoslav Nanev Slavov; Vincent Louis Viala
EPI_ISL_8353583	UBDS- DR MARCO A SAHAO - VILA VIRGINIA	Instituto Butantan	Antonio Jorge Martins; Claudia Renata dos Santos Barros; David Schlesinger; Debora Botequio Moretti; Dimas Tadeu Covas; Elaine Cristina Marqueze; Elaine Vieira Santos; Evandra Strazza Rodrigues; Heidge Fukumasu; Jayme Augusto de Souza-Neto; José Salvatore Leister Patané; Luiz Alcantara; Luiz Lehmann Coutinho; Maria Carolina Elias; Maurício Lacerda Nogueira; Rafael dos Santos Bezerra; Raul Machado Neto; Rejane Maria Tommasini Grotto; Ricardo Haddad; Sandra Coccuzzo Sampaio Vessoni; Simone Kashima; Svetoslav Nanev Slavov; Vincent Louis Viala
EPI_ISL_8465103	UBS 01 VILA BANDEIRANTES BIRIGUI	Instituto Butantan	Antonio Jorge Martins; Claudia Renata dos Santos Barros; David Schlesinger; Debora Botequio Moretti; Dimas Tadeu Covas; Elaine Cristina Marqueze; Elaine Vieira Santos; Evandra Strazza Rodrigues; Heidge Fukumasu; Jayme Augusto de Souza-Neto; José Salvatore Leister Patané; Luiz Alcantara; Luiz Lehmann Coutinho; Maria Carolina Elias; Maurício Lacerda Nogueira; Rafael dos Santos Bezerra; Raul Machado Neto; Rejane Maria Tommasini Grotto; Ricardo Haddad; Sandra Coccuzzo Sampaio Vessoni; Simone Kashima; Svetoslav Nanev Slavov; Vincent Louis Viala
EPI_ISL_8721708, EPI_ISL_8721953	UBS CASTELO BRANCO	Instituto Butantan	Antonio Jorge Martins; Claudia Renata dos Santos Barros; David Schlesinger; Debora Botequio Moretti; Dimas Tadeu Covas; Elaine Cristina Marqueze; Elaine Vieira Santos; Evandra Strazza Rodrigues; Heidge Fukumasu; Jayme Augusto de Souza-Neto; José Salvatore Leister Patané; Luiz Alcantara; Luiz Lehmann Coutinho; Maria Carolina Elias; Maurício Lacerda Nogueira; Rafael dos Santos Bezerra; Raul Machado Neto; Rejane Maria Tommasini Grotto; Ricardo Haddad; Sandra Coccuzzo Sampaio Vessoni; Simone Kashima; Svetoslav Nanev Slavov; Vincent Louis Viala
EPI_ISL_8721675	UBS DE SANTA SALETE	Instituto Butantan	Antonio Jorge Martins; Claudia Renata dos Santos Barros; David Schlesinger; Debora Botequio Moretti; Dimas Tadeu Covas; Elaine Cristina Marqueze; Elaine Vieira Santos; Evandra Strazza Rodrigues; Heidge Fukumasu; Jayme Augusto de Souza-Neto; José Salvatore Leister Patané; Luiz Alcantara; Luiz Lehmann Coutinho; Maria Carolina Elias; Maurício Lacerda Nogueira; Rafael dos Santos Bezerra; Raul Machado Neto; Rejane Maria Tommasini Grotto; Ricardo Haddad; Sandra Coccuzzo Sampaio Vessoni; Simone Kashima; Svetoslav Nanev Slavov; Vincent Louis Viala
EPI_ISL_8353596	UBS DO POVO DE SANTA ROSA DE VITERBO	Instituto Butantan	Antonio Jorge Martins; Claudia Renata dos Santos Barros; David Schlesinger; Debora Botequio Moretti; Dimas Tadeu Covas; Elaine Cristina Marqueze; Elaine Vieira Santos; Evandra Strazza Rodrigues; Heidge Fukumasu; Jayme Augusto de Souza-Neto; José Salvatore Leister Patané; Luiz Alcantara; Luiz Lehmann Coutinho; Maria Carolina Elias; Maurício Lacerda Nogueira; Rafael dos Santos Bezerra; Raul Machado Neto; Rejane Maria Tommasini Grotto; Ricardo Haddad; Sandra Coccuzzo Sampaio Vessoni; Simone Kashima; Svetoslav Nanev Slavov; Vincent Louis Viala
EPI_ISL_8465104, EPI_ISL_8465105, EPI_ISL_8721424	UBS DR ALFREDO DANTAS DE SOUZA UMUARAMA	Instituto Butantan	Antonio Jorge Martins; Claudia Renata dos Santos Barros; David Schlesinger; Debora Botequio Moretti; Dimas Tadeu Covas; Elaine Cristina Marqueze; Elaine Vieira Santos; Evandra Strazza Rodrigues; Heidge Fukumasu; Jayme Augusto de Souza-Neto; José Salvatore Leister Patané; Luiz Alcantara; Luiz Lehmann Coutinho; Maria Carolina Elias; Maurício Lacerda Nogueira; Rafael dos Santos Bezerra; Raul Machado Neto; Rejane Maria Tommasini Grotto; Ricardo Haddad; Sandra Coccuzzo Sampaio Vessoni; Simone Kashima; Svetoslav Nanev Slavov; Vincent Louis Viala
EPI_ISL_8721509, EPI_ISL_8721867	UBS EUFRASIO PEREIRA COSTA	Instituto Butantan	Antonio Jorge Martins; Claudia Renata dos Santos Barros; David Schlesinger; Debora Botequio Moretti; Dimas Tadeu Covas; Elaine Cristina Marqueze; Elaine Vieira Santos; Evandra Strazza Rodrigues; Heidge Fukumasu; Jayme Augusto de Souza-Neto; José Salvatore Leister Patané; Luiz Alcantara; Luiz Lehmann Coutinho; Maria Carolina Elias; Maurício Lacerda Nogueira; Rafael dos Santos Bezerra; Raul Machado Neto; Rejane Maria Tommasini Grotto; Ricardo Haddad; Sandra Coccuzzo Sampaio Vessoni; Simone Kashima; Svetoslav Nanev Slavov; Vincent Louis Viala
EPI_ISL_8184835	UBS JANDIRA MASSUR V GUMERCINDO	Instituto Butantan	Antonio Jorge Martins; Claudia Renata dos Santos Barros; David Schlesinger; Debora Botequio Moretti; Dimas Tadeu Covas; Elaine Cristina Marqueze; Elaine Vieira Santos; Evandra Strazza Rodrigues; Heidge Fukumasu; Jayme Augusto de Souza-Neto; José Salvatore Leister Patané; Luiz Alcantara; Luiz Lehmann Coutinho; Maria Carolina Elias; Maurício Lacerda Nogueira; Rafael dos Santos Bezerra; Raul Machado Neto; Rejane Maria Tommasini Grotto; Ricardo Haddad; Sandra Coccuzzo Sampaio Vessoni; Simone Kashima; Svetoslav Nanev Slavov; Vincent Louis Viala
EPI_ISL_8353547	UBS JARDIM NOVA CUMBICA	Instituto Butantan	Antonio Jorge Martins; Claudia Renata dos Santos Barros; David Schlesinger; Debora Botequio Moretti; Dimas Tadeu Covas; Elaine Cristina Marqueze; Elaine Vieira Santos; Evandra Strazza Rodrigues; Heidge Fukumasu; Jayme Augusto de Souza-Neto; José Salvatore Leister Patané; Luiz Alcantara; Luiz Lehmann Coutinho; Maria Carolina Elias; Maurício Lacerda Nogueira; Rafael dos Santos Bezerra; Raul Machado Neto; Rejane Maria Tommasini Grotto; Ricardo Haddad; Sandra Coccuzzo Sampaio Vessoni; Simone Kashima; Svetoslav Nanev Slavov; Vincent Louis Viala
EPI_ISL_8184838	UBS JARDIM VILA GALVAO	Instituto Butantan	Antonio Jorge Martins; Claudia Renata dos Santos Barros; David Schlesinger; Debora Botequio Moretti; Dimas Tadeu Covas; Elaine Cristina Marqueze; Elaine Vieira Santos; Evandra Strazza Rodrigues; Heidge Fukumasu; Jayme Augusto de Souza-Neto; José Salvatore Leister Patané; Luiz Alcantara; Luiz Lehmann Coutinho; Maria Carolina Elias; Maurício Lacerda Nogueira; Rafael dos Santos Bezerra; Raul Machado Neto; Rejane Maria Tommasini Grotto; Ricardo Haddad; Sandra Coccuzzo Sampaio Vessoni; Simone Kashima; Svetoslav Nanev Slavov; Vincent Louis Viala
EPI_ISL_8353543	UBS JD DONALISIO	Instituto Butantan	Antonio Jorge Martins; Claudia Renata dos Santos Barros; David Schlesinger; Debora Botequio Moretti; Dimas Tadeu Covas; Elaine Cristina Marqueze; Elaine Vieira Santos; Evandra Strazza Rodrigues; Heidge Fukumasu; Jayme Augusto de Souza-Neto; José Salvatore Leister Patané; Luiz Alcantara; Luiz Lehmann Coutinho; Maria Carolina Elias; Maurício Lacerda Nogueira; Rafael dos Santos Bezerra; Raul Machado Neto; Rejane Maria Tommasini Grotto; Ricardo Haddad; Sandra Coccuzzo Sampaio Vessoni; Simone Kashima; Svetoslav Nanev Slavov; Vincent Louis Viala
EPI_ISL_8721931, EPI_ISL_8721954, EPI_ISL_8721955, EPI_ISL_8721988	UBS NOVA MARILIA	Instituto Butantan	Antonio Jorge Martins; Claudia Renata dos Santos Barros; David Schlesinger; Debora Botequio Moretti; Dimas Tadeu Covas; Elaine Cristina Marqueze; Elaine Vieira Santos; Evandra Strazza Rodrigues; Heidge Fukumasu; Jayme Augusto de Souza-Neto; José Salvatore Leister Patané; Luiz Alcantara; Luiz Lehmann Coutinho; Maria Carolina Elias; Maurício Lacerda Nogueira; Rafael dos Santos Bezerra; Raul Machado Neto; Rejane Maria Tommasini Grotto; Ricardo Haddad; Sandra Coccuzzo Sampaio Vessoni; Simone Kashima; Svetoslav Nanev Slavov; Vincent Louis Viala
EPI_ISL_8721265	UBS PARQUE CECAP	Instituto Butantan	Antonio Jorge Martins; Claudia Renata dos Santos Barros; David Schlesinger; Debora Botequio Moretti; Dimas Tadeu Covas; Elaine Cristina Marqueze; Elaine Vieira Santos; Evandra Strazza Rodrigues; Heidge Fukumasu; Jayme Augusto de Souza-Neto; José Salvatore Leister Patané; Luiz Alcantara; Luiz Lehmann Coutinho; Maria Carolina Elias; Maurício Lacerda Nogueira; Rafael dos Santos Bezerra; Raul Machado Neto; Rejane Maria Tommasini Grotto; Ricardo Haddad; Sandra Coccuzzo Sampaio Vessoni; Simone Kashima; Svetoslav Nanev Slavov; Vincent Louis Viala
EPI_ISL_8721282, EPI_ISL_8721714, EPI_ISL_8722001	UBS PREF JOSE ERONIS BARBOSA DA SILVA	Instituto Butantan	Antonio Jorge Martins; Claudia Renata dos Santos Barros; David Schlesinger; Debora Botequio Moretti; Dimas Tadeu Covas; Elaine Cristina Marqueze; Elaine Vieira Santos; Evandra Strazza Rodrigues; Heidge Fukumasu; Jayme Augusto de Souza-Neto; José Salvatore Leister Patané; Luiz Alcantara; Luiz Lehmann Coutinho; Maria Carolina Elias; Maurício Lacerda Nogueira; Rafael dos Santos Bezerra; Raul Machado Neto; Rejane Maria Tommasini Grotto; Ricardo Haddad; Sandra Coccuzzo Sampaio Vessoni; Simone Kashima; Svetoslav Nanev Slavov; Vincent Louis Viala
EPI_ISL_8721959	UBS SALTO DE SAO JOSE	Instituto Butantan	Antonio Jorge Martins; Claudia Renata dos Santos Barros; David Schlesinger; Debora Botequio Moretti; Dimas Tadeu Covas; Elaine Cristina Marqueze; Elaine Vieira Santos; Evandra Strazza Rodrigues; Heidge Fukumasu; Jayme Augusto de Souza-Neto; José Salvatore Leister Patané; Luiz Alcantara; Luiz Lehmann Coutinho; Maria Carolina Elias; Maurício Lacerda Nogueira; Rafael dos Santos Bezerra; Raul Machado Neto; Rejane Maria Tommasini Grotto; Ricardo Haddad; Sandra Coccuzzo Sampaio Vessoni; Simone Kashima; Svetoslav Nanev Slavov; Vincent Louis Viala
EPI_ISL_8721306	UBS SALVADOR DE LEONE	Instituto Butantan	Antonio Jorge Martins; Claudia Renata dos Santos Barros; David Schlesinger; Debora Botequio Moretti; Dimas Tadeu Covas; Elaine Cristina Marqueze; Elaine Vieira Santos; Evandra Strazza Rodrigues; Heidge Fukumasu; Jayme Augusto de Souza-Neto; José Salvatore Leister Patané; Luiz Alcantara; Luiz Lehmann Coutinho; Maria Carolina Elias; Maurício Lacerda Nogueira; Rafael dos Santos Bezerra; Raul Machado Neto; Rejane Maria Tommasini Grotto; Ricardo Haddad; Sandra Coccuzzo Sampaio Vessoni; Simone Kashima; Svetoslav Nanev Slavov; Vincent Louis Viala
EPI_ISL_8721896	UBS V N SRA APARECIDA	Instituto Butantan	Antonio Jorge Martins; Claudia Renata dos Santos Barros; David Schlesinger; Debora Botequio Moretti; Dimas Tadeu Covas; Elaine Cristina Marqueze; Elaine Vieira Santos; Evandra Strazza Rodrigues; Heidge Fukumasu; Jayme Augusto de Souza-Neto; José Salvatore Leister Patané; Luiz Alcantara; Luiz Lehmann Coutinho; Maria Carolina Elias; Maurício Lacerda Nogueira; Rafael dos Santos Bezerra; Raul Machado Neto; Rejane Maria Tommasini Grotto; Ricardo Haddad; Sandra Coccuzzo Sampaio Vessoni; Simone Kashima; Svetoslav Nanev Slavov; Vincent Louis Viala
EPI_ISL_8353538	UBS VILA MANGALOT	Instituto Butantan	Antonio Jorge Martins; Claudia Renata dos Santos Barros; David Schlesinger; Debora Botequio Moretti; Dimas Tadeu Covas; Elaine Cristina Marqueze; Elaine Vieira Santos; Evandra Strazza Rodrigues; Heidge Fukumasu; Jayme Augusto de Souza-Neto; José Salvatore Leister Patané; Luiz Alcantara; Luiz Lehmann Coutinho; Maria Carolina Elias; Maurício Lacerda Nogueira; Rafael dos Santos Bezerra; Raul Machado Neto; Rejane Maria Tommasini Grotto; Ricardo Haddad; Sandra Coccuzzo Sampaio Vessoni; Simone Kashima; Svetoslav Nanev Slavov; Vincent Louis Viala
EPI_ISL_8721633	UNIDADE BASICA DE SAUDE DE TARUMA	Instituto Butantan	Antonio Jorge Martins; Claudia Renata dos Santos Barros; David Schlesinger; Debora Botequio Moretti; Dimas Tadeu Covas; Elaine Cristina Marqueze; Elaine Vieira Santos; Evandra Strazza Rodrigues; Heidge Fukumasu; Jayme Augusto de Souza-Neto; José Salvatore Leister Patané; Luiz Alcantara; Luiz Lehmann Coutinho; Maria Carolina Elias; Maurício Lacerda Nogueira; Rafael dos Santos Bezerra; Raul Machado Neto; Rejane Maria Tommasini Grotto; Ricardo Haddad; Sandra Coccuzzo Sampaio Vessoni; Simone Kashima; Svetoslav Nanev Slavov; Vincent Louis Viala
EPI_ISL_8721519	UNIDADE BASICA DE SAUDE II DE INDIANA	Instituto Butantan	Antonio Jorge Martins; Claudia Renata dos Santos Barros; David Schlesinger; Debora Botequio Moretti; Dimas Tadeu Covas; Elaine Cristina Marqueze; Elaine Vieira Santos; Evandra Strazza Rodrigues; Heidge Fukumasu; Jayme Augusto de Souza-Neto; José Salvatore Leister Patané; Luiz Alcantara; Luiz Lehmann Coutinho; Maria Carolina Elias; Maurício Lacerda Nogueira; Rafael dos Santos Bezerra; Raul Machado Neto; Rejane Maria Tommasini Grotto; Ricardo Haddad; Sandra Coccuzzo Sampaio Vessoni; Simone Kashima; Svetoslav Nanev Slavov; Vincent Louis Viala
EPI_ISL_8184839, EPI_ISL_8353608, EPI_ISL_8721679, EPI_ISL_8721719, EPI_ISL_8721866	UNIDADE DE INFECTOLOGIA WILLIAN ROCHA GUARUJA	Instituto Butantan	Antonio Jorge Martins; Claudia Renata dos Santos Barros; David Schlesinger; Debora Botequio Moretti; Dimas Tadeu Covas; Elaine Cristina Marqueze; Elaine Vieira Santos; Evandra Strazza Rodrigues; Heidge Fukumasu; Jayme Augusto de Souza-Neto; José Salvatore Leister Patané; Luiz Alcantara; Luiz Lehmann Coutinho; Maria Carolina Elias; Maurício Lacerda Nogueira; Rafael dos Santos Bezerra; Raul Machado Neto; Rejane Maria Tommasini Grotto; Ricardo Haddad; Sandra Coccuzzo Sampaio Vessoni; Simone Kashima; Svetoslav Nanev Slavov; Vincent Louis Viala
EPI_ISL_8721321, EPI_ISL_8721341, EPI_ISL_8721671, EPI_ISL_8721696	UNIDADE DE PRONTO ATENDIMENTO CARLOS LOURENCO	Instituto Butantan	Antonio Jorge Martins; Claudia Renata dos Santos Barros; David Schlesinger; Debora Botequio Moretti; Dimas Tadeu Covas; Elaine Cristina Marqueze; Elaine Vieira Santos; Evandra Strazza Rodrigues; Heidge Fukumasu; Jayme Augusto de Souza-Neto; José Salvatore Leister Patané; Luiz Alcantara; Luiz Lehmann Coutinho; Maria Carolina Elias; Maurício Lacerda Nogueira; Rafael dos Santos Bezerra; Raul Machado Neto; Rejane Maria Tommasini Grotto; Ricardo Haddad; Sandra Coccuzzo Sampaio Vessoni; Simone Kashima; Svetoslav Nanev Slavov; Vincent Louis Viala
EPI_ISL_8184836, EPI_ISL_8184837, EPI_ISL_8353524, EPI_ISL_8353545, EPI_ISL_8353546, EPI_ISL_8721229, EPI_ISL_8721275, EPI_ISL_8721290, EPI_ISL_8721324, EPI_ISL_8721340, EPI_ISL_8721393, EPI_ISL_8721394, EPI_ISL_8721400, EPI_ISL_8721452, EPI_ISL_8721474, EPI_ISL_8721490, EPI_ISL_8721491, EPI_ISL_8721503, EPI_ISL_8721504, EPI_ISL_8721512, EPI_ISL_8721525, EPI_ISL_8721585, EPI_ISL_8721603, EPI_ISL_8721635, EPI_ISL_8721636, EPI_ISL_8721656, EPI_ISL_8721678, EPI_ISL_8721680, EPI_ISL_8721692, EPI_ISL_8721711, EPI_ISL_8721713, EPI_ISL_8721715, EPI_ISL_8721738, EPI_ISL_8721739, EPI_ISL_8721741, EPI_ISL_8721791, EPI_ISL_8721862, EPI_ISL_8721889, EPI_ISL_8721913, EPI_ISL_8721914, EPI_ISL_8721915, EPI_ISL_8721957, EPI_ISL_8721958, EPI_ISL_8721976, EPI_ISL_8721984, EPI_ISL_8721986, EPI_ISL_8721989, EPI_ISL_8721994, EPI_ISL_8721997			
see above	UNIDADE DE PRONTO ATENDIMENTO CENTRAL DE CARAGUATATUBA	Instituto Butantan	Antonio Jorge Martins; Claudia Renata dos Santos Barros; David Schlesinger; Debora Botequio Moretti; Dimas Tadeu Covas; Elaine Cristina Marqueze; Elaine Vieira Santos; Evandra Strazza Rodrigues; Heidge Fukumasu; Jayme Augusto de Souza-Neto; José Salvatore Leister Patané; Luiz Alcantara; Luiz Lehmann Coutinho; Maria Carolina Elias; Maurício Lacerda Nogueira; Rafael dos Santos Bezerra; Raul Machado Neto; Rejane Maria Tommasini Grotto; Ricardo Haddad; Sandra Coccuzzo Sampaio Vessoni; Simone Kashima; Svetoslav Nanev Slavov; Vincent Louis Viala
EPI_ISL_8184731, EPI_ISL_8621411, EPI_ISL_8621412, EPI_ISL_8621413, EPI_ISL_8621415, EPI_ISL_8621416, EPI_ISL_8621417, EPI_ISL_8621418, EPI_ISL_8621419, EPI_ISL_8621420, EPI_ISL_8621421, EPI_ISL_8621422, EPI_ISL_8621423, EPI_ISL_8621424, EPI_ISL_8621425, EPI_ISL_8621426, EPI_ISL_8621427, EPI_ISL_8621428, EPI_ISL_8621429, EPI_ISL_8621430, EPI_ISL_8621431, EPI_ISL_8621432, EPI_ISL_8621433, EPI_ISL_8621434, EPI_ISL_8621435, EPI_ISL_8621436, EPI_ISL_8621437, EPI_ISL_8621438, EPI_ISL_8621439, EPI_ISL_8621440			
see above	UNIDADE DE PRONTO ATENDIMENTO DE RUSSAS	Analytical Competence Molecular Epidemiology Lab/ACME, Oswaldo Cruz Foundation, Ceara (FIOCRUZ CE)	Carlos Leonardo de Aragao Araujo; Cleber Furtado Aksenen; Fabio Miyajima; Fernando Braga Stehling; Igor Oliveira Duarte & Nicole Silva França on behalf of COVID-19 FIOCRUZ Genomic Network; Igor Oliveira Duarte on behalf & Nicole Silva França of COVID-19 FIOCRUZ Genomic Network; Jamille Maria Mendes Bezerra; Joaquim Cesar do Nascimento Sousa Junior; Pedro Miguel Carneiro Jeronimo; Suzana Porto Almeida; Thais Ferreira de Oliveira; Thais de Oliveira Costa; Ticiane Cavalcante de Souza; Veridiana Pessoa Miyajima
EPI_ISL_8721864	UNIDADE DE PRONTO ATENDIMENTO DRA ZILDA ARNS	Instituto Butantan	Antonio Jorge Martins; Claudia Renata dos Santos Barros; David Schlesinger; Debora Botequio Moretti; Dimas Tadeu Covas; Elaine Cristina Marqueze; Elaine Vieira Santos; Evandra Strazza Rodrigues; Heidge Fukumasu; Jayme Augusto de Souza-Neto; José Salvatore Leister Patané; Luiz Alcantara; Luiz Lehmann Coutinho; Maria Carolina Elias; Maurício Lacerda Nogueira; Rafael dos Santos Bezerra; Raul Machado Neto; Rejane Maria Tommasini Grotto; Ricardo Haddad; Sandra Coccuzzo Sampaio Vessoni; Simone Kashima; Svetoslav Nanev Slavov; Vincent Louis Viala
EPI_ISL_8353553, EPI_ISL_8721237, EPI_ISL_8721251, EPI_ISL_8721320, EPI_ISL_8721657, EPI_ISL_8721693, EPI_ISL_8721990			
see above	UNIDADE DE PRONTO ATENDIMENTO MASSAGUACU UPA NORTE CARAG	Instituto Butantan	Antonio Jorge Martins; Claudia Renata dos Santos Barros; David Schlesinger; Debora Botequio Moretti; Dimas Tadeu Covas; Elaine Cristina Marqueze; Elaine Vieira Santos; Evandra Strazza Rodrigues; Heidge Fukumasu; Jayme Augusto de Souza-Neto; José Salvatore Leister Patané; Luiz Alcantara; Luiz Lehmann Coutinho; Maria Carolina Elias; Maurício Lacerda Nogueira; Rafael dos Santos Bezerra; Raul Machado Neto; Rejane Maria Tommasini Grotto; Ricardo Haddad; Sandra Coccuzzo Sampaio Vessoni; Simone Kashima; Svetoslav Nanev Slavov; Vincent Louis Viala
EPI_ISL_8721456	UNIDADE DE PRONTO ATENDIMENTO UPA 24 HORAS	Instituto Butantan	Antonio Jorge Martins; Claudia Renata dos Santos Barros; David Schlesinger; Debora Botequio Moretti; Dimas Tadeu Covas; Elaine Cristina Marqueze; Elaine Vieira Santos; Evandra Strazza Rodrigues; Heidge Fukumasu; Jayme Augusto de Souza-Neto; José Salvatore Leister Patané; Luiz Alcantara; Luiz Lehmann Coutinho; Maria Carolina Elias; Maurício Lacerda Nogueira; Rafael dos Santos Bezerra; Raul Machado Neto; Rejane Maria Tommasini Grotto; Ricardo Haddad; Sandra Coccuzzo Sampaio Vessoni; Simone Kashima; Svetoslav Nanev Slavov; Vincent Louis Viala
EPI_ISL_8721346, EPI_ISL_8721500,	UNIDADE DE PRONTO	Instituto Butantan	Antonio Jorge Martins; Claudia Renata dos Santos Barros; David Schlesinger; Debora Botequio Moretti; Dimas Tadeu Covas; Elaine Cristina Marqueze; Elaine Vieira Santos; Evandra Strazza Rodrigues; Heidge Fukumasu; Jayme Augusto de Souza-Neto; José Salvatore Leister Patané;

EPI_ISL_8721534, EPI_ISL_8721689, EPI_ISL_8722000	ATENDIMENTO UPA DRA ANA OLIVIA BENTIVOGLIO		Luiz Alcantara; Luiz Lehmann Coutinho; Maria Carolina Elias; Maurício Lacerda Nogueira; Rafael dos Santos Bezerra; Raul Machado Neto; Rejane Maria Tommasini Grotto; Ricardo Haddad; Sandra Coccuzzo Sampaio Vessoni; Simone Kashima; Svetoslav Nanev Slavov; Vincent Louis Viala
EPI_ISL_8353609, EPI_ISL_8721938	UNIDADE DE PRONTO ATENDIMENTO UPA SUL CARAGUATATUBA	Instituto Butantan	Antonio Jorge Martins; Claudia Renata dos Santos Barros; David Schlesinger; Debora Botequio Moretti; Dimas Tadeu Covas; Elaine Cristina Marqueze; Elaine Vieira Santos; Evandra Strazza Rodrigues; Heidge Fukumasu; Jayme Augusto de Souza-Neto; José Salvatore Leister Patané; Luiz Alcantara; Luiz Lehmann Coutinho; Maria Carolina Elias; Maurício Lacerda Nogueira; Rafael dos Santos Bezerra; Raul Machado Neto; Rejane Maria Tommasini Grotto; Ricardo Haddad; Sandra Coccuzzo Sampaio Vessoni; Simone Kashima; Svetoslav Nanev Slavov; Vincent Louis Viala
EPI_ISL_8721345	UNIDADE DE SAUDE DA FAMILIA DR RAPHAEL	Instituto Butantan	Antonio Jorge Martins; Claudia Renata dos Santos Barros; David Schlesinger; Debora Botequio Moretti; Dimas Tadeu Covas; Elaine Cristina Marqueze; Elaine Vieira Santos; Evandra Strazza Rodrigues; Heidge Fukumasu; Jayme Augusto de Souza-Neto; José Salvatore Leister Patané; Luiz Alcantara; Luiz Lehmann Coutinho; Maria Carolina Elias; Maurício Lacerda Nogueira; Rafael dos Santos Bezerra; Raul Machado Neto; Rejane Maria Tommasini Grotto; Ricardo Haddad; Sandra Coccuzzo Sampaio Vessoni; Simone Kashima; Svetoslav Nanev Slavov; Vincent Louis Viala
EPI_ISL_8465110	UNIDADE MISTA DE AVANHANDAVA	Instituto Butantan	Antonio Jorge Martins; Claudia Renata dos Santos Barros; David Schlesinger; Debora Botequio Moretti; Dimas Tadeu Covas; Elaine Cristina Marqueze; Elaine Vieira Santos; Evandra Strazza Rodrigues; Heidge Fukumasu; Jayme Augusto de Souza-Neto; José Salvatore Leister Patané; Luiz Alcantara; Luiz Lehmann Coutinho; Maria Carolina Elias; Maurício Lacerda Nogueira; Rafael dos Santos Bezerra; Raul Machado Neto; Rejane Maria Tommasini Grotto; Ricardo Haddad; Sandra Coccuzzo Sampaio Vessoni; Simone Kashima; Svetoslav Nanev Slavov; Vincent Louis Viala
EPI_ISL_8721539	UNIDADE MISTA DE SAUDE DE EMBUGUACU	Instituto Butantan	Antonio Jorge Martins; Claudia Renata dos Santos Barros; David Schlesinger; Debora Botequio Moretti; Dimas Tadeu Covas; Elaine Cristina Marqueze; Elaine Vieira Santos; Evandra Strazza Rodrigues; Heidge Fukumasu; Jayme Augusto de Souza-Neto; José Salvatore Leister Patané; Luiz Alcantara; Luiz Lehmann Coutinho; Maria Carolina Elias; Maurício Lacerda Nogueira; Rafael dos Santos Bezerra; Raul Machado Neto; Rejane Maria Tommasini Grotto; Ricardo Haddad; Sandra Coccuzzo Sampaio Vessoni; Simone Kashima; Svetoslav Nanev Slavov; Vincent Louis Viala
EPI_ISL_8721476, EPI_ISL_8721621, EPI_ISL_8721894, EPI_ISL_8722005	UNIDADE REFERENCIAL SUDOESTE	Instituto Butantan	Antonio Jorge Martins; Claudia Renata dos Santos Barros; David Schlesinger; Debora Botequio Moretti; Dimas Tadeu Covas; Elaine Cristina Marqueze; Elaine Vieira Santos; Evandra Strazza Rodrigues; Heidge Fukumasu; Jayme Augusto de Souza-Neto; José Salvatore Leister Patané; Luiz Alcantara; Luiz Lehmann Coutinho; Maria Carolina Elias; Maurício Lacerda Nogueira; Rafael dos Santos Bezerra; Raul Machado Neto; Rejane Maria Tommasini Grotto; Ricardo Haddad; Sandra Coccuzzo Sampaio Vessoni; Simone Kashima; Svetoslav Nanev Slavov; Vincent Louis Viala
EPI_ISL_8721607, EPI_ISL_8721721, EPI_ISL_8721759, EPI_ISL_8721764, EPI_ISL_8721812, EPI_ISL_8721956	UNIDADE RESPIRATORIA NOVA HORTOLANDIA	Instituto Butantan	Antonio Jorge Martins; Claudia Renata dos Santos Barros; David Schlesinger; Debora Botequio Moretti; Dimas Tadeu Covas; Elaine Cristina Marqueze; Elaine Vieira Santos; Evandra Strazza Rodrigues; Heidge Fukumasu; Jayme Augusto de Souza-Neto; José Salvatore Leister Patané; Luiz Alcantara; Luiz Lehmann Coutinho; Maria Carolina Elias; Maurício Lacerda Nogueira; Rafael dos Santos Bezerra; Raul Machado Neto; Rejane Maria Tommasini Grotto; Ricardo Haddad; Sandra Coccuzzo Sampaio Vessoni; Simone Kashima; Svetoslav Nanev Slavov; Vincent Louis Viala
EPI_ISL_8721473, EPI_ISL_8721580, EPI_ISL_8721909, EPI_ISL_8721935	UPA 13 DE MAIO – DR LUIS ATILIO LOSI VIANA	Instituto Butantan	Antonio Jorge Martins; Claudia Renata dos Santos Barros; David Schlesinger; Debora Botequio Moretti; Dimas Tadeu Covas; Elaine Cristina Marqueze; Elaine Vieira Santos; Evandra Strazza Rodrigues; Heidge Fukumasu; Jayme Augusto de Souza-Neto; José Salvatore Leister Patané; Luiz Alcantara; Luiz Lehmann Coutinho; Maria Carolina Elias; Maurício Lacerda Nogueira; Rafael dos Santos Bezerra; Raul Machado Neto; Rejane Maria Tommasini Grotto; Ricardo Haddad; Sandra Coccuzzo Sampaio Vessoni; Simone Kashima; Svetoslav Nanev Slavov; Vincent Louis Viala
EPI_ISL_7898478	UPA 13 de maio	Instituto Butantan	Antonio Jorge Martins; Claudia Renata dos Santos Barros; David Schlesinger; Debora Botequio Moretti; Dimas Tadeu Covas; Elaine Cristina Marqueze; Elaine Vieira Santos; Evandra Strazza Rodrigues; Heidge Fukumasu; Jayme Augusto de Souza-Neto; José Salvatore Leister Patané; Luiz Alcantara; Luiz Lehmann Coutinho; Maria Carolina Elias; Maurício Lacerda Nogueira; Rafael dos Santos Bezerra; Raul Machado Neto; Rejane Maria Tommasini Grotto; Ricardo Haddad; Sandra Coccuzzo Sampaio Vessoni; Simone Kashima; Svetoslav Nanev Slavov; Vincent Louis Viala
EPI_ISL_8721384	UPA 24 HORAS ANA JACINTA	Instituto Butantan	Antonio Jorge Martins; Claudia Renata dos Santos Barros; David Schlesinger; Debora Botequio Moretti; Dimas Tadeu Covas; Elaine Cristina Marqueze; Elaine Vieira Santos; Evandra Strazza Rodrigues; Heidge Fukumasu; Jayme Augusto de Souza-Neto; José Salvatore Leister Patané; Luiz Alcantara; Luiz Lehmann Coutinho; Maria Carolina Elias; Maurício Lacerda Nogueira; Rafael dos Santos Bezerra; Raul Machado Neto; Rejane Maria Tommasini Grotto; Ricardo Haddad; Sandra Coccuzzo Sampaio Vessoni; Simone Kashima; Svetoslav Nanev Slavov; Vincent Louis Viala
EPI_ISL_8721795	UPA 24 HORAS CENTRO	Instituto Butantan	Antonio Jorge Martins; Claudia Renata dos Santos Barros; David Schlesinger; Debora Botequio Moretti; Dimas Tadeu Covas; Elaine Cristina Marqueze; Elaine Vieira Santos; Evandra Strazza Rodrigues; Heidge Fukumasu; Jayme Augusto de Souza-Neto; José Salvatore Leister Patané; Luiz Alcantara; Luiz Lehmann Coutinho; Maria Carolina Elias; Maurício Lacerda Nogueira; Rafael dos Santos Bezerra; Raul Machado Neto; Rejane Maria Tommasini Grotto; Ricardo Haddad; Sandra Coccuzzo Sampaio Vessoni; Simone Kashima; Svetoslav Nanev Slavov; Vincent Louis Viala
EPI_ISL_8721977	UPA 24 HS UNIDADE DE PRONTO ATENDIMENTO DR MARIO NUNES MIRAN	Instituto Butantan	Antonio Jorge Martins; Claudia Renata dos Santos Barros; David Schlesinger; Debora Botequio Moretti; Dimas Tadeu Covas; Elaine Cristina Marqueze; Elaine Vieira Santos; Evandra Strazza Rodrigues; Heidge Fukumasu; Jayme Augusto de Souza-Neto; José Salvatore Leister Patané; Luiz Alcantara; Luiz Lehmann Coutinho; Maria Carolina Elias; Maurício Lacerda Nogueira; Rafael dos Santos Bezerra; Raul Machado Neto; Rejane Maria Tommasini Grotto; Ricardo Haddad; Sandra Coccuzzo Sampaio Vessoni; Simone Kashima; Svetoslav Nanev Slavov; Vincent Louis Viala
EPI_ISL_8721769, EPI_ISL_8721797, EPI_ISL_8721841	UPA ALTO DA PONTE	Instituto Butantan	Antonio Jorge Martins; Claudia Renata dos Santos Barros; David Schlesinger; Debora Botequio Moretti; Dimas Tadeu Covas; Elaine Cristina Marqueze; Elaine Vieira Santos; Evandra Strazza Rodrigues; Heidge Fukumasu; Jayme Augusto de Souza-Neto; José Salvatore Leister Patané; Luiz Alcantara; Luiz Lehmann Coutinho; Maria Carolina Elias; Maurício Lacerda Nogueira; Rafael dos Santos Bezerra; Raul Machado Neto; Rejane Maria Tommasini Grotto; Ricardo Haddad; Sandra Coccuzzo Sampaio Vessoni; Simone Kashima; Svetoslav Nanev Slavov; Vincent Louis Viala
EPI_ISL_8317863	UPA ATALAIA DRA RITA DE CASSIA SORIO	Instituto Butantan	Antonio Jorge Martins; Claudia Renata dos Santos Barros; David Schlesinger; Debora Botequio Moretti; Dimas Tadeu Covas; Elaine Cristina Marqueze; Elaine Vieira Santos; Evandra Strazza Rodrigues; Heidge Fukumasu; Jayme Augusto de Souza-Neto; José Salvatore Leister Patané; Luiz Alcantara; Luiz Lehmann Coutinho; Maria Carolina Elias; Maurício Lacerda Nogueira; Rafael dos Santos Bezerra; Raul Machado Neto; Rejane Maria Tommasini Grotto; Ricardo Haddad; Sandra Coccuzzo Sampaio Vessoni; Simone Kashima; Svetoslav Nanev Slavov; Vincent Louis Viala
EPI_ISL_8721821	UPA CAMPO DOS ALEMAES	Instituto Butantan	Antonio Jorge Martins; Claudia Renata dos Santos Barros; David Schlesinger; Debora Botequio Moretti; Dimas Tadeu Covas; Elaine Cristina Marqueze; Elaine Vieira Santos; Evandra Strazza Rodrigues; Heidge Fukumasu; Jayme Augusto de Souza-Neto; José Salvatore Leister Patané; Luiz Alcantara; Luiz Lehmann Coutinho; Maria Carolina Elias; Maurício Lacerda Nogueira; Rafael dos Santos Bezerra; Raul Machado Neto; Rejane Maria Tommasini Grotto; Ricardo Haddad; Sandra Coccuzzo Sampaio Vessoni; Simone Kashima; Svetoslav Nanev Slavov; Vincent Louis Viala
EPI_ISL_8353579, EPI_ISL_8353580, EPI_ISL_8353581	UPA DR AKIRA TADA	Instituto Butantan	Antonio Jorge Martins; Claudia Renata dos Santos Barros; David Schlesinger; Debora Botequio Moretti; Dimas Tadeu Covas; Elaine Cristina Marqueze; Elaine Vieira Santos; Evandra Strazza Rodrigues; Heidge Fukumasu; Jayme Augusto de Souza-Neto; José Salvatore Leister Patané; Luiz Alcantara; Luiz Lehmann Coutinho; Maria Carolina Elias; Maurício Lacerda Nogueira; Rafael dos Santos Bezerra; Raul Machado Neto; Rejane Maria Tommasini Grotto; Ricardo Haddad; Sandra Coccuzzo Sampaio Vessoni; Simone Kashima; Svetoslav Nanev Slavov; Vincent Louis Viala
EPI_ISL_8184832	UPA II JUNDIAI	Instituto Butantan	Antonio Jorge Martins; Claudia Renata dos Santos Barros; David Schlesinger; Debora Botequio Moretti; Dimas Tadeu Covas; Elaine Cristina Marqueze; Elaine Vieira Santos; Evandra Strazza Rodrigues; Heidge Fukumasu; Jayme Augusto de Souza-Neto; José Salvatore Leister Patané; Luiz Alcantara; Luiz Lehmann Coutinho; Maria Carolina Elias; Maurício Lacerda Nogueira; Rafael dos Santos Bezerra; Raul Machado Neto; Rejane Maria Tommasini Grotto; Ricardo Haddad; Sandra Coccuzzo Sampaio Vessoni; Simone Kashima; Svetoslav Nanev Slavov; Vincent Louis Viala
EPI_ISL_8721300	UPA PORTE II 24HS JD CEREJEIRAS ATIBAIA	Instituto Butantan	Antonio Jorge Martins; Claudia Renata dos Santos Barros; David Schlesinger; Debora Botequio Moretti; Dimas Tadeu Covas; Elaine Cristina Marqueze; Elaine Vieira Santos; Evandra Strazza Rodrigues; Heidge Fukumasu; Jayme Augusto de Souza-Neto; José Salvatore Leister Patané; Luiz Alcantara; Luiz Lehmann Coutinho; Maria Carolina Elias; Maurício Lacerda Nogueira; Rafael dos Santos Bezerra; Raul Machado Neto; Rejane Maria Tommasini Grotto; Ricardo Haddad; Sandra Coccuzzo Sampaio Vessoni; Simone Kashima; Svetoslav Nanev Slavov; Vincent Louis Viala
EPI_ISL_8721942	UPA VILA SANTA CATARINA	Instituto Butantan	Antonio Jorge Martins; Claudia Renata dos Santos Barros; David Schlesinger; Debora Botequio Moretti; Dimas Tadeu Covas; Elaine Cristina Marqueze; Elaine Vieira Santos; Evandra Strazza Rodrigues; Heidge Fukumasu; Jayme Augusto de Souza-Neto; José Salvatore Leister Patané; Luiz Alcantara; Luiz Lehmann Coutinho; Maria Carolina Elias; Maurício Lacerda Nogueira; Rafael dos Santos Bezerra; Raul Machado Neto; Rejane Maria Tommasini Grotto; Ricardo Haddad; Sandra Coccuzzo Sampaio Vessoni; Simone Kashima; Svetoslav Nanev Slavov; Vincent Louis Viala
EPI_ISL_8465101, EPI_ISL_8465102, EPI_ISL_8465107, EPI_ISL_8465108, EPI_ISL_8465113, EPI_ISL_8465115, EPI_ISL_8721228, EPI_ISL_8721412, EPI_ISL_8721485, EPI_ISL_8721577			
see above	USF CESAR NETO BOTUCATU	Instituto Butantan	Antonio Jorge Martins; Claudia Renata dos Santos Barros; David Schlesinger; Debora Botequio Moretti; Dimas Tadeu Covas; Elaine Cristina Marqueze; Elaine Vieira Santos; Evandra Strazza Rodrigues; Heidge Fukumasu; Jayme Augusto de Souza-Neto; José Salvatore Leister Patané; Luiz Alcantara; Luiz Lehmann Coutinho; Maria Carolina Elias; Maurício Lacerda Nogueira; Rafael dos Santos Bezerra; Raul Machado Neto; Rejane Maria Tommasini Grotto; Ricardo Haddad; Sandra Coccuzzo Sampaio Vessoni; Simone Kashima; Svetoslav Nanev Slavov; Vincent Louis Viala
EPI_ISL_8721758	USF COHAB IV	Instituto Butantan	Antonio Jorge Martins; Claudia Renata dos Santos Barros; David Schlesinger; Debora Botequio Moretti; Dimas Tadeu Covas; Elaine Cristina Marqueze; Elaine Vieira Santos; Evandra Strazza Rodrigues; Heidge Fukumasu; Jayme Augusto de Souza-Neto; José Salvatore Leister Patané; Luiz Alcantara; Luiz Lehmann Coutinho; Maria Carolina Elias; Maurício Lacerda Nogueira; Rafael dos Santos Bezerra; Raul Machado Neto; Rejane Maria Tommasini Grotto; Ricardo Haddad; Sandra Coccuzzo Sampaio Vessoni; Simone Kashima; Svetoslav Nanev Slavov; Vincent Louis Viala
EPI_ISL_8721998	USF DR JOSE MARTINHO PALERMO II	Instituto Butantan	Antonio Jorge Martins; Claudia Renata dos Santos Barros; David Schlesinger; Debora Botequio Moretti; Dimas Tadeu Covas; Elaine Cristina Marqueze; Elaine Vieira Santos; Evandra Strazza Rodrigues; Heidge Fukumasu; Jayme Augusto de Souza-Neto; José Salvatore Leister Patané; Luiz Alcantara; Luiz Lehmann Coutinho; Maria Carolina Elias; Maurício Lacerda Nogueira; Rafael dos Santos Bezerra; Raul Machado Neto; Rejane Maria Tommasini Grotto; Ricardo Haddad; Sandra Coccuzzo Sampaio Vessoni; Simone Kashima; Svetoslav Nanev Slavov; Vincent Louis Viala
EPI_ISL_8721535	USF JARDIM FLAMINGO	Instituto Butantan	Antonio Jorge Martins; Claudia Renata dos Santos Barros; David Schlesinger; Debora Botequio Moretti; Dimas Tadeu Covas; Elaine Cristina Marqueze; Elaine Vieira Santos; Evandra Strazza Rodrigues; Heidge Fukumasu; Jayme Augusto de Souza-Neto; José Salvatore Leister Patané; Luiz Alcantara; Luiz Lehmann Coutinho; Maria Carolina Elias; Maurício Lacerda Nogueira; Rafael dos Santos Bezerra; Raul Machado Neto; Rejane Maria Tommasini Grotto; Ricardo Haddad; Sandra Coccuzzo Sampaio Vessoni; Simone Kashima; Svetoslav Nanev Slavov; Vincent Louis Viala
EPI_ISL_8721350	USF ROSALIA	Instituto Butantan	Antonio Jorge Martins; Claudia Renata dos Santos Barros; David Schlesinger; Debora Botequio Moretti; Dimas Tadeu Covas; Elaine Cristina Marqueze; Elaine Vieira Santos; Evandra Strazza Rodrigues; Heidge Fukumasu; Jayme Augusto de Souza-Neto; José Salvatore Leister Patané; Luiz Alcantara; Luiz Lehmann Coutinho; Maria Carolina Elias; Maurício Lacerda Nogueira; Rafael dos Santos Bezerra; Raul Machado Neto; Rejane Maria Tommasini Grotto; Ricardo Haddad; Sandra Coccuzzo Sampaio Vessoni; Simone Kashima; Svetoslav Nanev Slavov; Vincent Louis Viala
EPI_ISL_8721439, EPI_ISL_8721710, EPI_ISL_8721831	USF SANTA ANTONIETA II	Instituto Butantan	Antonio Jorge Martins; Claudia Renata dos Santos Barros; David Schlesinger; Debora Botequio Moretti; Dimas Tadeu Covas; Elaine Cristina Marqueze; Elaine Vieira Santos; Evandra Strazza Rodrigues; Heidge Fukumasu; Jayme Augusto de Souza-Neto; José Salvatore Leister Patané; Luiz Alcantara; Luiz Lehmann Coutinho; Maria Carolina Elias; Maurício Lacerda Nogueira; Rafael dos Santos Bezerra; Raul Machado Neto; Rejane Maria Tommasini Grotto; Ricardo Haddad; Sandra Coccuzzo Sampaio Vessoni; Simone Kashima; Svetoslav Nanev Slavov; Vincent Louis Viala
EPI_ISL_2535116, EPI_ISL_2691575, EPI_ISL_2691619	Unidade de apoio ao diagnostico da COVID - UNADIG	Bioinformatics Laboratory / LNCC	Alessandra P Lamarca; Alexandra L Gerber; Amilcar Tanuri; Ana Paula de C Guimaraes; Ana Tereza R Vasconcelos; Andrea Cony Cavalcanti; Caio Luiz Pereira Ribeiro; Cassia Alves; Cintia Policarpo; Claudia Maria Braga de Mello; Cristiane Gomes da Silva; Diana Mariani; Douglas Terra Machado; Flavio Dias da Silva; Gleidson da Silva de Oliveira; Leandro Magalhaes de Souza; Liliane Cavalcante; Luiz G P de Almeida; Marcio Henrique de Oliveira Garcia; Mario Sergio Ribeiro; Ronaldo da Silva F Jr; Silvia Carvalho
EPI_ISL_8721790, EPI_ISL_8721978	VIGILANCIA EM SAUDE ITAPETININGA	Instituto Butantan	Antonio Jorge Martins; Claudia Renata dos Santos Barros; David Schlesinger; Debora Botequio Moretti; Dimas Tadeu Covas; Elaine Cristina Marqueze; Elaine Vieira Santos; Evandra Strazza Rodrigues; Heidge Fukumasu; Jayme Augusto de Souza-Neto; José Salvatore Leister Patané; Luiz Alcantara; Luiz Lehmann Coutinho; Maria Carolina Elias; Maurício Lacerda Nogueira; Rafael dos Santos Bezerra; Raul Machado Neto; Rejane Maria Tommasini Grotto; Ricardo Haddad; Sandra Coccuzzo Sampaio Vessoni; Simone Kashima; Svetoslav Nanev Slavov; Vincent Louis Viala
EPI_ISL_8353526	VIGILANCIA EPIDEMIOLOGICA - SERTAOZINHO	Instituto Butantan	Antonio Jorge Martins; Claudia Renata dos Santos Barros; David Schlesinger; Debora Botequio Moretti; Dimas Tadeu Covas; Elaine Cristina Marqueze; Elaine Vieira Santos; Evandra Strazza Rodrigues; Heidge Fukumasu; Jayme Augusto de Souza-Neto; José Salvatore Leister Patané; Luiz Alcantara; Luiz Lehmann Coutinho; Maria Carolina Elias; Maurício Lacerda Nogueira; Rafael dos Santos Bezerra; Raul Machado Neto; Rejane Maria Tommasini Grotto; Ricardo Haddad; Sandra Coccuzzo Sampaio Vessoni; Simone Kashima; Svetoslav Nanev Slavov; Vincent Louis Viala
EPI_ISL_8721922	VIGILANCIA EPIDEMIOLOGICA CONTROLE DOENCAS	Instituto Butantan	Antonio Jorge Martins; Claudia Renata dos Santos Barros; David Schlesinger; Debora Botequio Moretti; Dimas Tadeu Covas; Elaine Cristina Marqueze; Elaine Vieira Santos; Evandra Strazza Rodrigues; Heidge Fukumasu; Jayme Augusto de Souza-Neto; José Salvatore Leister Patané; Luiz Alcantara; Luiz Lehmann Coutinho; Maria Carolina Elias; Maurício Lacerda Nogueira; Rafael dos Santos Bezerra; Raul Machado Neto; Rejane Maria Tommasini Grotto; Ricardo Haddad; Sandra Coccuzzo Sampaio Vessoni; Simone Kashima; Svetoslav Nanev Slavov; Vincent Louis Viala
EPI_ISL_8353557	VIGILANCIA SANITARIA E VIG EPIDEMIOLOGICA DE ITAPEVI	Instituto Butantan	Antonio Jorge Martins; Claudia Renata dos Santos Barros; David Schlesinger; Debora Botequio Moretti; Dimas Tadeu Covas; Elaine Cristina Marqueze; Elaine Vieira Santos; Evandra Strazza Rodrigues; Heidge Fukumasu; Jayme Augusto de Souza-Neto; José Salvatore Leister Patané; Luiz Alcantara; Luiz Lehmann Coutinho; Maria Carolina Elias; Maurício Lacerda Nogueira; Rafael dos Santos Bezerra; Raul Machado Neto; Rejane Maria Tommasini Grotto; Ricardo Haddad; Sandra Coccuzzo Sampaio Vessoni; Simone Kashima; Svetoslav Nanev Slavov; Vincent Louis Viala
