# Supplementary figures and images for: SARS-CoV-2 reinfections with BA.1 (Omicron) variant among fully vaccinated individuals in northeastern Brazil
Source: PLoS Negl Trop Dis. 2022 Oct 3;16(10):e0010337. doi: 10.1371/journal.pntd.0010337 (PMC9560550; doi:10.1371/journal.pntd.0010337)

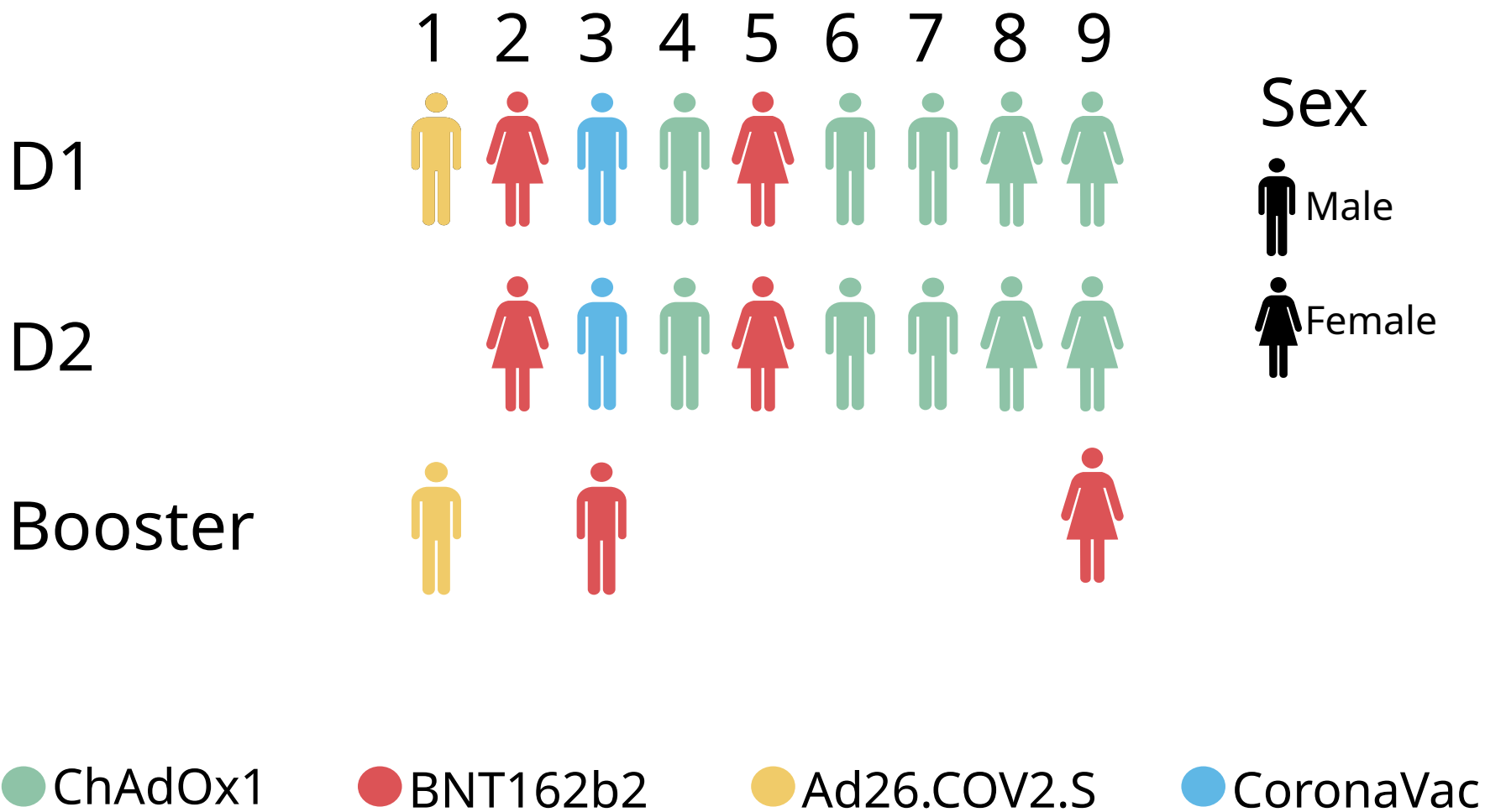

Supplement: S1 Fig — (PDF) [file pntd.0010337.s004.pdf]

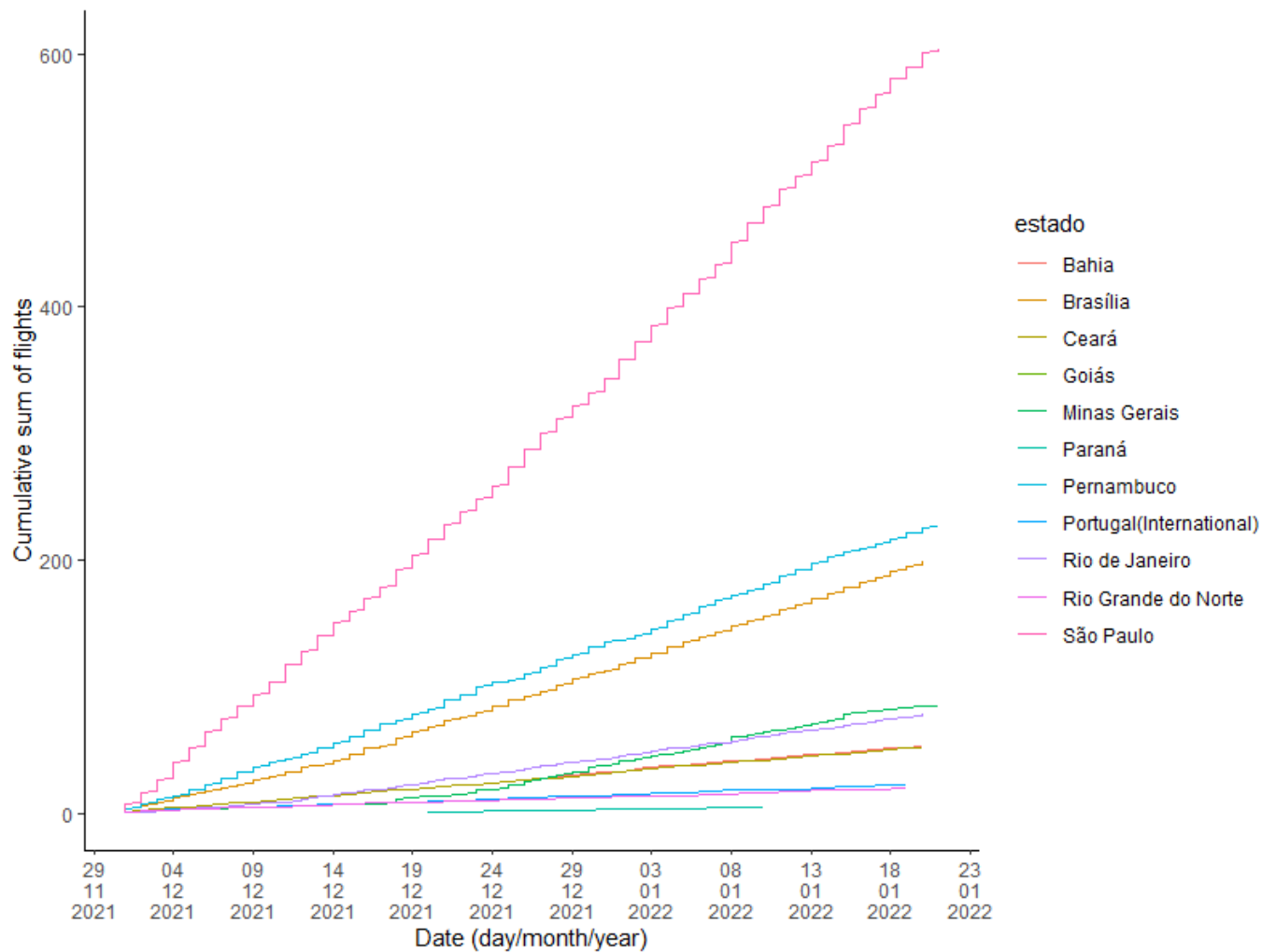

Supplement: S3 Fig — The order from the Brazilian federative unit with the most flights to Natal (from December 01, 2021 to January 20, 2022) from, to the lesser one, is: São Paulo (604 flights); Pernambuco (227 flights); Brasília (199 flights); Minas Gerais (85 flights); Rio de Janeiro (78 flights); Bahia (53 flights); Ceará (52 flights); Rio Grande do Norte (20 flights); Paraná (4 flights); Goiás (1 flight); and also Portugal with 22 international flights. (PDF) [file pntd.0010337.s006.pdf]
