# Supplementary material for: SARS-CoV-2 reinfections with BA.1 (Omicron) variant among fully vaccinated individuals in northeastern Brazil
Source: PLoS Negl Trop Dis. 2022 Oct 3;16(10):e0010337. doi: 10.1371/journal.pntd.0010337 (PMC9560550; doi:10.1371/journal.pntd.0010337)
Supplement: S2 Fig — The Omicron genomes sequenced here are indicated with the red tips. (PDF) [file pntd.0010337.s005.pdf]

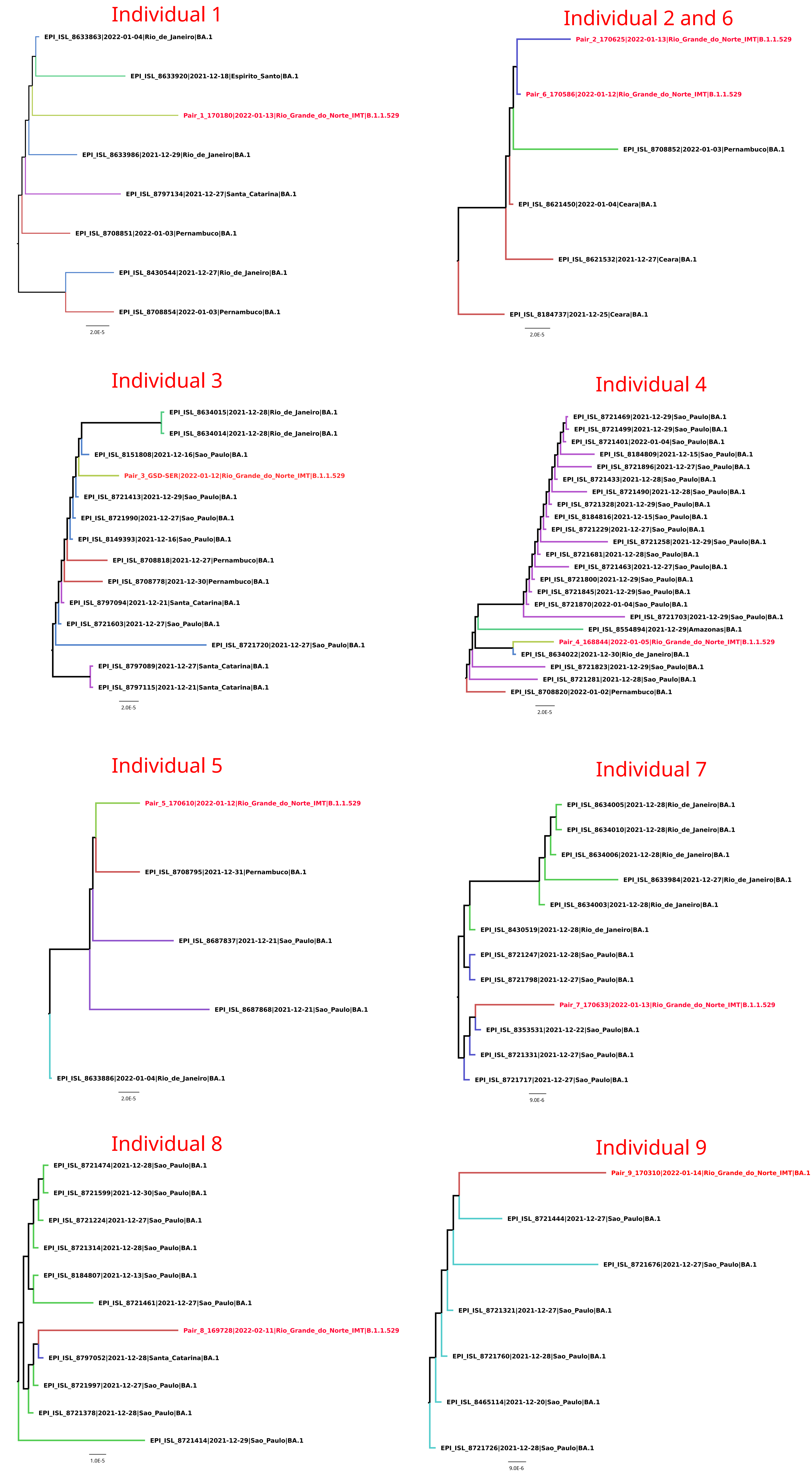
Individual 1
EPI_ISL_8633863|2022-01-04|Rio_de_Janeiro|BA.1
EPI_ISL_8633920|2021-12-18|Espirito_Santo|BA.1
Pair_1_170180|2022-01-13|Rio_Grande_do_Norte_IMT|B.1.1.529
EPI_ISL_8633986|2021-12-29|Rio_de_Janeiro|BA.1
EPI_ISL_8797134|2021-12-27|Santa_Catarina|BA.1
EPI_ISL_8708851|2022-01-03|Pernambuco|BA.1
EPI_ISL_8430544|2021-12-27|Rio_de_Janeiro|BA.1
EPI_ISL_8708854|2022-01-03|Pernambuco|BA.1
2.0E-5
Individual 2 and 6
Pair_2_170625|2022-01-13|Rio_Grande_do_Norte_IMT|B.1.1.529
Pair_6_170586|2022-01-12|Rio_Grande_do_Norte_IMT|B.1.1.529
EPI_ISL_8708852|2022-01-03|Pernambuco|BA.1
EPI_ISL_8621450|2022-01-04|Ceara|BA.1
EPI_ISL_8621532|2021-12-27|Ceara|BA.1
EPI_ISL_8184737|2021-12-25|Ceara|BA.1
2.0E-5
Individual 3
EPI_ISL_8634015|2021-12-28|Rio_de_Janeiro|BA.1
EPI_ISL_8634014|2021-12-28|Rio_de_Janeiro|BA.1
EPI_ISL_8151808|2021-12-16|Sao_Paulo|BA.1
Pair_3_GSD-SER|2022-01-12|Rio_Grande_do_Norte_IMT|B.1.1.529
EPI_ISL_8721413|2021-12-29|Sao_Paulo|BA.1
EPI_ISL_8721990|2021-12-27|Sao_Paulo|BA.1
EPI_ISL_8149393|2021-12-16|Sao_Paulo|BA.1
EPI_ISL_8708818|2021-12-27|Pernambuco|BA.1
EPI_ISL_8708778|2021-12-30|Pernambuco|BA.1
EPI_ISL_8797094|2021-12-21|Santa_Catarina|BA.1
EPI_ISL_8721603|2021-12-27|Sao_Paulo|BA.1
EPI_ISL_8721720|2021-12-27|Sao_Paulo|BA.1
EPI_ISL_8797089|2021-12-27|Santa_Catarina|BA.1
EPI_ISL_8797115|2021-12-21|Santa_Catarina|BA.1
2.0E-5
Individual 4
EPI_ISL_8721469|2021-12-29|Sao_Paulo|BA.1
EPI_ISL_8721499|2021-12-29|Sao_Paulo|BA.1
EPI_ISL_8721401|2022-01-04|Sao_Paulo|BA.1
EPI_ISL_8184809|2021-12-15|Sao_Paulo|BA.1
EPI_ISL_8721896|2021-12-27|Sao_Paulo|BA.1
EPI_ISL_8721433|2021-12-28|Sao_Paulo|BA.1
EPI_ISL_8721490|2021-12-28|Sao_Paulo|BA.1
EPI_ISL_8721328|2021-12-29|Sao_Paulo|BA.1
EPI_ISL_8184816|2021-12-15|Sao_Paulo|BA.1
EPI_ISL_8721229|2021-12-27|Sao_Paulo|BA.1
EPI_ISL_8721258|2021-12-29|Sao_Paulo|BA.1
EPI_ISL_8721681|2021-12-28|Sao_Paulo|BA.1
EPI_ISL_8721463|2021-12-27|Sao_Paulo|BA.1
EPI_ISL_8721800|2021-12-29|Sao_Paulo|BA.1
EPI_ISL_8721845|2021-12-29|Sao_Paulo|BA.1
EPI_ISL_8721870|2022-01-04|Sao_Paulo|BA.1
EPI_ISL_8721703|2021-12-29|Sao_Paulo|BA.1
EPI_ISL_8554894|2021-12-29|Amazonas|BA.1
Pair_4_168844|2022-01-05|Rio_Grande_do_Norte_IMT|B.1.1.529
EPI_ISL_8634022|2021-12-30|Rio_de_Janeiro|BA.1
EPI_ISL_8721823|2021-12-29|Sao_Paulo|BA.1
EPI_ISL_8721281|2021-12-28|Sao_Paulo|BA.1
EPI_ISL_8708820|2022-01-02|Pernambuco|BA.1
2.0E-5
Individual 5
Pair_5_170610|2022-01-12|Rio_Grande_do_Norte_IMT|B.1.1.529
EPI_ISL_8708795|2021-12-31|Pernambuco|BA.1
EPI_ISL_8687837|2021-12-21|Sao_Paulo|BA.1
EPI_ISL_8687868|2021-12-21|Sao_Paulo|BA.1
EPI_ISL_8633886|2022-01-04|Rio_de_Janeiro|BA.1
2.0E-5
Individual 7
EPI_ISL_8634005|2021-12-28|Rio_de_Janeiro|BA.1
EPI_ISL_8634010|2021-12-28|Rio_de_Janeiro|BA.1
EPI_ISL_8634006|2021-12-28|Rio_de_Janeiro|BA.1
EPI_ISL_8633984|2021-12-27|Rio_de_Janeiro|BA.1
EPI_ISL_8634003|2021-12-28|Rio_de_Janeiro|BA.1
EPI_ISL_8430519|2021-12-28|Rio_de_Janeiro|BA.1
EPI_ISL_8721247|2021-12-28|Sao_Paulo|BA.1
EPI_ISL_8721798|2021-12-27|Sao_Paulo|BA.1
Pair_7_170633|2022-01-13|Rio_Grande_do_Norte_IMT|B.1.1.529
EPI_ISL_8353531|2021-12-22|Sao_Paulo|BA.1
EPI_ISL_8721331|2021-12-27|Sao_Paulo|BA.1
EPI_ISL_8721717|2021-12-27|Sao_Paulo|BA.1
9.0E-6
Individual 8
EPI_ISL_8721474|2021-12-28|Sao_Paulo|BA.1
EPI_ISL_8721599|2021-12-30|Sao_Paulo|BA.1
EPI_ISL_8721224|2021-12-27|Sao_Paulo|BA.1
EPI_ISL_8721314|2021-12-28|Sao_Paulo|BA.1
EPI_ISL_8184807|2021-12-13|Sao_Paulo|BA.1
EPI_ISL_8721461|2021-12-27|Sao_Paulo|BA.1
Pair_8_169728|2022-02-11|Rio_Grande_do_Norte_IMT|B.1.1.529
EPI_ISL_8797052|2021-12-28|Santa_Catarina|BA.1
EPI_ISL_8721997|2021-12-27|Sao_Paulo|BA.1
EPI_ISL_8721378|2021-12-28|Sao_Paulo|BA.1
EPI_ISL_8721414|2021-12-29|Sao_Paulo|BA.1
1.0E-5
Individual 9
Pair_9_170310|2022-01-14|Rio_Grande_do_Norte_IMT|BA.1
EPI_ISL_8721444|2021-12-27|Sao_Paulo|BA.1
EPI_ISL_8721676|2021-12-27|Sao_Paulo|BA.1
EPI_ISL_8721321|2021-12-27|Sao_Paulo|BA.1
EPI_ISL_8721760|2021-12-28|Sao_Paulo|BA.1
EPI_ISL_8465114|2021-12-20|Sao_Paulo|BA.1
EPI_ISL_8721726|2021-12-28|Sao_Paulo|BA.1
9.0E-6
